# Thermally activated delayed fluorescence and high-contrast mechanochromism of anthrone-based donor–acceptor systems

**DOI:** 10.3389/fchem.2023.1248267

**Published:** 2023-08-31

**Authors:** Pagidi Sudhakar, Alexandra Slawin, Eli Zysman-Colman

**Affiliations:** Organic Semiconductor Centre, EaStCHEM School of Chemistry, University of St Andrews, St Andrews, United Kingdom

**Keywords:** thermally activated delayed fluorescence, anthrone, mechanochromism, donor–acceptor, red

## Abstract

The development of materials that emit in the deep-red to near-infrared region of the spectrum has attracted significant attention due to their potential as optical sensing and imaging reagents in biology. Herein, we report the synthesis and optoelectronic characterization of four anthraquinone-based emitters, **T-tBuCz-AQ**, **T-MeOCz-AQ**, **C-tBuCz-AQ**, and **C-MeOCz-AQ**, and two pyrazoloanthrone-based emitters, **tBuCz-PA** and **DMAC-PA**. Depending on the donor, these compounds emit in the spectral range between 640 and 750 nm in the neat film, while the emission of the 10 wt% doped films in poly(methyl methacrylate) (PMMA) is blue-shifted between 600 and 700 nm and has low photoluminescence quantum yields between 2.6% and 6.6%. Of these compounds, **T-tBuCz-AQ**, **T-MeOCz-AQ**, and **C-tBuCz-AQ** exhibited thermally activated delayed fluorescence (TADF) in 10 wt% doped films in PMMA, while the crystals of **T-tBuCz-AQ** also showed TADF. Compound **tBuCz-PA** showed a high-contrast and reversible photoluminescence (PL) response upon mechanical grinding and hexane fuming.

## Graphical Abstract

**Figure:**
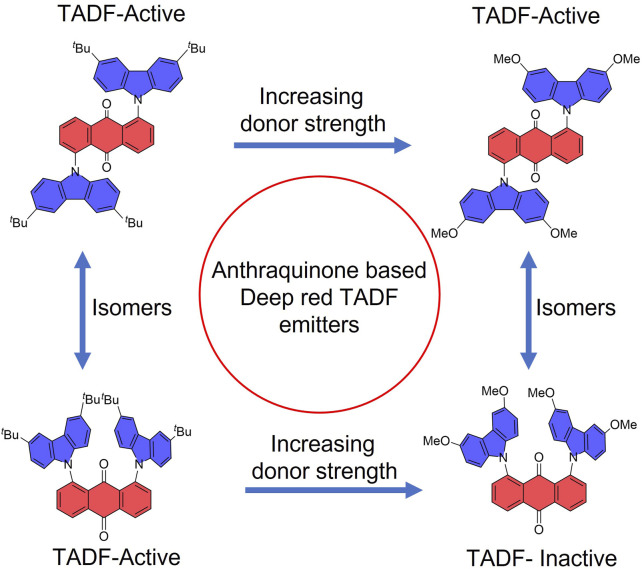

## Introduction

Organic emissive materials have gained significant attention in view of their use in myriad applications from organic light-emitting diodes (OLEDs) (Wong and Zysman-Colman, 2017) to bioimaging (Yang et al., 2022), sensors (Wu et al., 2017), and organic lasers (Kuehne and Gather, 2016). Recently, the discovery that organic compounds that emit via thermally activated delayed fluorescence (TADF) could, similar to phosphorescent metal complexes, harvest 100% of electrically generated excitons to produce light has initiated a tsunami of research activities in OLEDs (Uoyama et al., 2012). Typically, TADF emitters comprised electron donor and acceptor moieties that are weakly electronically coupled, usually by means of a strong twisted conformation, which results in compounds having a small singlet–triplet splitting energy (∆*E*_ST_), facilitating the reverse intersystem crossing (RISC) process enabling dark-triplet excitons being endothermally upconverted to the S_1_ state.

**FIGURE 1:**
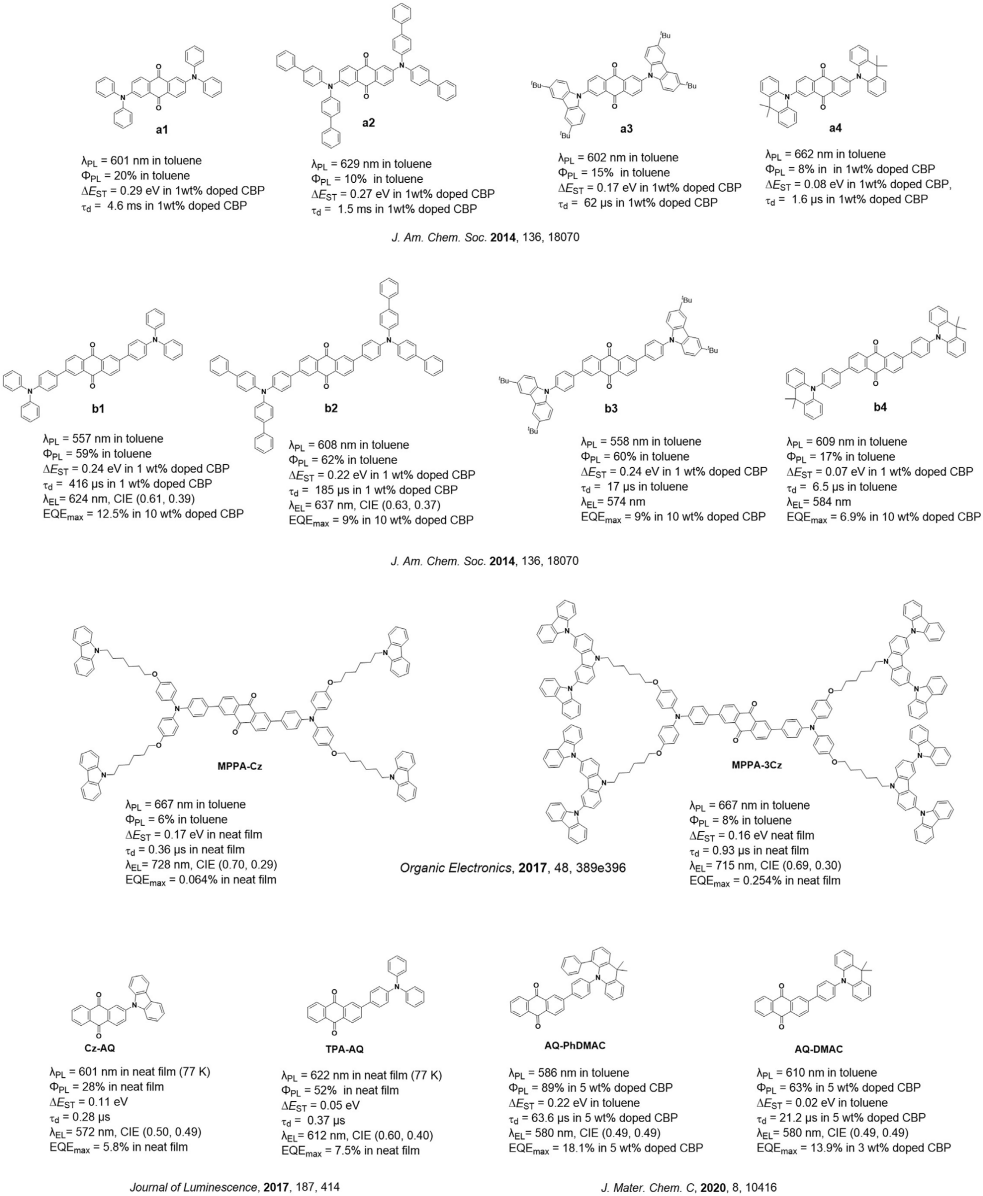
Reported anthraquinone-based TADF emitters.

Currently, significant progress has been made in terms of the design of efficient TADF emitters and OLEDs across the visible spectrum. Owing to the energy gap law, the design of efficient deep-red (DR) or near-infrared (NIR) TADF emitters remains challenging, and there are far fewer examples of these emitters compared to blue and green congeners (Kim et al., 2018). DR and NIR emitters are integral components in medical diagnostics and biomedical imaging (Li et al., 2022), optical communication (Minotto et al., 2020), remote sensing (Zhuo et al., 2022), security (Kim et al., 2014), night vision (Park et al., 2023), and data storage (Xiong et al., 2023). Anthraquinone is a strong acceptor and has been incorporated into a number of low-energy emissive donor–acceptor TADF emitters. In the early work, Adachi and co-workers (Zhang et al., 2014) reported a family of emitters containing an anthraquinone acceptor linked to diphenylamine (**a1–a3)** or triphenylamine donors (**b1–b3**). The family of compounds **b1–b3** has a higher Φ_PL_ value, ranging from 59% to 62%, compared to **a1–a3** (Φ_PL_ = 10%–20%) in toluene. Despite their similarly high Φ_PL_ values, the ∆*E*_ST_ values for **b1**–**b4** (0.22–0.24 eV) are of comparable magnitude to those of **a1**–**a3** (0.17–0.29 eV), and the delayed lifetime (τ_d_) values of the former series span in a wide range from 17 to 416 µs; the τ_d_ value of the latter series ranges from 62 to 4.6 ms. Compounds **b1**–**b4** emit with λ_PL_ values ranging from 557 to 609 nm, while compounds **a1**–**a4** emit with λ_PL_ values ranging from 601 to 662 nm in toluene. Among **b1**–**b4**, the device with compound **b1** showed the best performance, with the maximum external quantum efficiency, EQE_max_, of 12.5% at λ_EL_ of 624 nm (Figure 1).

Sun et al. (2017) reported two near-infrared anthraquinone dendrimers, **MPPA-Cz** and **MPPA-3Cz**, both emitting at 667 nm, have Φ_PL_ values of 6% and 8% and ∆*E*_ST_ values of 0.17 and 0.16 eV in toluene, respectively, while their delayed lifetime (τ_d_) values are 0.36 and 0.93 µs in the neat film. The solution-processed non-doped OLEDs with **MPPA-Cz** and **MPPA-3Cz** showed very low EQE_max_ values of 0.06% and 0.25% at λ_EL_ of 728 and 715 nm, respectively (Figure 1). Previously, Zhang et al. studied D–A–D systems, where the two donors are attached to the anthraquinone acceptor at the 2- and 6-positions, while Bin et al. (2017) reported D–A systems consisting of a carbazole or triphenylamine donor bound to the anthraquinone acceptor at the 2-position. Compounds **Cz-AQ** and **TPA-AQ** have Φ_PL_ values of 28% and 52% and ∆*E*_ST_ values of 0.11 and 0.05 eV in the neat film, respectively. The solution-processed non-doped OLEDs showed EQE_max_ values of 5.8% and 7.5% at λ_EL_ of 572 and 612 nm (Figure 1). Huang (2022) studied the properties of **b1** and its 1,8-disubstituted analog (**1**,**8-2TPA-AQ**) in terms of their chemical structure by single-crystal X-ray diffraction and computationally evaluated their optoelectronic properties at the B3LYP/6-31G(d) level; similarly, the author contrasted **TPA-AQ** (TPA substituted at the 2-position of AQ) and its 1-substituted analog (**1-TPA-AQ**). The calculations revealed that both **1-TPA-AQ** and **1**,**8-2TPA-AQ** have smaller ∆*E*_ST_ values of 0.08 eV and have a low oscillator strength (*f* = S_1_→S_0_) of 0.02 and 0.02 compared to **TPA-AQ** (∆*E*_ST_ = 0.19 eV; *f* = 0.14; S_2_→S_0_) and **b1** (∆*E*_ST_ = 0.21 eV; *f* = 0.33; S_2_→S_0_), respectively. Thus, there appears to be a trade-off between TADF efficiency and Φ_PL_ depending on the substitution pattern of the donors about anthraquinone. Hao et al. (2020) reported the compound **AQ-PhDMAC** that contains a “crooked” conformation of the DMAC donor due to the introduction of a phenyl group at the α-position to the nitrogen of the DMAC donor (PhDMAC = 9,9-dimethyl-4-phenyl-9,10-dihydroacridine). The control emitter **AQ-DMAC** that does not contain the α-phenyl group was also investigated for comparison. **AQ-PhDMAC** and **AQ-DMAC** emit at 586 and 610 nm in toluene, respectively. **AQ-PhDMAC** has a higher Φ_PL_ value of 89% than that of **AQ-DMAC** (Φ_PL_ of 63%) in 5 wt% doped films in CBP but has a much larger ∆*E*_ST_ value of 0.22 eV and longer τ_d_ value of 63.6 µs than those of the control emitter (∆*E*_ST_ = 0.002 eV; τ_d_ **=** 21.2 µs), which is due to the more planar conformation adopted by **AQ-PhDMAC** (torsion angle of 20° between the PhDMAC and phenylene bridge), while the torsion angle between the DMAC and the phenylene bridge increased to 80° in **AQ-DMAC**. Despite the more inefficient TADF, orange-red OLEDs with **AQ-PhDMAC** showed a higher EQE_max_ value of 18.1% at λ_EL_ of 580 nm than those of the devices with **AQ-DMAC** (EQE_max_ = 13.9% at λ_EL_ = 580 nm) in the 5 and 3 wt% doped CBP hosts (Figure 1).

Currently, the reported anthraquinone TADF emitters have their donor groups mostly substituted either at 2- or 2,6-position of anthraquinone. These compounds emit in the orange-red region (λ_PL_ ranging between 557 and 667 nm in toluene) and have τ_d_ values that span widely between 0.28 and 4.6 ms, corresponding to the ∆*E*_ST_ values ranging between 0.02 and 0.29 eV. Amongst the devices utilizing these emitters, the device using **AQ-PhDMAC** showed the maximum efficiency with an EQE_max_ value of 18.1% at a λ_EL_ value of 580 nm. Two near-infrared OLEDs with **MPPA-Cz** and **MPPA-3Cz** were reported, although they showed very low EQE_max_ values. Although the use of anthraquinone as an acceptor has revealed some promising material designs, no research studies have currently investigated its substitution with donors at 1,5- and 1,8-positions and their impact on the optoelectronic properties of these compounds.

**FIGURE 2:**
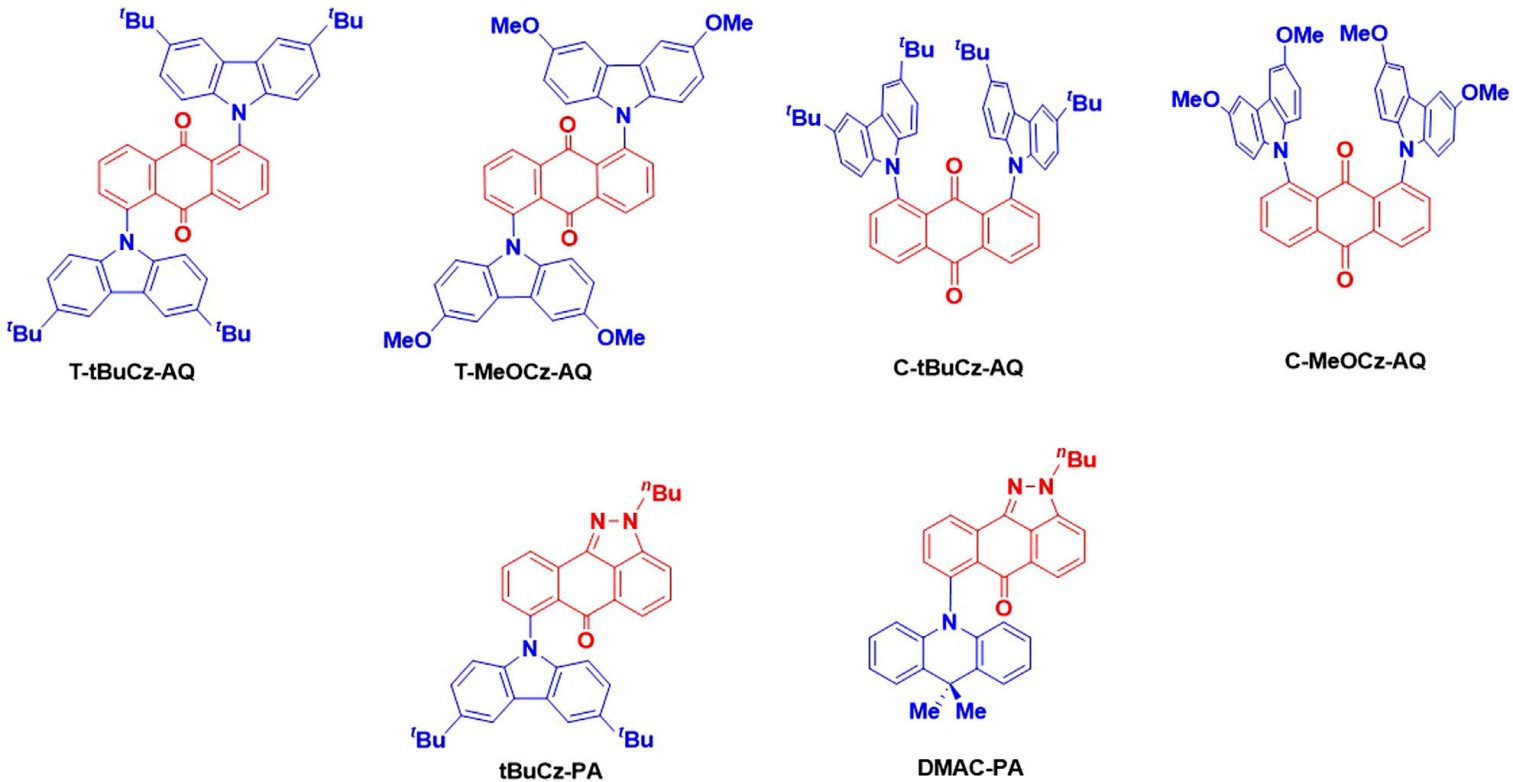
Chemical structures of the targeted emitters.

Herein, we have designed and synthesized a series of emitters based on anthraquinone and related pyrazoloanthrone as acceptor groups that are decorated with either 3,6-di-*tert*-butyl-9*H*-carbazole or 3,6-dimethoxy-9*H*-carbazole and DMAC donors and have investigated their optoelectronic properties (Figure 2). The compounds show a very weak emission ranging from 640 to 750 nm in neat films. The emission of 10 wt% doped films in PMMA as a host matrix similarly ranges from 600 to 700 nm; unfortunately, the photoluminescence quantum yields, Φ_PL_, are very low, ranging from 2.6% to 6.6%. Compounds **T-tBuCz-AQ**, **T-MeOCz-AQ**, and **C-tBuCz-AQ** showed TADF with short delay lifetimes (τ_d_) of 1 µs, 132 ns, and 0.7 µs, respectively. We also observed that **tBuCz-PA** showed reversible photoluminescence (PL) switching upon mechanical grinding and hexane solvent fuming.

## Results and discussion

### Synthesis and characterization

**SCHEME 1:**
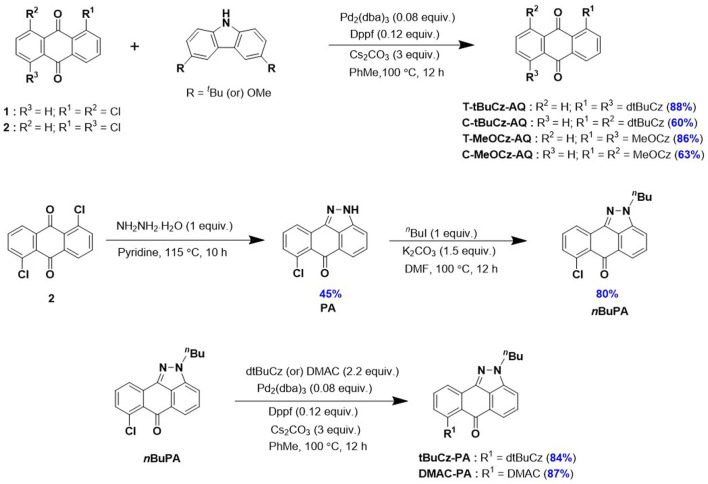
Synthesis of targeted emitters.

The syntheses of the targeted emitters are shown in Scheme 1. Anthraquinone acceptor-based emitters **T-tBuCz-AQ**, **C-tBuCz-AQ**, **T-MeOCz-AQ**, and **C-MeOCz-AQ** were obtained following a Buchwald–Hartwig cross-coupling reaction of 1,5-dichloroanthraquinone (**1**) or 1,8-dichloroanthraquinone (**2**) with 3,6-di-*tert*-butylcarbazole (dtBuCz) or 3,6-dimethoxy-9*H*-carbazole (dMeOCz) in good yields (63%–93%). The intermediate *n*BuPA was prepared following the reaction of **2** with hydrazine monohydrate to furnish **PA**, which was then butylated in an overall yield of 36% (Ganduri et al., 2018). Pyrazoloanthrone-based emitters **tBuCz-PA** and **DMAC-PA** were also synthesized following the same cross-coupling strategy in 84% and 87% yields, respectively. The compounds were characterized by NMR (^1^H and ^13^C) spectroscopy, high-resolution mass spectrometry (HRMS), high-performance liquid chromatography (HPLC), melting point determination, and X-ray diffraction analysis (Supplementary Figures S1–S24).

**FIGURE 3:**
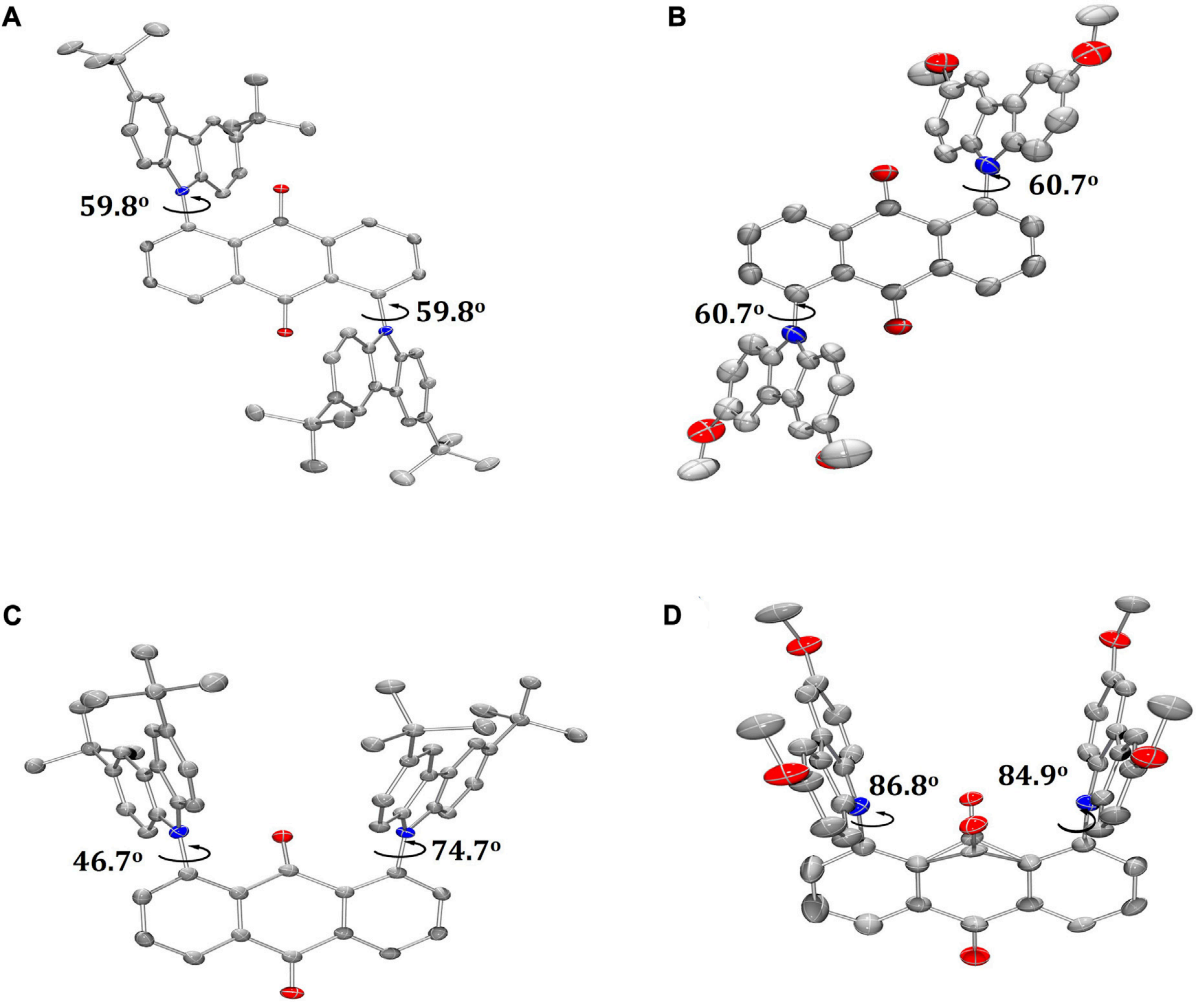
ORTEP molecular structures of **(A) T-tBuCz-AQ**, **(B) T-MeOCz-AQ**, **(C) C-tBuCz-AQ**, and **(D) C-MeOCz-AQ** (hydrogen atoms are omitted for clarity; displacement parameters are drawn at the 50% probability level).

Single crystals of **T-tBuCz-AQ**, **T-MeOCz-AQ**, and **C-tBuCz-AQ** were obtained directly via temperature-gradient vacuum sublimation (Figure 3), while the crystals of **C-MeOCz-AQ** were obtained via isolation from a mixture of EtOAc and DCM. **T-tBuCz-AQ**, **T-MeOCz-AQ**, **C-tBuCz-AQ**, and **C-MeOCz-AQ** crystallized in monoclinic, triclinic, tetragonal, and rhombohedral crystal systems with the corresponding space groups of *P*2_1_/c, *P*-1, *P*43, and *R*-3, respectively. Each tBuCz has the same dihedral angle of 59.8° with respect to the anthraquinone acceptor in **T-tBuCz-AQ**, and the angle is effectively the same at 60.7° in **T-MeOCz-AQ**. On the other hand, for **C-tBuCz-AQ**, each tBuCz adopts one of the two dihedral angles of 46.7° and 74.7° with anthraquinone. In the case of **C-MeOCz-AQ**, nearly the same dihedral angles (86.8° and 84.9°) were observed between MeOCz and the anthraquinone acceptor.

### DFT calculations

**FIGURE 4:**
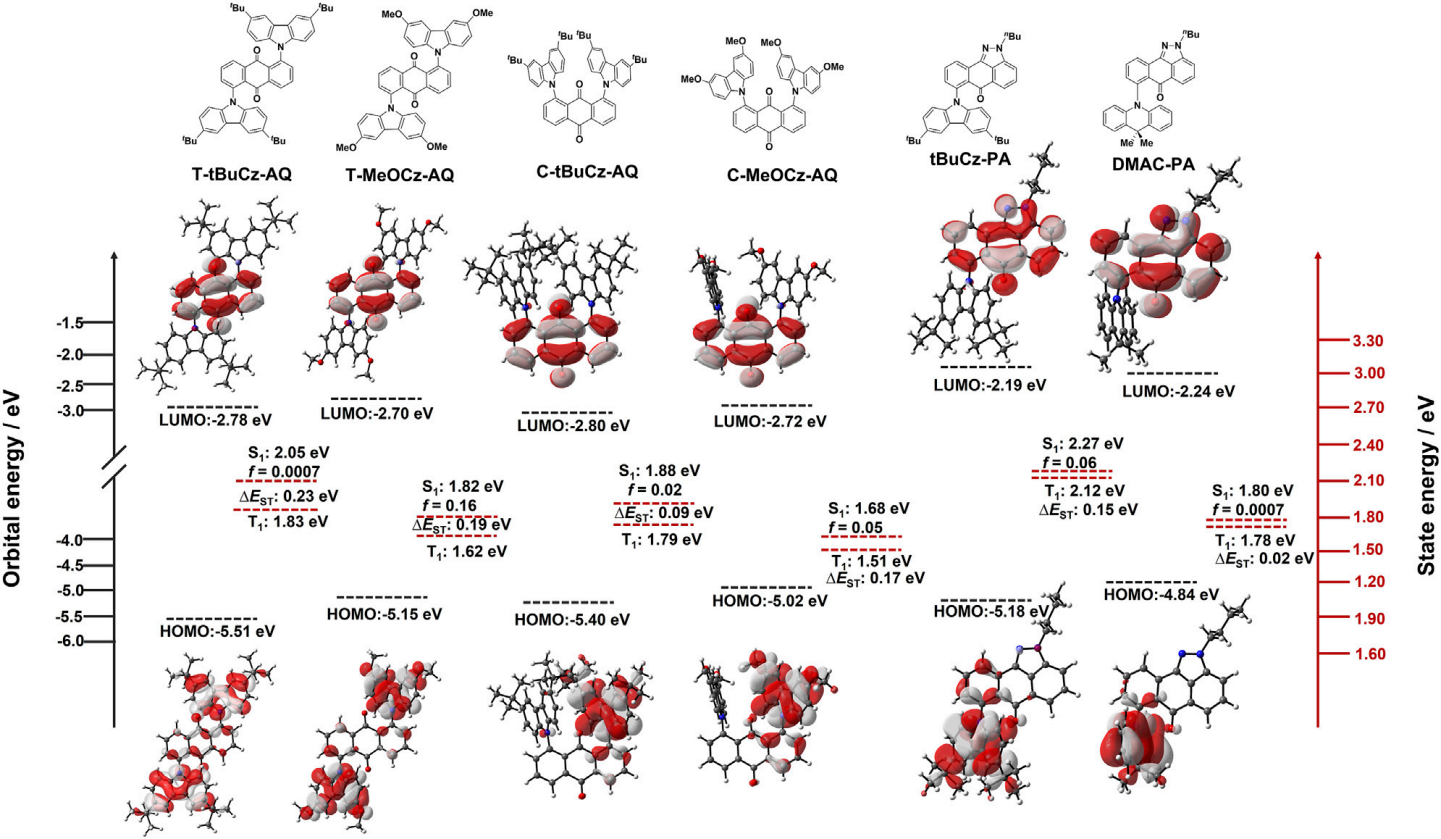
DFT modeling of energies and electron density distributions of the HOMO/LUMO (ISO value = 0.02) and their associated vertical excitation energies in S_1_ and T_1_ states of **T-tBuCz-AQ**, **T-MeOCz-AQ**, **C-tBuCz-AQ**, **C-MeOCz-AQ**, **tBuCz-PA**, and **DMAC-PA**.

Density functional theory (DFT) calculations at the PBE0/6-31G(d,p) level of theory in the gas phase were performed in order to gain a deeper understanding of the electronic structures of all compounds (Figure 4). For **T-tBuCz-AQ**, the highest occupied molecular orbital (HOMO) is localized on the carbazole donors, with a minor contribution from anthraquinone, while the lowest unoccupied molecular orbital (LUMO) is localized on anthraquinone. Similar HOMO and LUMO distributions are noted for **T-MeOCz-AQ**. As expected, the HOMO level for **T-MeOCz-AQ** is slightly destabilized compared to that of **T-tBuCz-AQ** due to the stronger electron-donating character of the dMeOCz donor compared to dtBuCzs, while the LUMO levels are also destabilized, although less so than the HOMO levels, reflecting the poor but non-trivial electronic coupling of donors with the AQ acceptor. In the case of **C-tBuCz-AQ** and **C-MeOCz-AQ**, HOMOs and LUMOs are exclusively localized on the carbazole donors and anthraquinone, respectively, and no overlap is observed. A consequence of the near-zero orbital overlap is that although, as expected, the HOMO of **C-MeOCz-AQ** is destabilized compared to that of **C-tBuCz-AQ**, the LUMO levels are effectively the same in these two compounds. In the case of **tBuCz-PA**, the HOMO is delocalized on the carbazole donors and pyrazoloanthrone, while the LUMO is located on pyrazoloanthrone. Due to the orthogonal conformation of DMAC in **DMAC-PA**, the HOMO is mostly localized on DMAC, while the LUMO is located on the pyrazoloanthrone ring. As expected, the HOMO levels are correlated with the electron-donating strength of the donor group, while LUMO levels are similar; the more destabilized LUMO in **tBuCz-PA** is a reflection of the greater electronic coupling with the donor in this compound. As expected, the calculated optical gaps of 2.73 eV for **T-tBuCz-AQ** and 2.60 eV for **C-tBuCz-AQ** decrease to 2.45 and 2.30 eV for **T-MeOCz-AQ** and **C-MeOCz-AQ**, respectively. Similarly, **DMAC-PA** has a smaller optical gap of 2.60 eV than **tBuCz-PA** (2.99 eV).

The excited-state properties were calculated using time-dependent density functional theory (TD-DFT) within the Tamm−Dancoff approximation (TDA-DFT) based on optimized ground-state geometries (Hirata and Head-Gordon, 1999). The S_1_/T_1_ energy levels are 2.05/1.82 eV for **T-tBuCz-AQ**, which decreases to 1.81/1.62 eV for **T-MeOCz-AQ**. These energy levels for **C-tBuCz-AQ** and **C-MeOCz-AQ** are further stabilized to 1.88/1.79 eV and 1.68/1.51 eV, respectively. However, in the case of **tBuCz-PA** and **DMAC-PA**, the S_1_/T_1_ energy levels are found to be 2.27/2.11 eV and 1.79/1.77 eV, respectively (Table 1). The calculated ∆*E*_ST_ values are in the range of 0.19–0.23 eV. Natural transition orbital (NTO) analysis of **T-tBuCz-AQ** reveals that both S_1_- and T_1_-state particles are located on tBuCz donors, while the holes are localized on anthraquinone, illustrating its charge transfer transition (CT) character (Supplementary Table S1). Similarly, NTO distributions were noted for the S_1_ and T_1_ states of **T-MeOCz-AQ**, while for **C-tBuCz-AQ** and **C-MeOCz-AQ**, the hole is located on one of the two Cz donors and the particle is localized on anthraquinone, indicating the CT character of these states. Similar hole and particle localizations on the donor (Cz/DMAC) and anthraquinone were noted for **tBuCz-PA** and **DMAC-PA**. The spin–orbit coupling matrix elements (SOCMEs) between S_1_ and T_1_ at the optimized S_1_ geometry of **T-tBuCz-AQ** (<S_1_|*Ĥ*_SOC_|T_1_> = 0.07 cm^−1^) and **C-tBuCz-AQ** (<S_1_|*Ĥ*_SOC_|T_1_> = 0.06 cm^−1^) are larger than those of **T-MeOCz-AQ** (<S_1_|*Ĥ*_SOC_|T_1_> = 0.02 cm^−1^) and **C-MeOCz-AQ** (<S_1_|*Ĥ*_SOC_|T_1_> = 0.001 cm^−1^), despite having similar NTOs for all four compounds; a similar trend in SOCME values between S_1_ and T_2_ was also observed (Supplementary Table S2). For **tBuCz-PA** and **DMAC-PA**, the hole and particle are localized on the donor (tBuCz/DMAC) and pyrazoloanthrone, respectively, in both S_1_ and T_1_ states, implying their CT character. However, since both the hole and particle in T_2_ are localized on pyrazoloanthrone, this state possesses the LE character. As a result, the SOCME values between S_1_ and T_2_ are much larger than those between S_1_ and T_1_ for **tBuCz-PA** and **DMAC-PA** (Supplementary Table S2).

**TABLE 1:** Electrochemical data.

Emitter	E_ox_/V^a^	E_red_/V^a^	HOMO/eV^b^	LUMO/eV^b^	∆*E*_HL_/eV^c^
**T-tBuCz-AQ**	1.25	−0.88	−5.59	−3.46	2.13
**T-MeOCz-AQ**	1.06	−0.85	−5.40	−3.49	1.91
**C-tBuCz-AQ**	1.23	−0.94	−5.57	−3.40	2.17
**C-MeOCz-AQ**	0.97	−0.92	−5.31	−3.42	1.89
**tBuCz-PA**	1.15	−1.31	−5.49	−3.03	2.46
**DMAC-PA**	0.69	−1.30	−5.03	−3.04	1.99

^a^ Obtained from the DPV peaks and referenced with respect to SCE (Fc/Fc^+^ = 0.46 V for DCM) (Connelly and Geiger, 1996).

^b^ E_HOMO/LUMO_ = −[E^ox/red^(*vs.* Fc/Fc^+^) + 4.8] eV.

^c^ ∆*E*_HL_ = |*E*_HOMO_−*E*_LUMO_| (Cardona et al., 2011).

### Cyclic voltammetry

**FIGURE 5:**
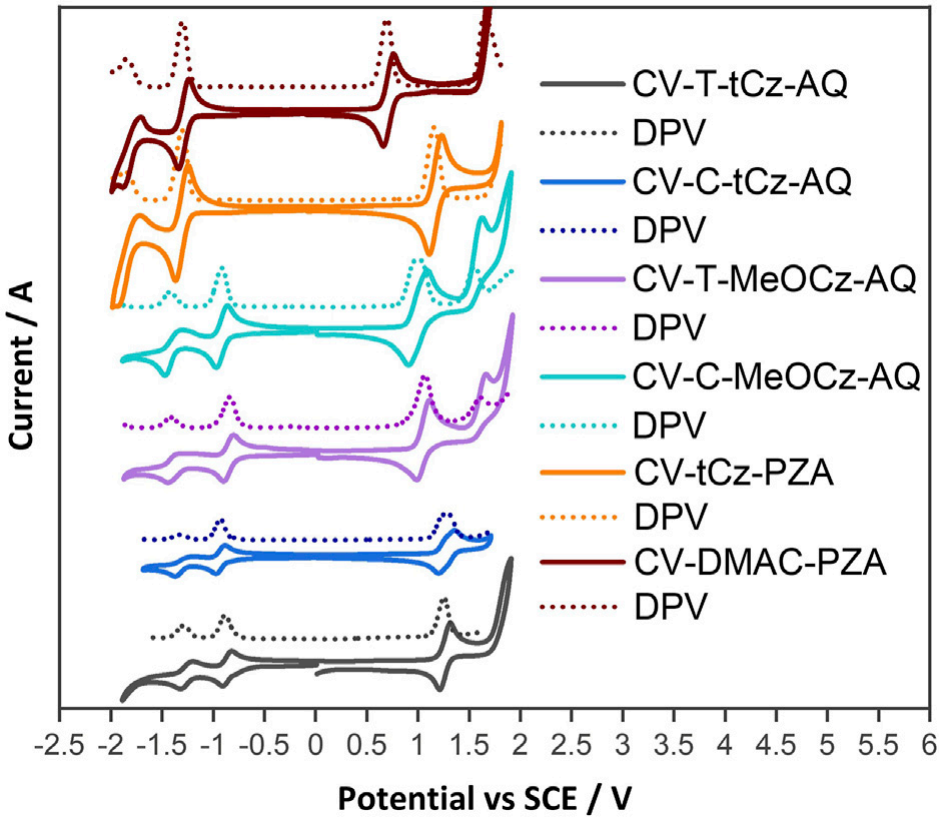
Cyclic voltammograms (CVs) and differential pulse voltammograms (DPVs) of compounds in the N_2_-saturated DCM solution with 0.1 M [^*n*^Bu_4_N]PF_6_ as the supporting electrolyte and Fc/Fc^+^ as the internal reference (0.46 V for DCM vs. SCE) at a scan rate of 100 mV·s^−1^.

HOMO and LUMO energies were estimated from the oxidation and reduction potentials measured using cyclic voltammetry (CV) and differential pulse voltammetry (DPV) in dichloromethane at a scan rate of 100 mV·s^−1^ with tetra-*n*-butylammonium hexafluorophosphate as the supporting electrolyte (Figure 5). The values are reported against a standard calomel electrode (SCE). Two reversible reduction waves were observed for the anthraquinone acceptor-based compounds. The first reduction potentials are −0.88, −0.85, −0.94, and −0.92 V for **T-tBuCz-AQ**, **T-MeOCz-AQ**, **C-tBuCz-AQ**, and **C-MeOCz-AQ**, respectively (Table 1). The values are nearly the same for each of the *trans*-substituted derivatives (**T-tBuCz-AQ** and **T-MeOCz-AQ)** and for *cis*-substituted derivatives (**C-tBuCz-AQ** and **C-MeOCz-AQ)**. The corresponding LUMO values of **T-tBuCz-AQ**, **T-MeOCz-AQ**, **C-tBuCz-AQ**, and **C-MeOCz-AQ** are −3.46, −3.49, −3.40, and −3.42 eV. Although the DFT-predicted LUMO levels are stabilized at −2.78, −2.70, −2.80, and −2.72 eV, the trend in the values imply an insensitivity of the LUMO energy to the relative positions of the donors or the strength of the donors.

The first oxidation waves were all found to be reversible, with potentials, *E*_ox_, of 1.25, 1.06, 1.23, and 0.97 V for **T-tBuCz-AQ**, **T-MeOCz-AQ**, **C-tBuCz-AQ**, and **C-MeOCz-AQ**, respectively. The *E*_ox_ values of the two compounds with dMeOCz donors (**T-MeOCz-AQ** and **C-tBuCz-AQ)** are cathodically shifted compared to those with dtBuCz donors (**T-tBuCz-AQ** and **T-MeOCz-AQ)**, reflective of their stronger electron-donating ability. The corresponding HOMO levels of **T-tBuCz-AQ**, **T-MeOCz-AQ**, **C-tBuCz-AQ**, and **C-MeOCz-AQ** are −5.59, −5.40, −5.57, and −5.31 eV, which align with the trend of the DFT-predicted HOMO levels of −5.51, −5.15, −5.40, and −5.02 eV, respectively. Consequently, the experimentally observed electrochemical bandgap, ∆*E*, for **T-MeOCz-AQ** is 1.91 eV and for **C-MeOCz-AQ** is 1.89 eV, while those of **T-tBuCz-AQ** and **C-tBuCz-AQ** are expectedly wider at 2.13 and 2.17 eV, respectively (Table 1).

Both **DMAC-PA** and **tBuCz-PA** show reversible reduction and oxidation waves via CV. Their respective reduction potentials are −1.31 and −1.30 V, indicating that the electronic structure of the acceptor core is not affected by different donor attachments. These reduction potentials are cathodically shifted compared to anthraquinone derivatives, implying that the pyrazoloanthrone acceptor is weaker than anthraquinone. The *E*_ox_ value of **DMAC-PA** at 0.69 V is cathodically shifted compared to that of **tBuCz-PA** (1.15 V) due to the use of the stronger DMAC donor in the former. The **∆** *E* value of **DMAC-PA** is 1.99 eV, which is much smaller than that of **tBuCz-PA** (2.46 eV). These trends match well with HOMO–LUMO gaps predicted by DFT.

### Photophysical properties

**FIGURE 6:**
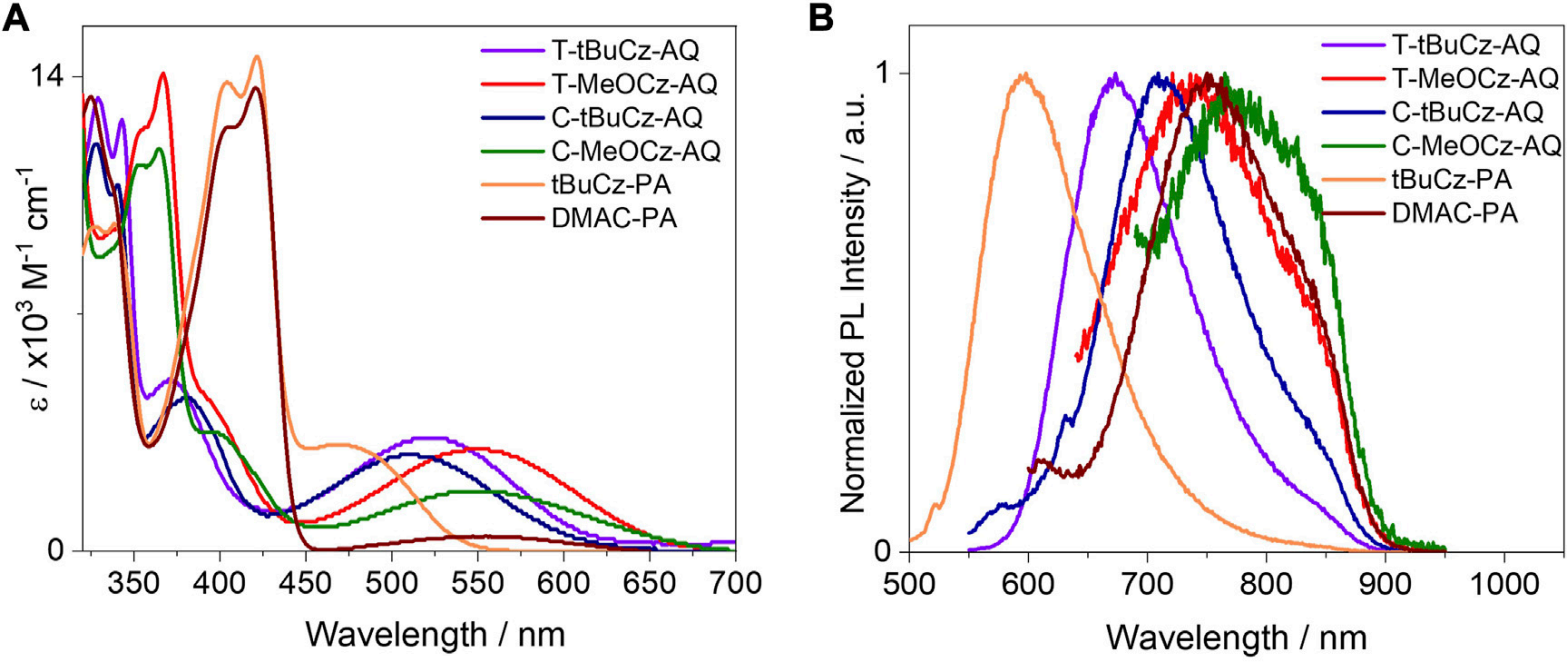
**(A)** UV-vis absorption and **(B)** PL at room temperature in toluene (λ_exc_ = 450 nm).

The absorption spectra recorded in toluene are shown in Figure 6. These compounds exhibit a broad absorption band in the region of 450–700 nm and high-energy structured absorption bands in the region of 320–450 nm. The broad low-energy bands are assigned to charge transfer transitions from the donors to anthraquinone/pyrazoloanthrone acceptors. Both **T-MeOCz-AQ** and **C-MeOCz-AQ** showed a red-shifted absorption maximum at 550 nm compared to the CT bands of **T-tBuCz-AQ** (525 nm) and **C-tBuCz-AQ** (516 nm), which aligns with their smaller electrochemical gaps. The higher energy absorption bands of approximately ∼328 and 340 nm for **T-tBuCz-AQ** and **C-tBuCz-AQ** are assigned to π → π* transitions of the tBuCz donor as they match the absorption spectrum of tBuCz itself (Supplementary Figure S25). Similarly, the absorption bands of approximately ∼354 and ∼365 nm are assigned to the MeOCz-centered π → π* transitions (Supplementary Figure S25). The molar extinction coefficients, *ε*, of the CT band of **T-tBuCz-AQ** (3.3 × 10^3^ M^−1^·cm^−1^) and **T-MeOCz-AQ** (3.0 × 10^3^ M^−1^·cm^−1^) are slightly larger than those of **C-tBuCz-AQ** (2.8 × 10^3^ M^−1^·cm^−1^) and **T-MeOCz-AQ** (1.8 × 10^3^ M^−1^·cm^−1^). Similarly, as expected, **DMAC-PA** showed a red-shifted absorption band at 555 nm compared to **tBuCz-PA** (470 nm). The CT band in **tBuCz-PA** (*ε* = 3.1 × 10^3^ M^−1^·cm^−1^) is more intense than that in **DMAC-PA** (4.1 × 10^2^ M^−1^·cm^−1^), reflective of the greater electronic coupling between the donor and the PA acceptor in the former. The absorption bands of approximately ∼403 and ∼422 nm are assigned to LE transitions of the PA acceptor as these bands match those of the acceptor (Supplementary Figure S25).

**FIGURE 7:**
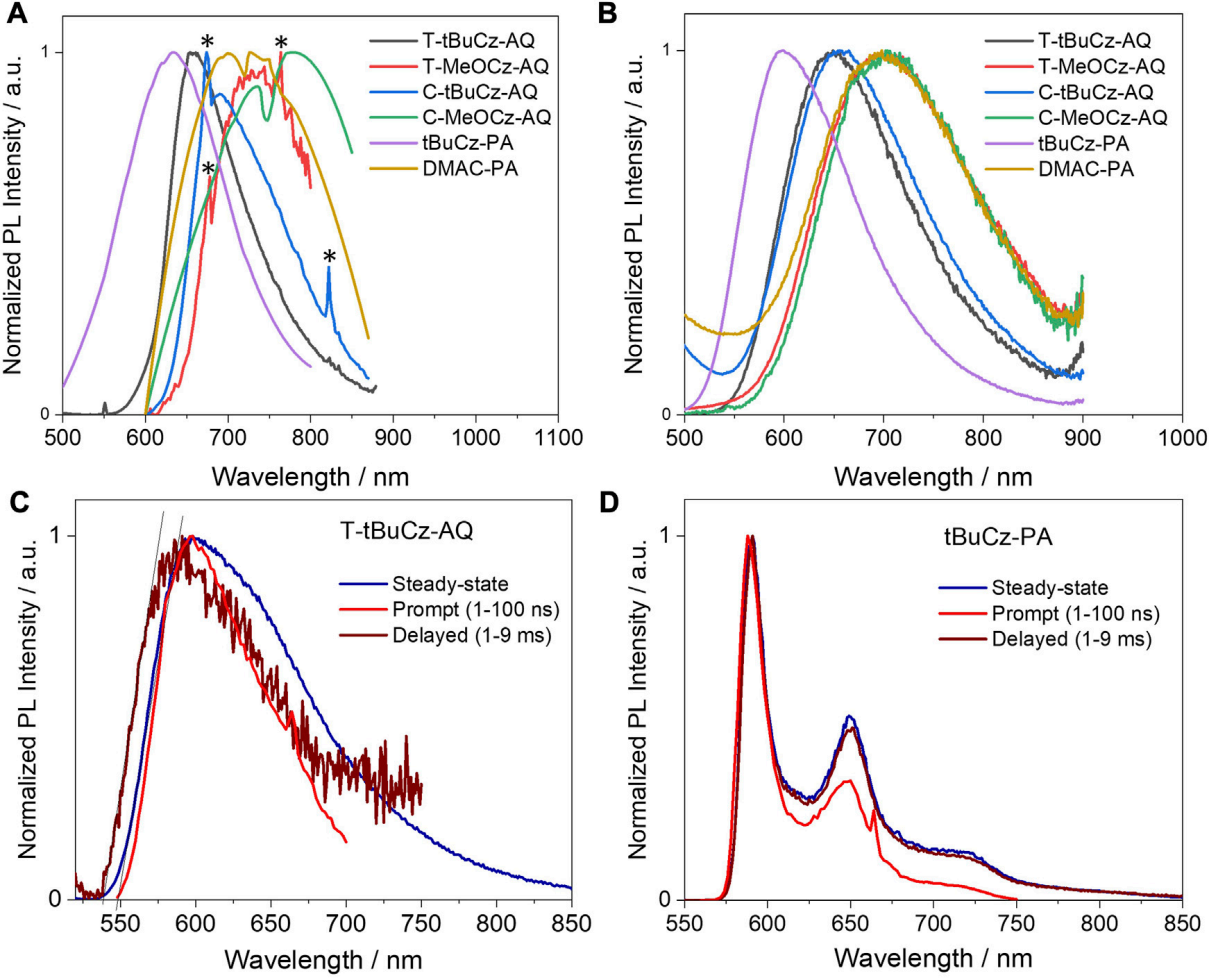
Steady-state PL (λ_exc_ = 450 nm) spectra of **(A)** powders (labeled peaks with “*” are noisy due to poorly emissive samples) and **(B)** 10 wt% doped films in PMMA (λ_exc_ = 450 nm); prompt and delayed emission spectra in 2-MeTHF glass at 77 K (λ_exc_ = 450 nm) of **(C) T-tBuCz-AQ** and **(D) T-tBuCz-PA**.

The compounds showed broad and weak emissions in toluene solutions. Compounds **T-MeOCz-AQ** (λ_PL_ = 735 nm) and **C-MeOCz-AQ** (λ_PL_ = 770 nm) showed red-shifted emission compared to their *tert*-butylcarbazole containing counterparts **T-tBuCz-AQ** (λ_PL_ = 670 nm) and **C-tBuCz-AQ** (λ_PL_ = 710 nm). Compound **T-tBuCz-AQ** exhibited a longer lifetime of 16.4 ns than **C-tBuCz-AQ** (τ_PL_ = 8.0 ns), while no significant difference in lifetimes was observed for **T-MeOCz-AQ** (τ_PL_ = 7.7 ns) and **C-MeOCz-AQ** (τ_PL_ = 6.5 ns) (Supplementary Figure S26). The compound **DMAC-PA** (λ_PL_ = 750 nm) showed significantly red-shifted emission compared to **tBuCz-PA** (λ_PL_ = 594 nm). The lifetime of **tBuCz-PA** is 34 ns, while no emission decay could be detected in the temperature-dependent time-resolved PL (TRPL) for **DMAC-PA**. The Φ_PL_ value could not be determined as the emission was too weak in the solution. The PL spectra for the powders are shown in Figure 7A. The compound **T-tBuCz-AQ** (τ_PL_ = 23 ns) showed structure-less emissions at 656 nm, while a slightly red-shifted emission at 684 nm was observed for **C-tBuCz-AQ** (τ_PL_ = 25 ns). Similar trends were observed for **T-MeOCz-AQ** (λ_PL_ = 728 nm; no decay observed) and **C-MeOCz-AQ** (λ_PL_ = 752 nm; τ_PL_ = 14 ns). Similar to that observed in toluene, the emission of **DMAC-PA** (λ_PL_ = 730 nm; τ_PL_ = 30 ns) is significantly red-shifted compared to that of **tBuCz-PA** (λ_PL_ = 637 nm; τ_PL_ = 16 ns) (Supplementary Figure S27).

Next, we investigated the PL properties of these compounds in 10 wt% doped films in PMMA. Compounds **T-tBuCz-AQ**, **T-MeOCz-AQ**, **C-tBuCz-AQ**, and **C-MeOCz-AQ** showed structure-less emissions at λ_PL_ of 650, 700, 655, and 700 nm, respectively, illustrating again that the emission of the *cis*-isomers is red-shifted compared to that of their *trans*-counterparts; aligning with previous measurements in the solution and powder, the emission of **DMAC-PA** at 700 nm is red-shifted compared to that of **tBuCz-PA** at 600 nm (Figure 7B). The corresponding Φ_PL_ values of the emitters are low (Table 2), in part due to the energy gap law, at 6.6/3.9, 2.7/2.4, 3.6/3.6, 3.0/2.6, 2.8/2.8, and 2.6/2.6 in N_2_/air.

**TABLE 2:** Summary of the photophysical properties of the 10 wt% doped films in PMMA.

Compound	λ_PL_/nm	Φ_PL_ (O_2_/N_2_)/%^a^	τ_p_/ns^b^	τ_d_/µs^c^
**T-tBuCz-AQ**	650	3.9/6.6	3.5	1.00
**T-MeOCz-AQ**	700	2.4/2.7	14	0.132
**C-tBuCz-AQ**	655	3.6/3.6	88	0.704
**C-MeOCz-AQ**	700	2.6/3.0	2	—
**tBuCz-PA**	600	2.8/2.8	5.2	335
**DMAC-PA**	700	2.6/2.6	21	—

^a^ Φ_PL_ was recorded under air/N_2_ atmosphere using an integrating sphere (λ_exc_ = 450 nm).

^b^ Prompt lifetime (τ_p_) was recorded using time-correlated single-photon counting (TCSPC) (λ_exc_ = 375 nm).

^c^ Delayed lifetime (τ_d_) was recorded using TCSPC for **T-tBuCz-AQ**, **T-MeOCz-AQ**, and **C-tBuCz-AQ** and a microsecond flash lamp for **tBuCz-PA** (λ_exc_ = 450 nm).

To determine the Δ*E*_ST_ value in these compounds, we measured the steady state, prompt, and delayed emission spectra in 2-MeTHF glass at 77 K (Figures 7C, D). **T-tBuCz-AQ** showed a prompt emission at 597 nm, while the delayed emission (gated time = 1 ms) peaks at 594 nm. S_1_ and T_1_ energies, determined from the onset of prompt and delayed emissions, are 2.26 and 2.28 eV, respectively, and ∆*E*_ST_ is −20 meV. This apparent small inverted gap must have resulted from the prompt and delayed emissions, occurring from different conformer species in the glass matrix; furthermore, it is possible that 77 K is not sufficiently cold to deconvolute the phosphorescence spectrum from the residual delayed emission. Compounds **T-MeOCz-AQ**, **C-tBuCz-AQ**, and **C-MeOCz-AQ** showed steady-state emission at 640, 635, and 680 nm, respectively, yet no delayed emission was detected, likely due to the too weak emission of these compounds. The corresponding broad and structure-less emission stems from the CT nature of the S_1_ state (Supplementary Figure S28). In contrast, **tBuCz-PA** showed a structured prompt emission peaking at 590 nm, which is also same as the emission observed in the time-gated PL (Figure 7D), meaning that the emission only occurs from the anthrone acceptor moiety (Supplementary Figure S29). Compound **DMAC-PA** showed very weak steady-state emission, while no delayed emission was detected.

**FIGURE 8:**
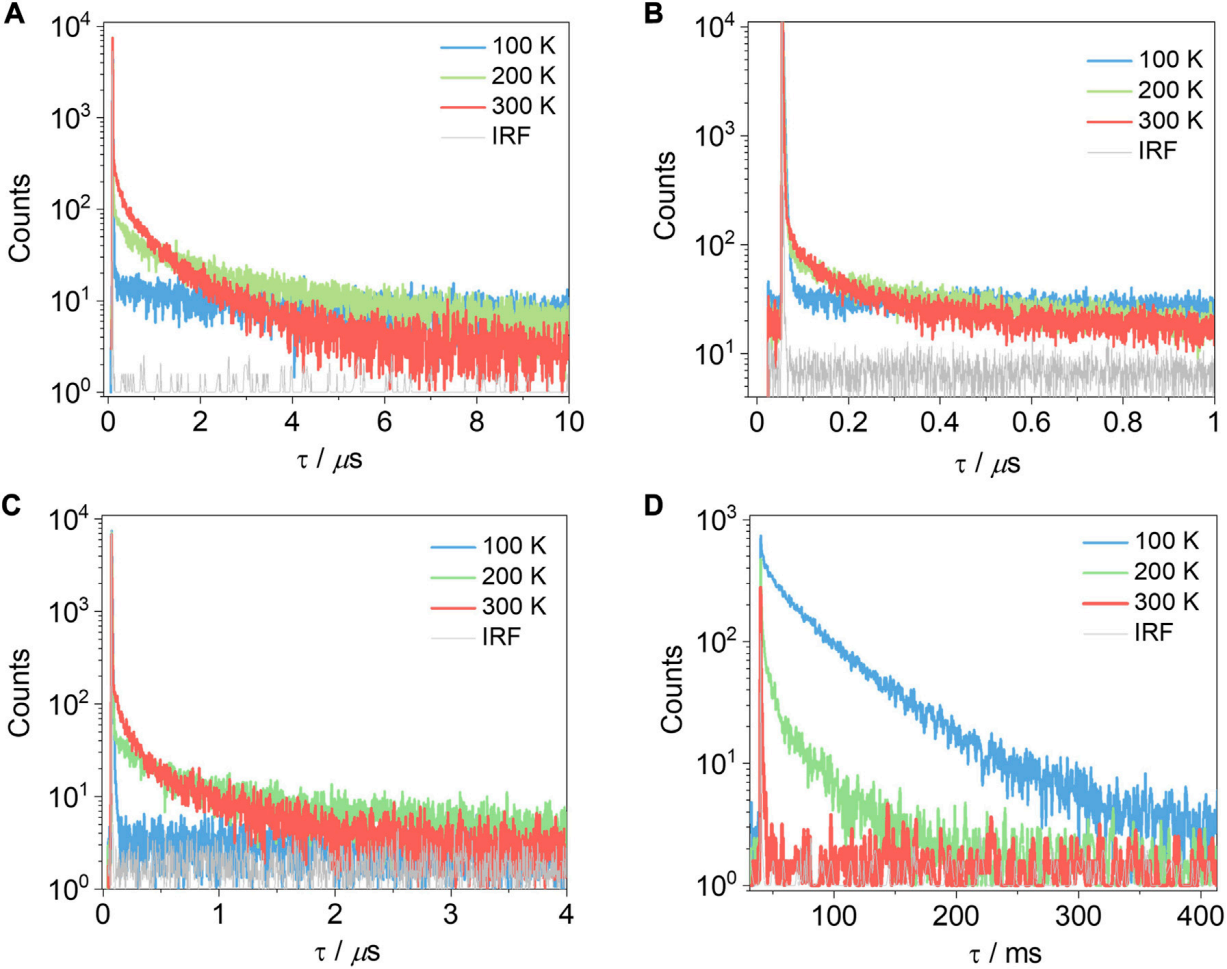
Time-resolved photoluminescence decays of **(A) T-tBuCz-AQ**, **(B) T-MeOCz-AQ**, **(C) C-tBuCz-AQ**, and **(D) tBuCz-PA** in 10 wt% doped films in PMMA (λ_exc_ = 450 nm).

Despite the rather inconclusive results from 2-MeTHF glass measurements to ascertain the Δ*E*_ST_ values, we next measured the temperature-dependent time-resolved PL decays of the compounds in 10 wt% doped films in PMMA (Figure 8). Compounds **T-tBuCz-AQ**, **T-MeOCz-AQ**, and **C-tBuCz-AQ** all showed increasing delayed emission with the increasing temperature, indicative that these compounds exhibit TADF behavior. The prompt and delayed lifetimes (τ_p_ and τ_d_) of **T-tBuCz-AQ** are 3.5 ns and 1 µs at room temperature, while those of **T-MeOCz-AQ** and **C-tBuCz-AQ** are 14.0 and 88.0 ns and 132 and 704 ns, respectively. The rate constants for ISC and RISC are reported in Supplementary Table S3 (Tsuchiya et al., 2021). In contrast, the TRPL of **tBuCz-PA** showed a behavior where non-radiative decay is dominant at elevated temperatures and there is no evidence of TADF.

**FIGURE 9:**
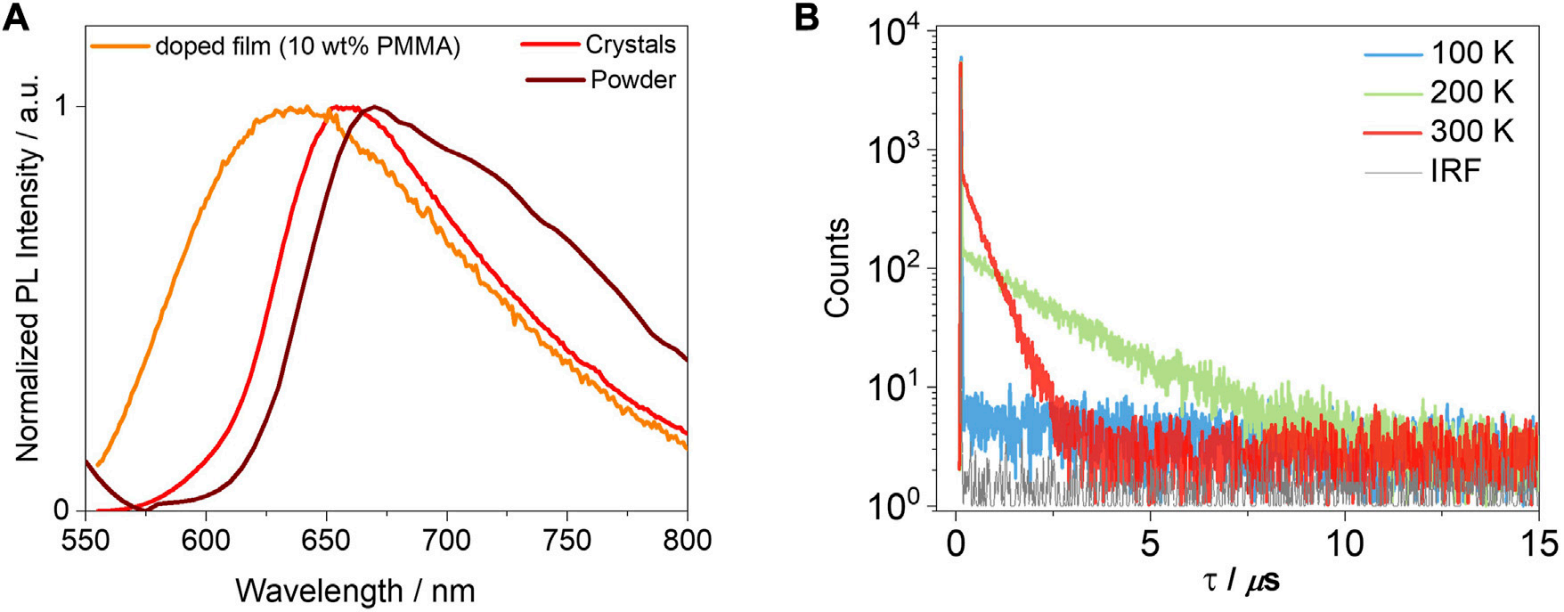
**(A)** PL spectra of crystals and 10 wt% doped film in PMMA (λ_exc_ = 450 nm); **(B)** time-resolved PL decays of crystals of **T-tBuCz-AQ** (λ_exc_ = 375 nm).

Next, we investigated the photophysical properties of the crystals. The crystals of **T-tBuCz-AQ** emit at λ_PL_ of 658 nm and have a Φ_PL_ value of 5%, while the crystals of the other compounds in this study are not luminescent. The emission of the crystals of **T-tBuCz-AQ** is red-shifted compared to the 10 wt% doped film in PMMA (λ_PL_ = 640 nm) and blue-shifted with respect to the powder (λ_PL_ = 670 nm) (Figure 9A). The crystals also showed a delayed emission, with a τ_d_ value of 0.5 µs. The TADF behavior was confirmed by variable temperature time-resolved PL measurements (Figure 9B).

### Mechanochromism

**FIGURE 10:**
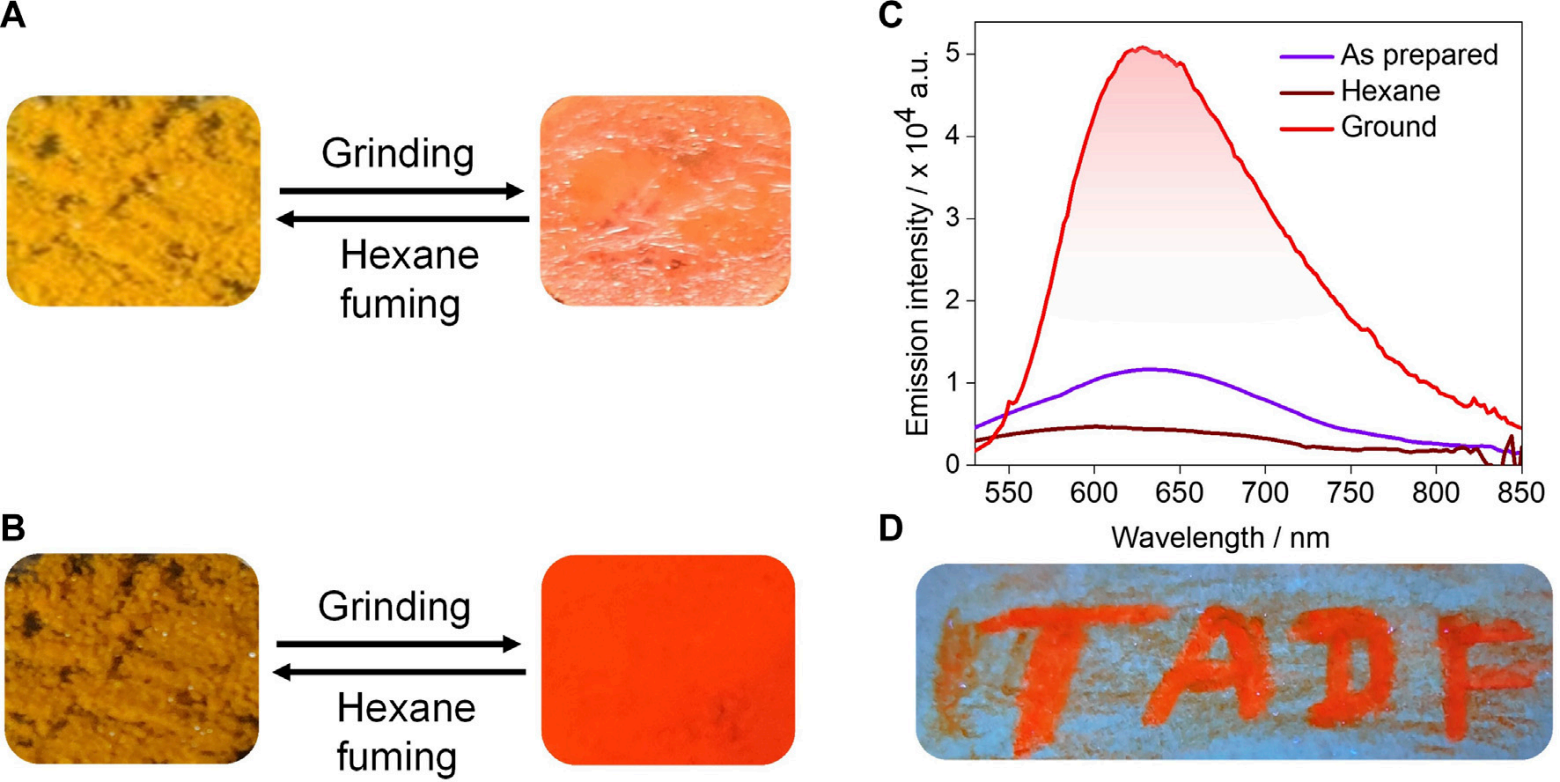
Reversible switching of luminescence of **tBuCz-PA** upon grinding and hexane fuming: **(A)** under daylight, **(B)** under UV light, **(C)** corresponding PL spectra, and **(D)** filter paper coated with **tBuCz-PA** showing that the written text “TADF” is orange luminescent, while the remaining portion is non-luminescent.

Among all the compounds, **tBuCz-PA** showed a distinct PL response with mechanical pressure (Figures 10A, B). As-prepared **tBuCz-PA** showed a very weak emission at 640 nm. When mechanical pressure was applied to the as-prepared sample of **tBuCz-PA**, an enhancement of the PL intensity with a slightly blue-shifted emission (630 nm) was observed, which is linked to an increase in the Φ_PL_ value ranging from 1.2% to 5.3% (Figure 10C). The ground form was reverted to the original compound upon exposure to hexane vapors. The reversibility of this process was confirmed by repeated cycles of grinding and fuming experiments (Supplementary Figure S30). The powder X-ray diffraction (PXRD) of the as-prepared compound exhibits reflection peaks, indicating its crystalline nature, while the ground form did not show any significant reflection peaks, pointing to its amorphous state (Supplementary Figure S31). Thus, the change in luminescence upon grinding is due to a crystalline-to-amorphous transition. High-contrast mechanochromism was exploited in an invisible ink application, where a filter paper was coated with **tBuCz-PA** and only when pressure was applied, the text “TADF” appeared upon excitation using a UV torch, as shown in Figure 10D.

## Conclusion

Herein, the synthesis, single-crystal X-ray diffraction, and optoelectronic characterization of donor–acceptor anthraquinone- and pyrazoloanthrone-based emitters are discussed. The anthraquinone-based compounds in this study show a red-shifted emission in toluene (λ_PL_ value ranging between 670 and 770 nm) compared to the literature-reported anthraquinone TADF emitters; this is due to the differing regiochemistry of the donors. The compounds emit in the spectral range 600–700 nm in 10 wt% doped films in PMMA and 640–750 nm in the neat films. Of the compounds investigated, **T-tBuCz-AQ**, **T-MeOCz-AQ**, and **C-tBuCz-AQ** showed TADF behavior in 10 wt% doped films in PMMA, while the crystals of **T-tBuCz-AQ** also showed TADF behavior. The related pyrazoloanthrone-based emitters showed a blue-shifted emission, compared to D–A anthraquinone-based emitters with the same donor, and did not show any TADF behavior. Of the compounds studied, only **tBuCz-PA** was found to exhibit high-contrast reversible mechanochromism.

## Data Availability

The datasets presented in this study can be found in online repositories. The names of the repository/repositories and accession number(s) can be found in the article/Supplementary Material. The research data supporting this publication can be accessed at https://doi.org/10.17630/84d1a524-3e28-4c7d-9fd7-9e038ceffaf3.
