# Supplementary material for: Thermally activated delayed fluorescence and high-contrast mechanochromism of anthrone-based donor–acceptor systems
Source: Front Chem. 2023 Aug 31;11:1248267. doi: 10.3389/fchem.2023.1248267 (PMC10501131; doi:10.3389/fchem.2023.1248267)
Supplement: Supplementary file 1 [file DataSheet2.pdf]

Thermally Activated Delayed Fluorescence and High-contrast Mechanochromism of Anthrone-Based Donor-Acceptor Systems

*Pagidi Sudhakar, Alexandra Slawin, and Eli Zysman-Colman**

[a] Organic Semiconductor Centre, EaStCHEM School of Chemistry, University of St Andrews,

St Andrews, UK, KY16 9ST. E-mail: eli.zysman-colman@st-andrews.ac.uk

Table of contents

**Section**	**Pages**
General experimental details	S2-S4
Synthesis and spectral characterization	S5-S25
DFT results	S26-S27
Photophysical data	S27-S31
References	S31-S32

Experimental Section

General Synthetic Procedures. Reagents and solvents were obtained from commercial sources and used as received. Air-sensitive reactions were performed under a nitrogen atmosphere using Schlenk techniques, no special precautions were taken to exclude air or moisture during work-up and crystallization. Anhydrous toluene was obtained from a MBraun SPS5 solvent purification system. Flash column chromatography was carried out using silica gel (Silia-P from Silicycle, 60 Å, 40-63 µm). Analytical thin-layer-chromatography (TLC) was performed with silica plates with aluminum backings (250 µm with F-254 indicator). TLC visualization was accomplished by 254/365 nm UV lamp. HPLC analysis was conducted on a Shimadzu LC-40 HPLC system. HPLC analysis was conducted on a Shimadzu Prominence Modular HPLC system. HPLC traces were performed using an ACE Excel 2 C18 analytical column. GCMS analysis was conducted using a Shimadzu QP2010SE GC-MS equipped with a Shimadzu SH-Rtx-1 column (30 m × 0.25 mm). ^{1}H and ^{13}C spectra were recorded on a Bruker Advance spectrometer (400 MHz for ^{1}H, 125 MHz for ^{13}C). The following abbreviations have been used for multiplicity assignments: "s" for singlet, "d" for doublet, "t" for triplet and "m" for multiplet. ^{1}H and ^{13}C NMR spectra referenced residual solvent peaks with respect to TMS ($\delta = 0$ ppm). Melting points were measured using open-ended capillaries on an Electrothermal 1101D Mel-Temp apparatus and are uncorrected. High-resolution mass spectrometry (HRMS) was performed at the University of Edinburgh. Elemental analyses were performed by the School of Geosciences at the University of Edinburgh. Single-crystal XRD structures (CCDC: 2271716- 2271719) of the target compounds have been deposited.

Theoretical Calculations. All ground-state optimizations have been carried out at the Density Functional Theory (DFT) level with Gaussian16 (Frisch et al., 2016) using the PBE0 functional (Adamo and Barone, 1999) and the 6-31G(d,p) basis set (Petersson et al., 1991). Excited-state calculations have been performed at Time-Dependent DFT (TD-DFT) within the Tamm-Dancoff approximation (TDA) (Hirata and Head-Gordon, 1999) using the same functional and basis set as for ground state geometry optimization. Spin-orbit coupling matrix elements (ξ) were calculated based on the optimized singlet excited state geometry. Molecular orbitals were visualized using GaussView 6.0 (Dennington et al., 2016). Calculations were automated using an in-house designed software package, *Silico*, which uses a number of 3rd party libraries and programs, including

extraction and processing of results: cclib (Allouche, 2011), generations of 3D images (Humphrey et al., 1996): VMD & Tachyon (Edward et al., 1998).

Electrochemistry measurements. Cyclic Voltammetry (CV) analysis was performed on an Electrochemical Analyzer potentiostat model 620E from CH Instruments at a sweep rate of 100 mV/s. Differential pulse voltammetry (DPV) was conducted with an increment potential of 0.004 V and pulse amplitude, width, and period of 50 mV, 0.05, and 0.5 s, respectively. Samples were prepared as DCM solutions, which were degassed by sparging with MeCN-saturated argon gas for 5 minutes prior to measurements. All measurements were performed using 0.1 M DCM solution of tetra-*n*-butylammonium hexafluorophosphate ($[^{n}Bu_4N]PF_6$]). An Ag/Ag^+ electrode was used as the reference electrode while a platinum electrode and a platinum wire were used as the working electrode and counter electrode, respectively. The redox potentials are reported relative to a saturated calomel electrode (SCE) with a ferrocenium/ferrocene (Fc/Fc^+) redox couple as the internal standard (0.46 V vs SCE) (Pavlishchuk and Addison, 2000).

Photophysical measurements. Optically dilute solutions of concentrations on the order of 10^{-5} or 10^{-6} M were prepared in spectroscopic or HPLC grade solvents for absorption and emission analysis. Absorption spectra were recorded at room temperature on a Shimadzu UV-2600 double beam spectrophotometer with a 1 cm quartz cuvette. Molar absorptivity determination was verified by linear regression analysis of values obtained from at least four independent solutions at varying concentrations with absorbance ranging from 0.078 to 0.144 for **T-tBuCz-AQ**; 0.086 to 0.154 for **T-MeOCz-AQ**; 0.075 to 0.130 for **C-tBuCz-AQ**; 0.078 to 0.143 for **C-MeOCz-AQ;** 0.012 to 0.061 for **tBuCz-PA** and 0.002 to 0.010 for **tBuCz-DMAC**.

To prepare the 10 wt% doped films of emitters in a PMMA matrix, 90% w/w (90 mg) of host was dissolved in 1 mL of solvent and to this, 10% w/w (10 mg) of emitter was added. Thin films were then spin-coated on a quartz substrate using a spin speed of 1500 rpm for 60 s. Absolute photoluminescence quantum yields (Φ_{PL}s) were determined using an integrating sphere that is equipped with an FS5 spectrometer. The Φ_{PL}s were measured in air and N_2 environment by purging the integrating sphere with N_2 gas flow for 2 min. Steady-state PL spectra were measured using a xenon lamp as the source. Time-gated PL spectra (delayed emission/phosphorescence) were

measured using a pulsed microsecond flash lamp (μF1) by the multi-channel scaling (MCS) mode in FS5. The time-gated PL spectra for the samples were collected between 1-9 ms (λ_{exc} = 450 nm). Temperature-dependent (100 to 298 K) measurements were performed using an Oxford Instruments OPTISTAT DN-V cryostat controlled by an Oxford Instruments Mercury iTC temperature controller connected to the FS5 spectrometer. Samples were allowed to equilibrate at each temperature before measurements were conducted.

The singlet-triplet energy splitting (ΔE_{ST}) in 2-MeTHF was estimated from the onset of prompt fluorescence spectra and phosphorescence emission at 77 K. Prompt fluorescence spectra (1-100 ns) were generated using the time-resolve PL technique using a 375 nm picosecond pulsed laser diode. Prompt fluorescence lifetimes were measured using a picosecond pulsed diode laser (375 nm). Phosphorescence lifetimes were measured using a pulsed xenon microsecond flash lamp.

Fitting of time-resolved luminescence measurements: Time-resolved PL measurements were fitted to a sum of exponentials decay model, with chi-squared (χ^2) values between 1 and 2, using the EI FLS980 software. Each component of the decay is assigned a weight, (w_i), which is the contribution of the emission from each component to the total emission.

The average lifetime was then calculated using the following:

- Two exponential decay model:

$$\tau_{AVG} = \tau_1 w_1 + \tau_2 w_2$$

with weights defined as $w_1 = \frac{A1\tau_1}{A1\tau_1 + A2\tau_2}$ and $w_2 = \frac{A2\tau_2}{A1\tau_1 + A2\tau_2}$ where A1 and A2 are the preexponential-factors of each component.

- Three exponential decay model:

$$\tau_{AVG} = \tau_1 w_1 + \tau_2 w_2 + \tau_3 w_3$$

with weights defined as $w_1 = \frac{A1\tau_1}{A1\tau_1 + A2\tau_2 + A3\tau_3}$, $w_2 = \frac{A2\tau_2}{A1\tau_1 + A2\tau_2 + A3\tau_3}$ and $w_3 = \frac{A3\tau_3}{A1\tau_1 + A2\tau_2 + A3\tau_3}$ where A1, A2 and A3 are the preexponential-factors of each component.

Synthesis

General experimental procedure for Buchwald-Hartwig cross-coupling reaction: A dried Schlenk flask held under a nitrogen atmosphere was charged with dry toluene (50 mL), 1,5 dichloroanthraquinone or 1,8-dichloroanthraquinone (1 equiv.), 3,6-di-*tert*-butylcarbazole (dtBuCz) or 3,6-dimethoxy-9*H*-carbazole (2.3 equiv.), bis(dichlorophosphino)ferrocene (0.12 equiv.), $Pd_2(dba)_3$ (0.08 equiv.), and cesium carbonate (3 equiv.). The reaction mixture was then heated at 100 °C for 12 h. After cooling, the mixture was passed through a Celite pad and concentrated in a vacuum. The combined organic layer was dried with anhydrous sodium sulfate and concentrated in vacuo. The resulting mixture was purified by silica gel column chromatography to yield the desired compound.

1,5-bis(3,6-di-tert-butyl-9H-carbazol-9-yl)anthracene-9,10-dione

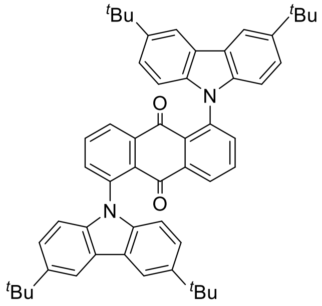

T-tBuCz-AQ

The quantities used for the reaction are as follows: 1,5 dichloroanthraquinone (700 mg, 2.53 mmol, 1 equiv.), 3,6-di-*tert*-butylcarbazole (1.62 g, 5.81 mmol, 2.3 equiv.), bis(dichlorophosphino)ferrocene (168 mg, 0.30 mmol, 0.12 equiv.), $Pd_2(dba)_3$ (185 mg, 0.20 mmol, 0.08 equiv.), cesium carbonate (2.47 g, 7.58 mmol, 3 equiv.). The target compound was then purified by silica gel column chromatography (hexane: DCM = 3:1, silica gel). Red solid. $\boldsymbol{R}_\mathbf{f}$: 0.38 (hexane:DCM = 3:2, silica gel). **Yield**: 88%. **Mp**: > 400 °C. ^{1}H NMR (500 MHz, $CDCl_3$) δ 8.25 (d, *J* = 1.4 Hz, 2H), 8.20 (dd, *J* = 6.2, 3.0 Hz, 1H), 7.93 – 7.80 (m, 2H), 7.45 (dd, *J* = 8.6, 1.9 Hz, 2H), 7.00 (d, *J* = 8.6 Hz, 2H), 1.51 (s, 18H). ^{13}C NMR (126 MHz, $CDCl_3$) δ 180.83, 142.94, 139.40, 137.29, 136.95, 136.54, 135.02, 128.96, 128.09, 123.69, 116.74, 108.86, 77.30, 77.04,

76.79, 34.79, 32.06. **HR-MS[M+Na]$^+$ Calculated: (**$C_{54}H_{54}N_2O_2Na$) 785.4083; **Found**: 785.4077. **HPLC**: 99.7%, retention time: 7.7 minutes in 95% MeCN/5% H_2O.

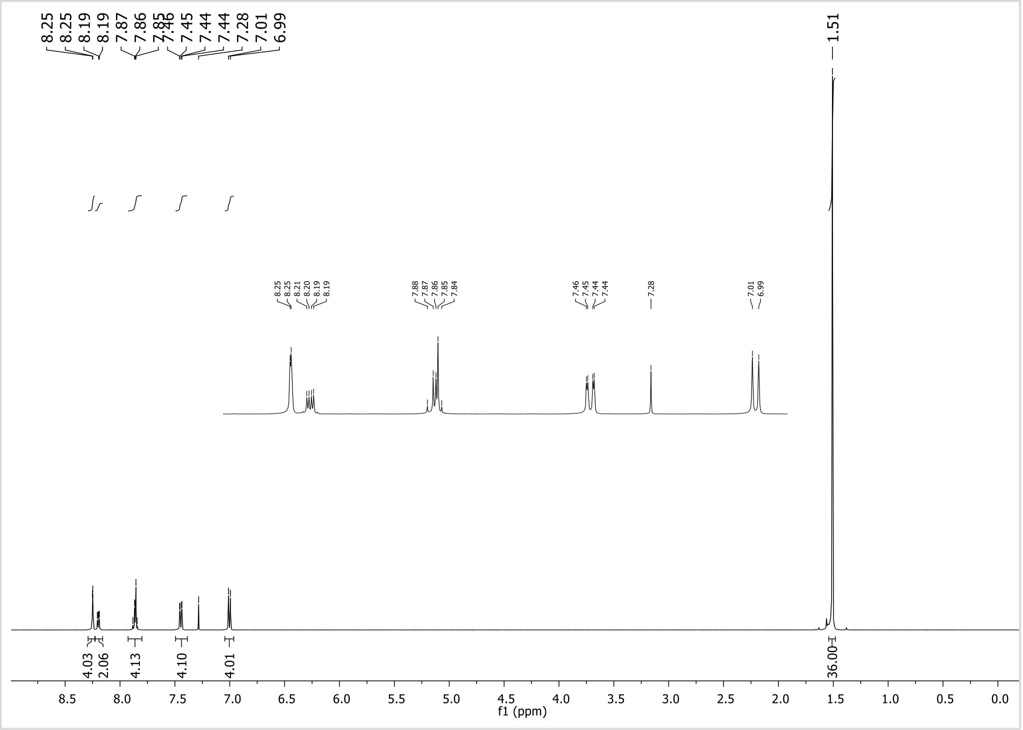

Figure **S1**. ^{1}H NMR of **T-tBuCz-AQ** in $CDCl_3$

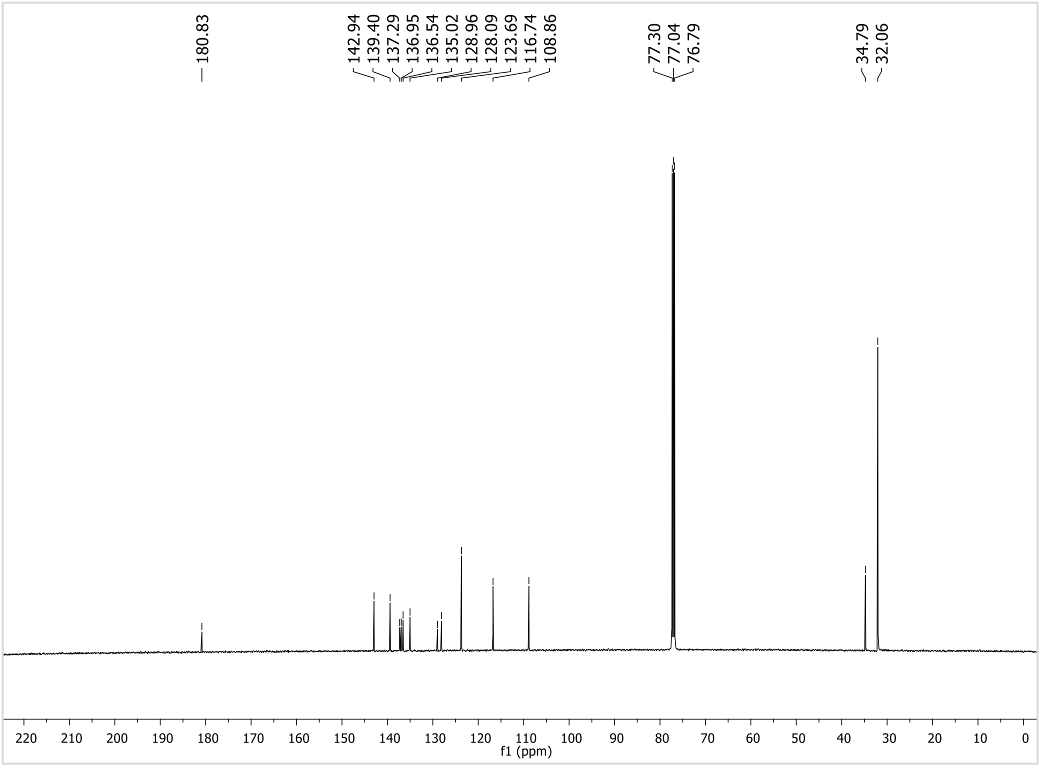

Figure **S2**. ^{13}C NMR of **T-tBuCz-AQ** in $CDCl_3$

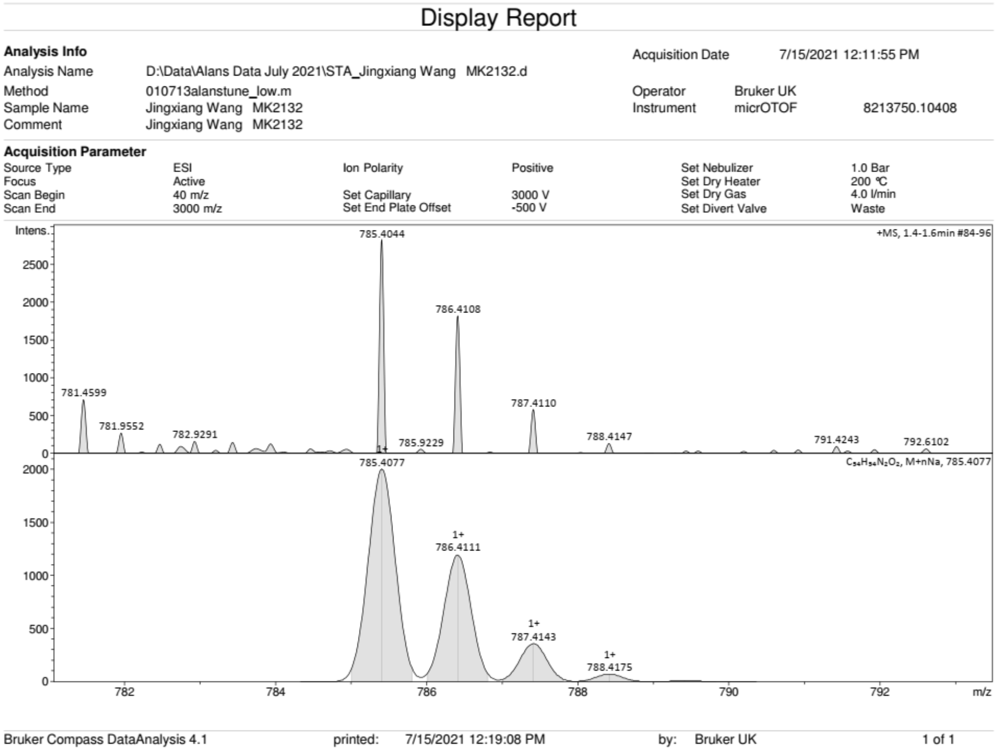

Figure **S3**. HRMS of **T-tBuCz-AQ**

HPLC Trace Report31May2023

<Sample Information>

Sample Name	: 2132		
Sample ID	:		
Method Filename	: 95% Acetonitrile 5 Water 20 mins.lcm		
Batch Filename	: ALL.lcb		
Vial #	: 1-53	Sample Type	: Unknown
Injection Volume	: 10 uL		
Date Acquired	: 30/05/2023 15:33:31	Acquired by	: System Administrator
Date Processed	: 30/05/2023 15:53:33	Processed by	: System Administrator

<Chromatogram>

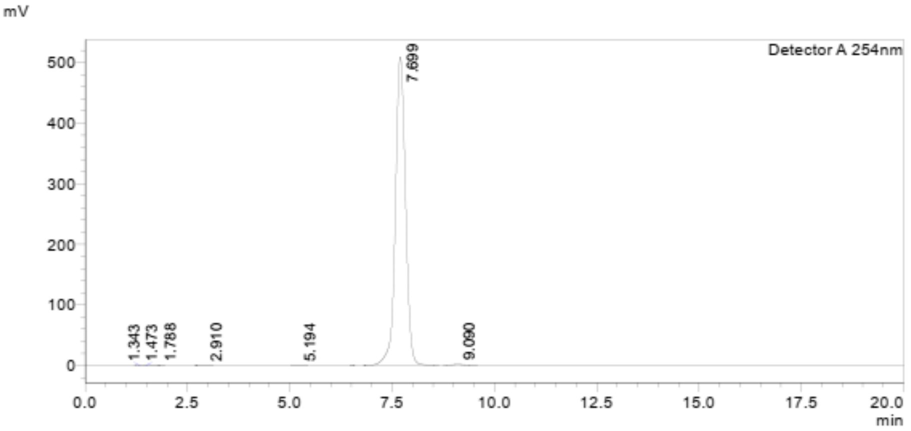

<Peak Table>

Detector A 254nm

Peak#	Ret. Time	Area	Height	Area%	Area/Height	Width at 5% Height
1	1.343	1925	257	0.022	7.481	--
2	1.473	1045	177	0.012	5.910	--
3	1.788	2643	156	0.030	16.982	--
4	2.910	3133	280	0.036	11.202	0.341
5	5.194	1495	126	0.017	11.836	0.349
6	7.699	8740085	508263	99.661	17.196	0.603
7	9.090	19469	1025	0.222	18.998	0.600
Total		8769795	510284	100.000		

Figure **S4**. HPLC trace report of **T-tBuCz-AQ**

1,8-bis(3,6-di-tert-butyl-9H-carbazol-9-yl)anthracene-9,10-dione

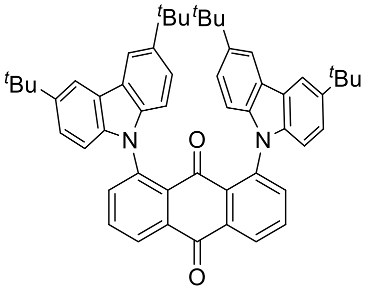

C-tBuCz-AQ

The quantities used for the reaction are as follows: 1,8 dichloroanthraquinone (800 mg, 2.89 mmol, 1 equiv.), 3,6-di-*tert*-butylcarbazole (1.85 g, 6.64 mmol, 2.3 equiv.), bis(dichlorophosphino)ferrocene (192 mg, 0.35 mmol, 0.12 equiv.), $Pd_2(dba)_3$ (211 mg, 0.23 mmol, 0.08 equiv.), cesium carbonate (2.82 g, 8.66 mmol, 3 equiv.). The target compound was then purified by silica gel column chromatography (hexane: DCM = 3:2, silica gel). Red solid. $\boldsymbol{R}_{\mathbf{f}}$: 0.51 (hexane:DCM = 2:3, silica gel). **Yield**: 60%. **Mp**: 379-382 °C. ^{1}H NMR (400 MHz, $CDCl_3$) δ 8.49 (dd, J = 7.7, 1.3 Hz, 1H), 7.84 (t, J = 7.8 Hz, 1H), 7.67 (dd, J = 7.9, 1.3 Hz, 1H), 7.19 (dd, J = 8.6, 1.9 Hz, 2H), 6.91 (d, J = 8.6 Hz, 2H), 1.36 (s, 18H). ^{13}C NMR (126 MHz, $CDCl_3$) δ 183.33, 180.05, 142.84, 139.97, 138.38, 136.58, 134.98, 133.91, 131.63, 126.75, 124.24, 123.27, 116.33, 109.84, 77.29, 77.04, 76.78, 34.59, 31.96. **HR-MS[M+Na]$^{+}$ Calculated: (**$C_{54}H_{54}N_2O_2Na$) 785.4083; **Found**: 785.4077. **HPLC**: 99.8%, retention time: 13 minutes in 95% MeCN/5% H_2O.

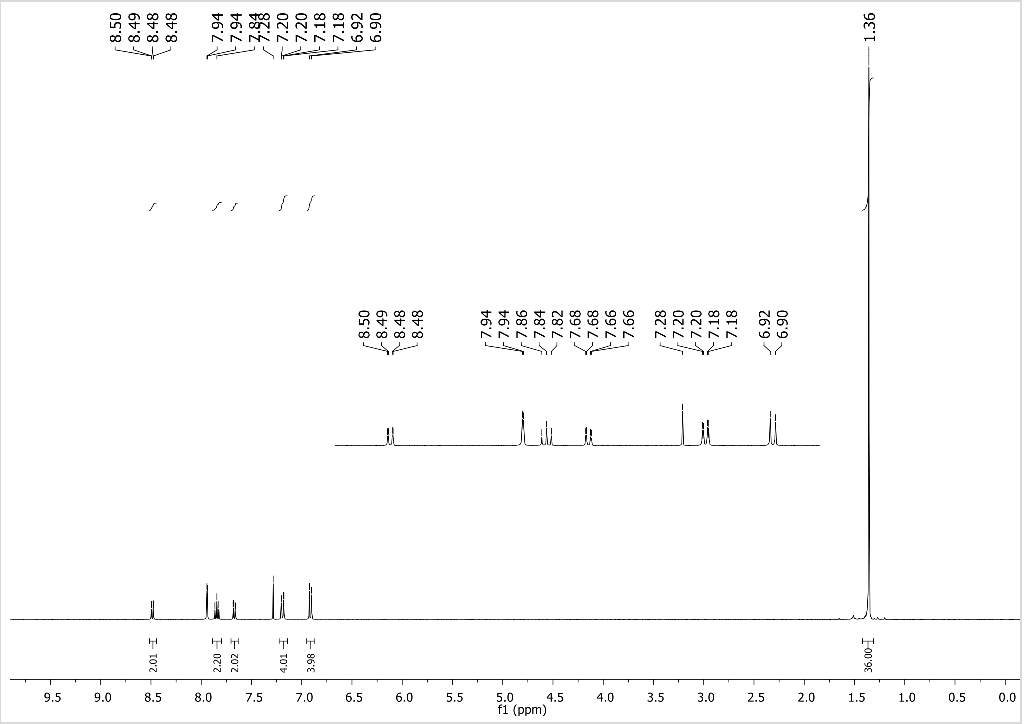

Figure **S5**. ^{1}H NMR of **C-tBuCz-AQ** in $CDCl_3$

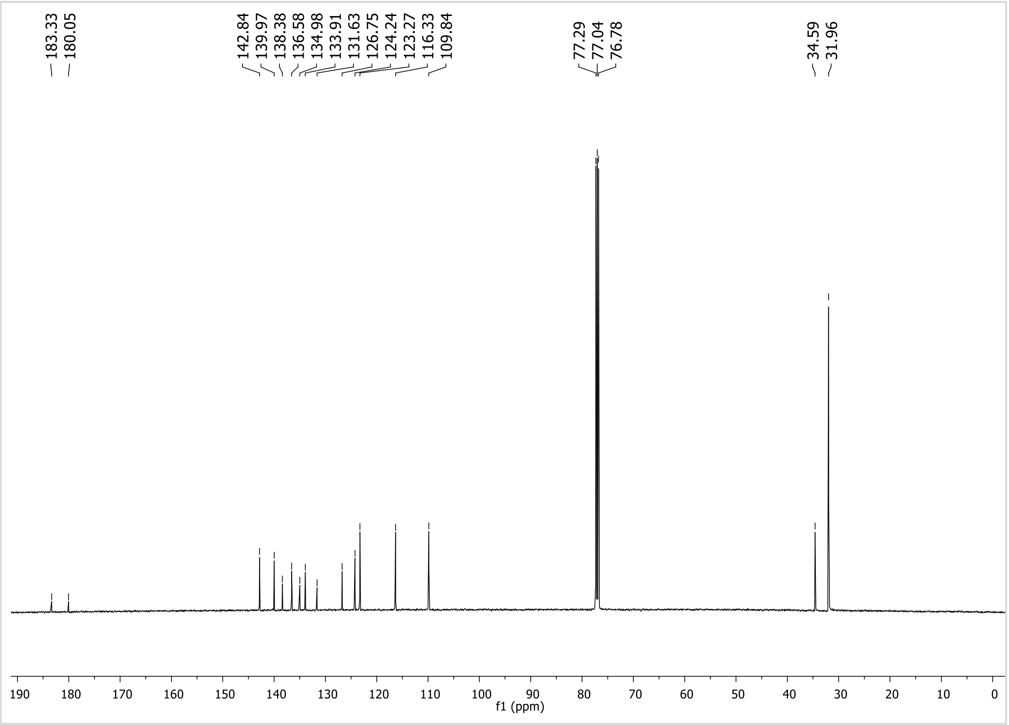

Figure **S6**. ^{13}C NMR of **C-tBuCz-AQ** in $CDCl_3$

Display Report

Analysis Info

Analysis Name	D:\Data\Alans Data July 2021\STA_Jingxiang Wang MK2205.d	Acquisition Date	7/15/2021 12:22:02 PM
Method	010713alanstune_low.m	Operator	Bruker UK
Sample Name	Jingxiang Wang MK2205	Instrument	micrOTOF 8213750.10408
Comment	Jingxiang Wang MK2205		

Acquisition Parameter

Source Type	ESI	Ion Polarity	Positive	Set Nebulizer	1.0 Bar
Focus	Active			Set Dry Heater	200 °C
Scan Begin	40 m/z	Set Capillary	3000 V	Set Dry Gas	4.0 l/min
Scan End	3000 m/z	Set End Plate Offset	-500 V	Set Divert Valve	Waste

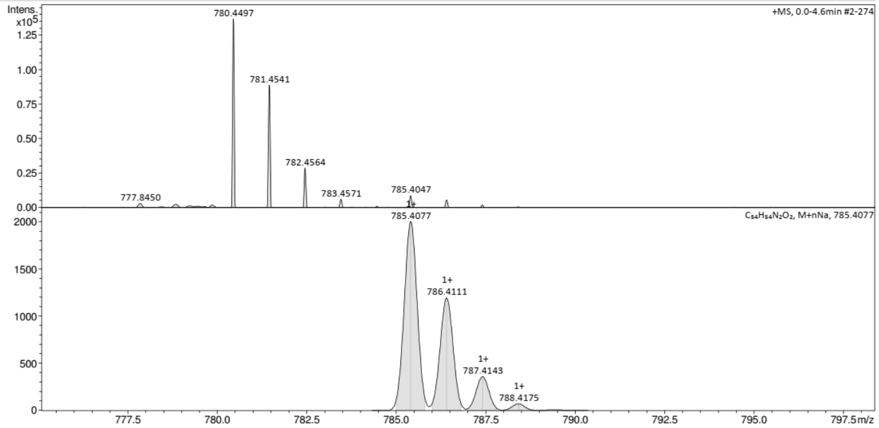

Bruker Compass DataAnalysis 4.1 printed: 7/15/2021 12:32:52 PM by: Bruker UK 1 of 1

Figure **S7**. HRMS of **C-tBuCz-AQ**

HPLC Trace Report31May2023

<Sample Information>

Sample Name	: 2205		
Sample ID	:		
Method Filename	: 95% Acetonitrile 5 Water 20 mins.lcm		
Batch Filename	: ALL.lcb		
Vial #	: 1-51	Sample Type	: Unknown
Injection Volume	: 10 uL		
Date Acquired	: 30/05/2023 16:35:08	Acquired by	: System Administrator
Date Processed	: 30/05/2023 16:55:09	Processed by	: System Administrator

<Chromatogram>

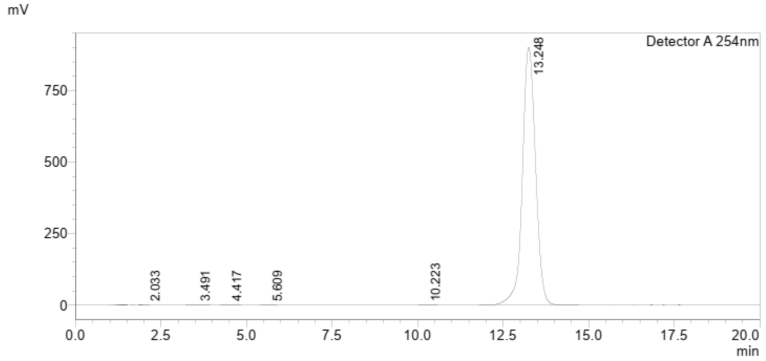

<Peak Table>

Detector A 254nm

Peak#	Ret. Time	Area	Height	Area%	Area/Height	Width at 5% Height
1	2.033	1147	109	0.005	10.545	--
2	3.491	17536	1079	0.073	16.255	0.588
3	4.417	11236	1047	0.047	10.730	0.383
4	5.609	2349	163	0.010	14.382	0.410
5	10.223	2506	149	0.010	16.851	0.465
6	13.248	24118710	900075	99.856	26.796	0.949
Total		24153483	902622	100.000		

Figure **S8**. HPLC trace report of **C-tBuCz-AQ**

1,5-bis(3,6-dimethoxy-9H-carbazol-9-yl)anthracene-9,10-dione

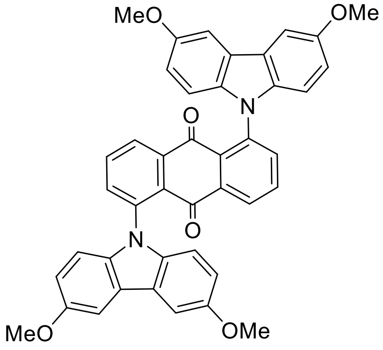

T-MeOCz-AQ

The quantities used for the reaction are as follows: 1,5 dichloroanthraquinone (600 mg, 2.16 mmol, 1 equiv.), 3,6-dimethoxy-9*H*-carbazole (1.13 g, 4.98 mmol, 2.3 equiv.), bis(dichlorophosphino)ferrocene (144 mg, 0.26 mmol, 0.12 equiv.), $Pd_2(dba)_3$ (159 mg, 0.17 mmol, 0.08 equiv.), cesium carbonate (2.1 g, 6.49 mmol, 3 equiv.). The target compound was then purified by silica gel column chromatography (hexane: EtOAc = 2:1, silica gel). Purple solid. $\boldsymbol{R_f}$: 0.36 (hexane: EtOAc = 1:1, silica gel). **Yield**: 86%. **Mp**: 321-323 °C. ^{1}H NMR (400 MHz, $CDCl_3$) δ 8.51 (dd, J = 7.8, 1.3 Hz, 1H), 7.89 (t, J = 7.8 Hz, 1H), 7.71 (dd, J = 7.8, 1.3 Hz, 1H), 7.35 (t, J = 1.4 Hz, 2H), 6.78 (d, J = 1.5 Hz, 4H), 3.88 (s, 6H). ^{13}C NMR (126 MHz, $CDCl_3$) δ 180.78, 154.19, 137.18, 136.98, 136.45, 136.35, 135.06, 128.80, 127.96, 124.10, 115.25, 110.40, 103.30, 77.29, 77.04, 76.79, 56.10. **HR-MS[M+H]$^+$ Calculated: (**$C_{42}H_{31}N_2O_6$) 659.2177; **Found**: 659.2177. **HPLC**: 99.9%, retention time: 2.1 minutes in 95% MeCN/5% H_2O.

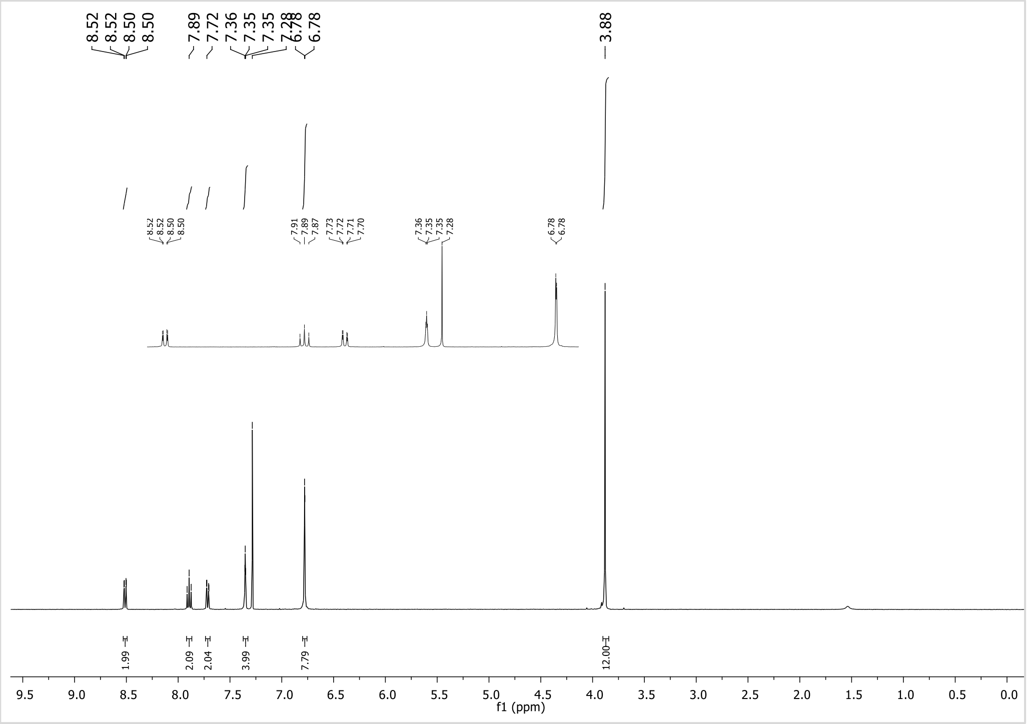

Figure **S9**. ^{1}H NMR of **T-MeOCz-AQ** in $CDCl_3$

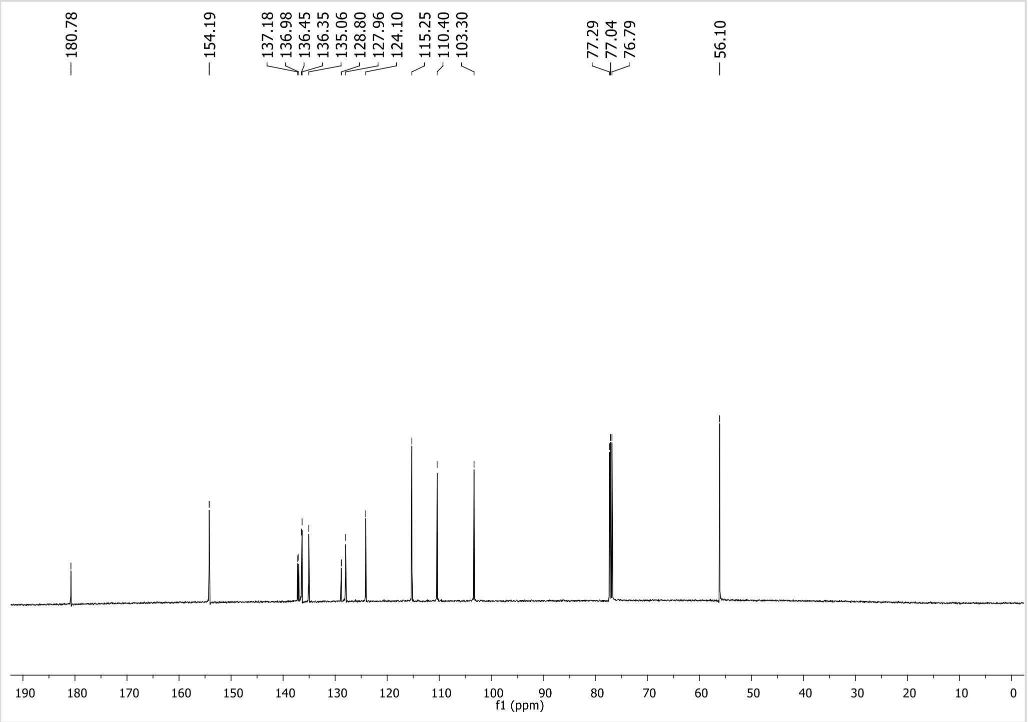

Figure **S10**. ^{13}C NMR of **T-MeOCz-AQ** in $CDCl_3$

Display Report

Analysis Info

Analysis Name	D:\Data\Alans Data July 2021\STA_Jingxiang Wang MK2322.d	Acquisition Date	7/15/2021 11:47:56 AM
Method	010713alanstune_low.m	Operator	Bruker UK
Sample Name	Jingxiang Wang MK2322	Instrument	micrOTOF 8213750.10408
Comment	Jingxiang Wang MK2322		

Acquisition Parameter

Source Type	ESI	Ion Polarity	Positive	Set Nebulizer	1.0 Bar
Focus	Active			Set Dry Heater	200 °C
Scan Begin	40 m/z	Set Capillary	3000 V	Set Dry Gas	4.0 l/min
Scan End	3000 m/z	Set End Plate Offset	-500 V	Set Divert Valve	Waste

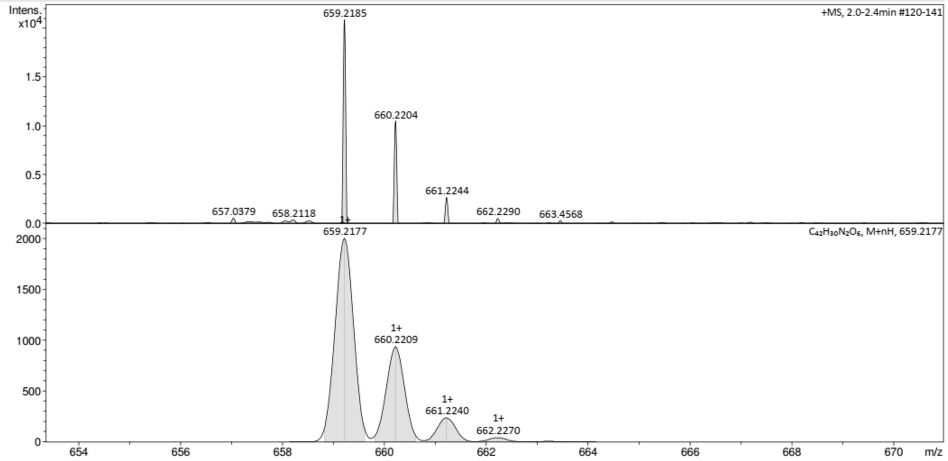

Bruker Compass DataAnalysis 4.1 printed: 7/15/2021 11:56:45 AM by: Bruker UK 1 of 1

Figure **S11**. HRMS of **T-MeOCz-AQ**

HPLC Trace Report31May2023

<Sample Information>

Sample Name	: 2322		
Sample ID	:		
Method Filename	: 95% Acetonitrile 5 Water 20 mins.lcm		
Batch Filename	: ALL.lcb		
Vial #	: 1-52	Sample Type	: Unknown
Injection Volume	: 10 uL		
Date Acquired	: 30/05/2023 16:04:18	Acquired by	: System Administrator
Date Processed	: 30/05/2023 16:24:21	Processed by	: System Administrator

<Chromatogram>

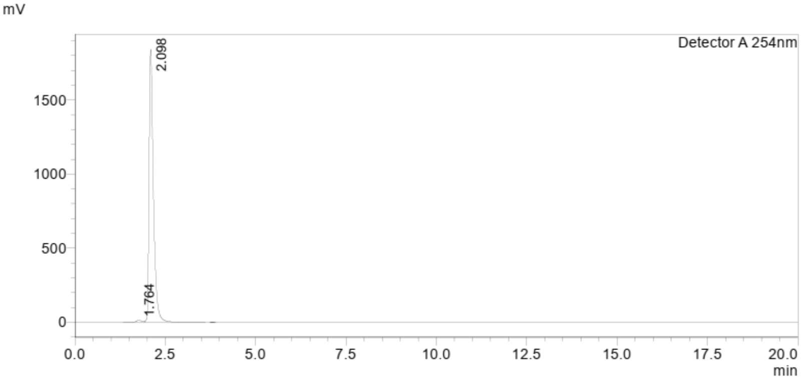

<Peak Table>

Detector A 254nm

Peak#	Ret. Time	Area	Height	Area%	Area/Height	Width at 5% Height
1	1.764	139137	12144	0.896	11.457	--
2	2.098	15381500	1838236	99.104	8.368	0.302
Total		15520637	1850380	100.000		

Figure **S12**. HPLC trace report of **T-MeOCz-AQ**

1,8-bis(3,6-dimethoxy-9H-carbazol-9-yl)anthracene-9,10-dione

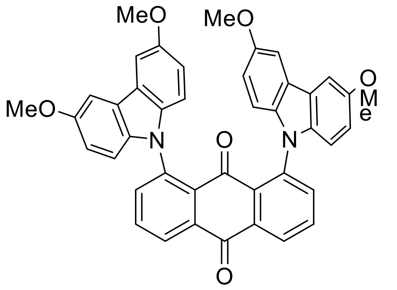

C-MeOCz-AQ

The quantities used for the reaction are as follows: 1,5 dichloroanthraquinone (800 mg, 2.89 mmol, 1 equiv.), 3,6-dimethoxy-9*H*-carbazole (1.51 g, 6.64 mmol, 2.3 equiv.), bis(dichlorophosphino)ferrocene (192 mg, 0.35 mmol, 0.12 equiv.), $Pd_2(dba)_3$ (211 mg, 0.23 mmol, 0.08 equiv.), cesium carbonate (2.8 g, 8.66 mmol, 3 equiv.). The target compound was then purified by silica gel column chromatography (hexane: EtOAc = 2:1, silica gel). Purple solid. $\boldsymbol{R}_f$: 0.47 (hexane: EtOAc = 1:1, silica gel). **Yield**: 63%. **Mp**: 314-317 °C. ^{1}H NMR (400 MHz, $CDCl_3$) δ 8.51 (dd, *J* = 7.8, 1.3 Hz, 1H), 7.89 (t, *J* = 7.8 Hz, 1H), 7.71 (dd, *J* = 7.8, 1.3 Hz, 1H), 7.35 (t, *J* = 1.4 Hz, 2H), 6.78 (d, *J* = 1.5 Hz, 4H), 3.88 (s, 6H). ^{13}C NMR (126 MHz, $CDCl_3$) δ 182.89, 179.71, 153.82, 137.63, 137.26, 136.56, 135.04, 134.18, 131.58, 127.17, 124.01, 114.79, 110.37, 103.13, 77.29, 77.04, 76.78, 55.97. **HR-MS[M+Na]$^+$ Calculated: (**$C_{42}H_{30}N_2O_6Na$) 681.1997; **Found**: 681.1996. **HPLC**: 98.9%, retention time: 2.3 minutes in 95% MeCN/5% H_2O.

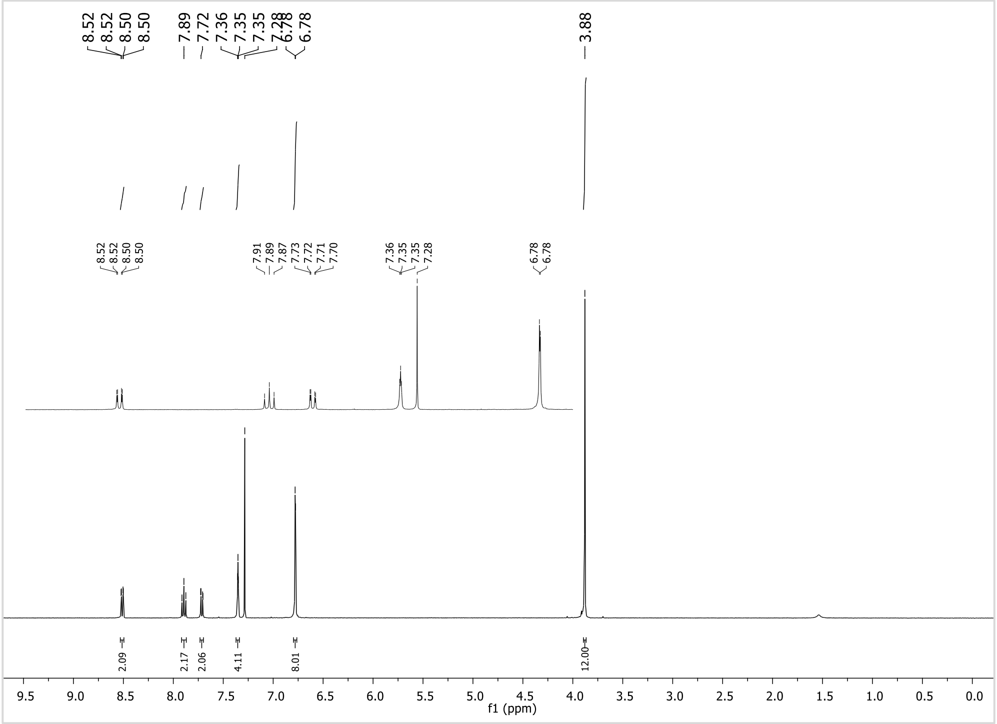

Figure **S13**. ^{1}H NMR of **C-MeOCz-AQ** in $CDCl_3$

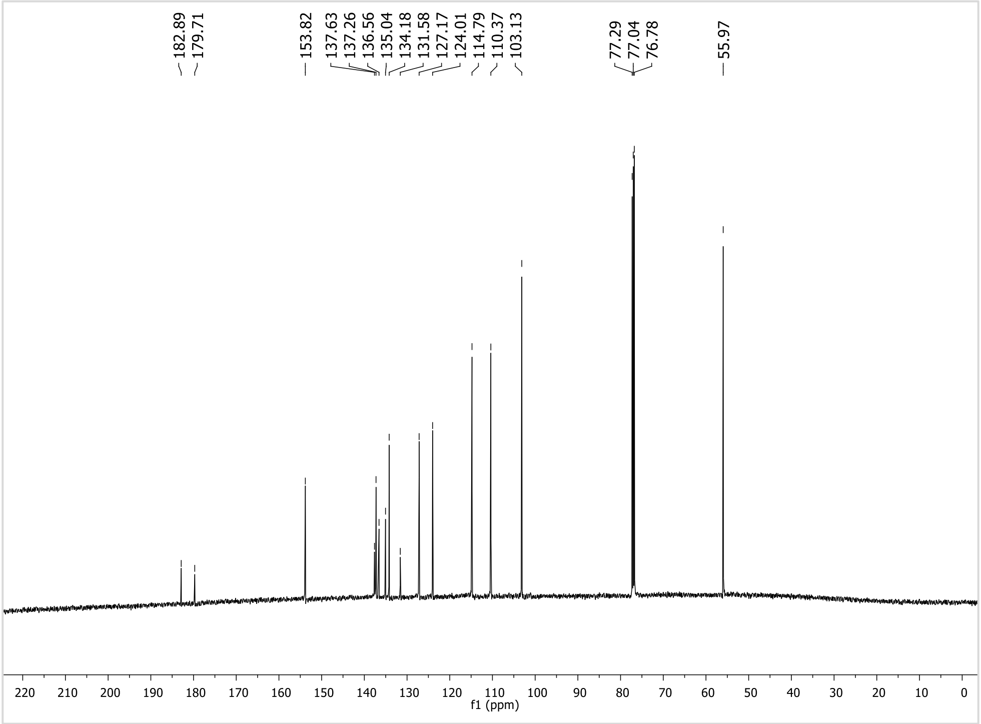

Figure **S14**. ^{13}C NMR of **C-MeOCz-AQ** in $CDCl_3$

Display Report

Analysis Info

Analysis Name	D:\Data\Alans Data July 2021\STA_Jingxiang Wang MK2397.d	Acquisition Date	7/15/2021 12:00:35 PM
Method	010713alanstune_low.m	Operator	Bruker UK
Sample Name	Jingxiang Wang MK2397	Instrument	micrOTOF 8213750.10408
Comment	Jingxiang Wang MK2397		

Acquisition Parameter

Source Type	ESI	Ion Polarity	Positive	Set Nebulizer	1.0 Bar
Focus	Active			Set Dry Heater	200 °C
Scan Begin	40 m/z	Set Capillary	3000 V	Set Dry Gas	4.0 l/min
Scan End	3000 m/z	Set End Plate Offset	-500 V	Set Divert Valve	Waste

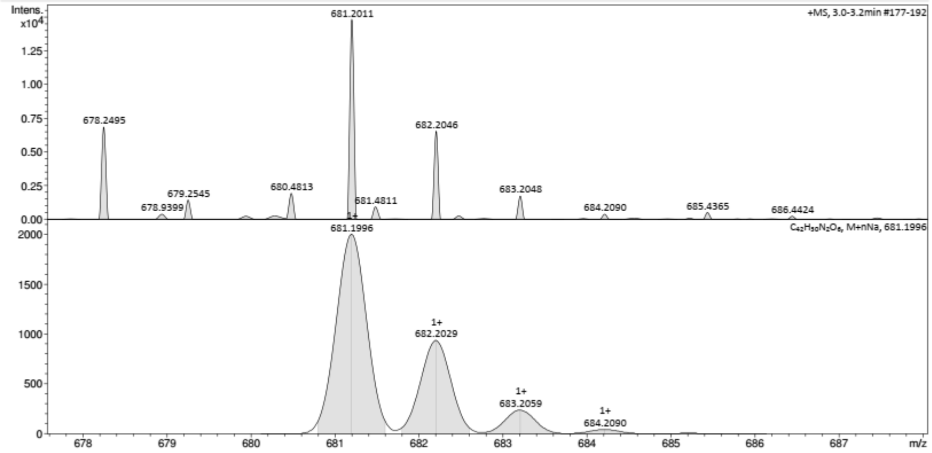

Bruker Compass DataAnalysis 4.1 printed: 7/15/2021 12:08:59 PM by: Bruker UK 1 of 1

Figure **S15**. HRMS **C-MeOCz-AQ**

HPLC Trace Report31May2023

<Sample Information>

Sample Name	: 2397		
Sample ID	:		
Method Filename	: 95% Acetonitrile 5 Water 20 mins.lcm		
Batch Filename	: ALL.lcb		
Vial #	: 1-50	Sample Type	: Unknown
Injection Volume	: 10 uL		
Date Acquired	: 30/05/2023 17:18:47	Acquired by	: System Administrator
Date Processed	: 30/05/2023 17:38:50	Processed by	: System Administrator

<Chromatogram>

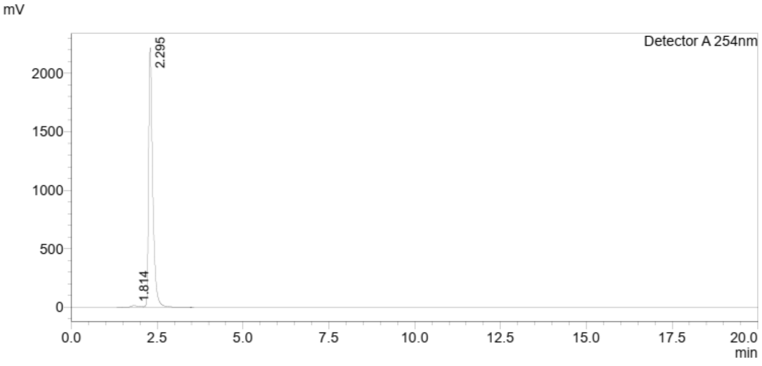

<Peak Table>

Detector A 254nm

Peak#	Ret. Time	Area	Height	Area%	Area/Height	Width at 5% Height
1	1.814	196113	13098	1.063	14.973	--
2	2.295	18244666	2214120	98.937	8.240	0.301
Total		18440779	2227218	100.000		

Figure **S16**. HPLC trace report of **C-MeOCz-AQ**

General experimental procedure for Buchwald-Hartwig cross-coupling reaction: An oven-An oven-dried Schlenk flask held under a nitrogen atmosphere was charged with dry toluene (50 mL), 1,5 2-butyl-7-chlorodibenzo[cd,g]indazol-6(2H)-one (1 equiv.), 3,6-di-*tert*-butylcarbazole (dtBuCz) or DMAC (1.1 equiv.), bis(dichlorophosphino)ferrocene (0.12 equiv.), $Pd_2(dba)_3$ (0.08 equiv.), and cesium carbonate (3 equiv.). The reaction mixture was then heated at 100 °C for 12 h. After cooling, the mixture was passed through a Celite pad and concentrated in vacuo. The combined organic layer was dried with anhydrous sodium sulfate and concentrated in vacuo. The resulting mixture was purified by silica gel column chromatography to yield the desired compound.

2-butyl-7-(3,6-di-tert-butyl-9H-carbazol-9-yl)dibenzo[cd,g]indazol-6(2H)-one

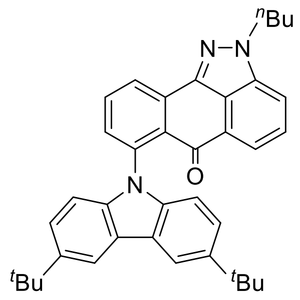

tBuCz-PA

The quantities used for the reaction are as follows: 1,5 2-butyl-7-chlorodibenzo[cd,g]indazol-6(2H)-one (200 mg, 0.64 mmol, 1 equiv.), 3,6-di-*tert*-butylcarbazole (198 mg, 51 µmmol, 1.1 equiv.), bis(dichlorophosphino)ferrocene (43 mg, 77 µmol, 0.12 equiv.), $Pd_2(dba)_3$ (30 mg, 0.17 mmol, 0.08 equiv.), cesium carbonate (629 mg, 1.93 mmol, 3 equiv.). The target compound was then purified by silica gel column chromatography (hexane: EtOAc = 4:1, silica gel). Yellow solid. ***R*$_f$**: 0.59 (hexane: EtOAc = 3:1, silica gel). **Yield**: 84%. **Mp**: 287-290 °C. ^{1}H NMR (400 MHz, $CDCl_3$) δ 8.49 (dd, *J* = 7.8, 1.3 Hz, 1H), 8.22 (d, *J* = 1.5 Hz, 2H), 7.88 (t, *J* = 7.8 Hz, 1H), 7.72 (d, *J* = 7.0 Hz, 1H), 7.67 (d, *J* = 8.2 Hz, 1H), 7.54 (ddd, *J* = 7.1, 4.5, 3.1 Hz, 2H), 7.38 (dd, *J* = 8.6, 1.9 Hz, 2H), 6.98 (d, *J* = 8.6 Hz, 2H), 4.58 (t, *J* = 7.1 Hz, 2H), 2.07 (dq, *J* = 12.7, 7.3 Hz, 2H), 1.48 (s, 18H), 1.03 (t, *J* = 7.4 Hz, 3H). ^{13}C NMR (101 MHz, $CDCl_3$) δ 181.80, 142.01, 140.03, 139.55, 139.05, 138.40, 134.75, 134.00, 131.69, 129.87, 128.38, 127.01, 123.40, 123.37, 123.03, 120.88,

116.56, 114.47, 108.81, 77.37, 77.26, 77.05, 76.74, 50.00, 34.72, 32.50, 32.10, 20.18, 13.73. **HR-MS[M+H]+ Calculated: (**$C_{38}H_{40}N_3O$) 554.3166; **Found**: 554.3166. **HPLC**: 99.93%, retention time: 4.91 minutes in 100% MeCN

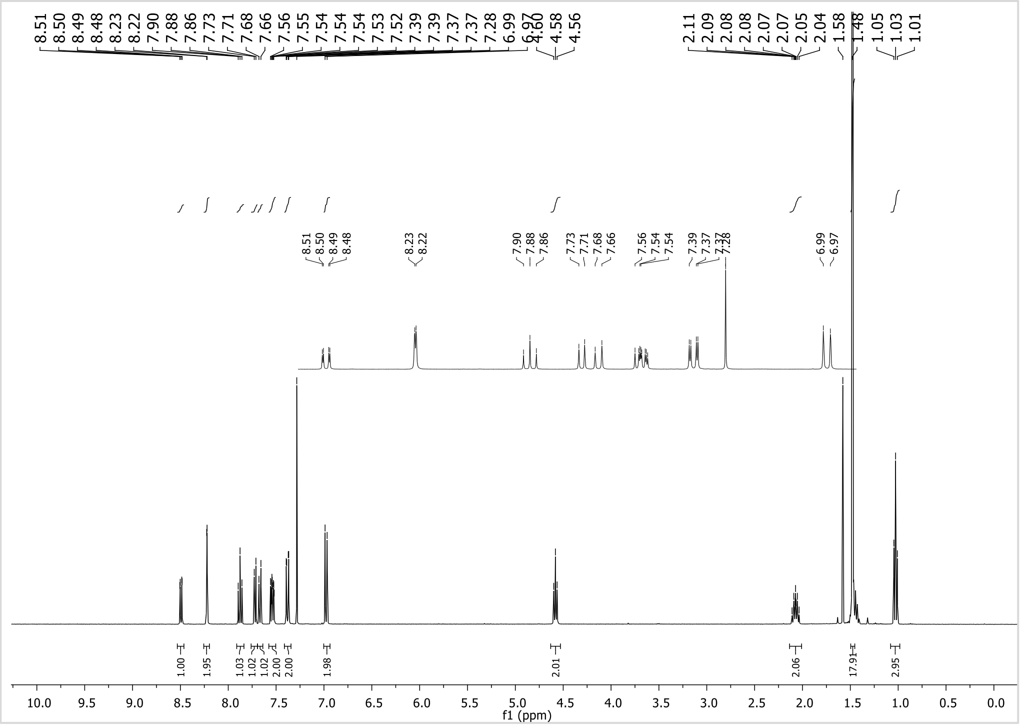

Figure **S17**. ^{1}H NMR of **tBuCz-PA** in $CDCl_3$

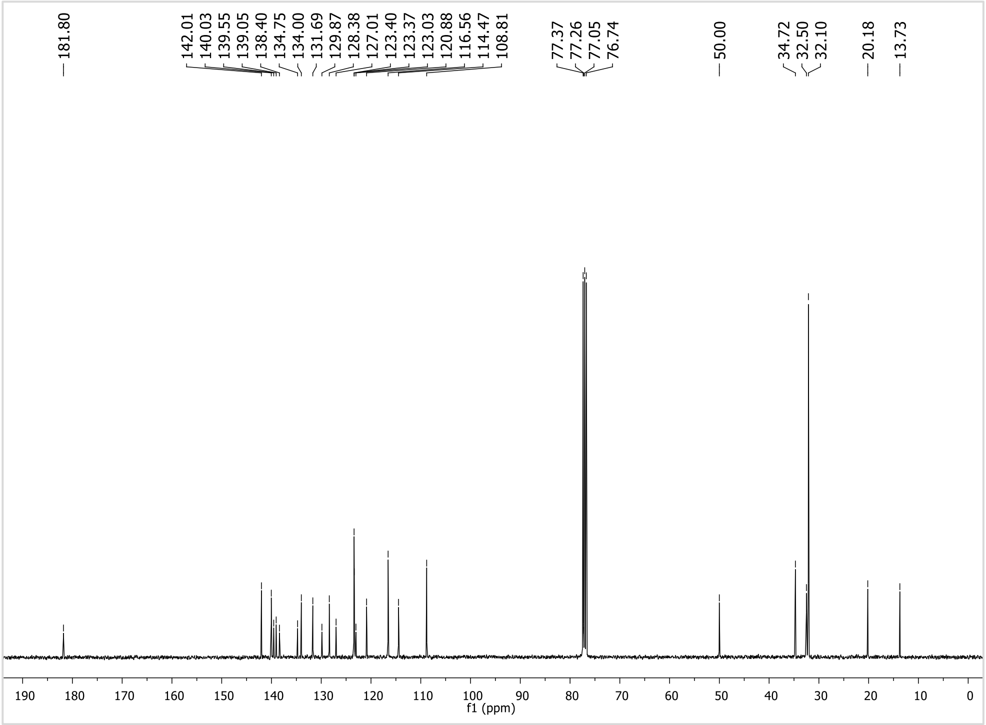

Figure **S18**. ^{13}C NMR of **tBuCz-PA** in $CDCl_3$

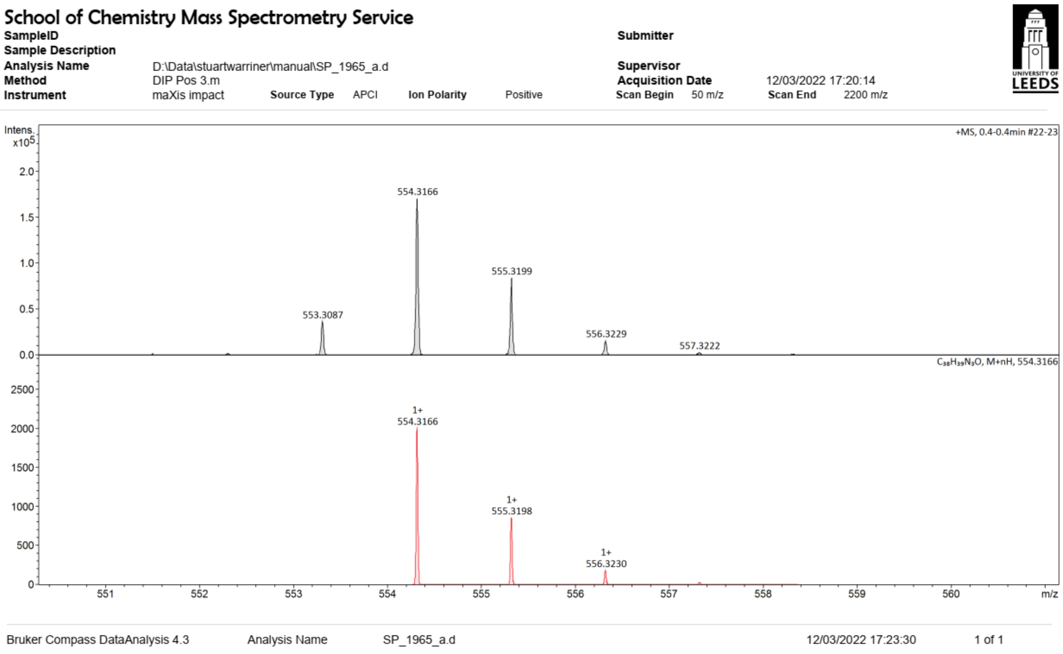

Figure **S19**. HRMS of **tBuCz-PA**

HPLC Trace Report16Jun2023

<Sample Information>

Sample Name	: 1965		
Sample ID	:		
Method Filename	: 100% Acetonitrile B 20 mins.lcm		
Batch Filename	: 1965-R.lcb		
Vial #	: 1-53	Sample Type	: Unknown
Injection Volume	: 10 uL		
Date Acquired	: 14/06/2023 15:13:44	Acquired by	: System Administrator
Date Processed	: 14/06/2023 15:33:46	Processed by	: System Administrator

<Chromatogram>

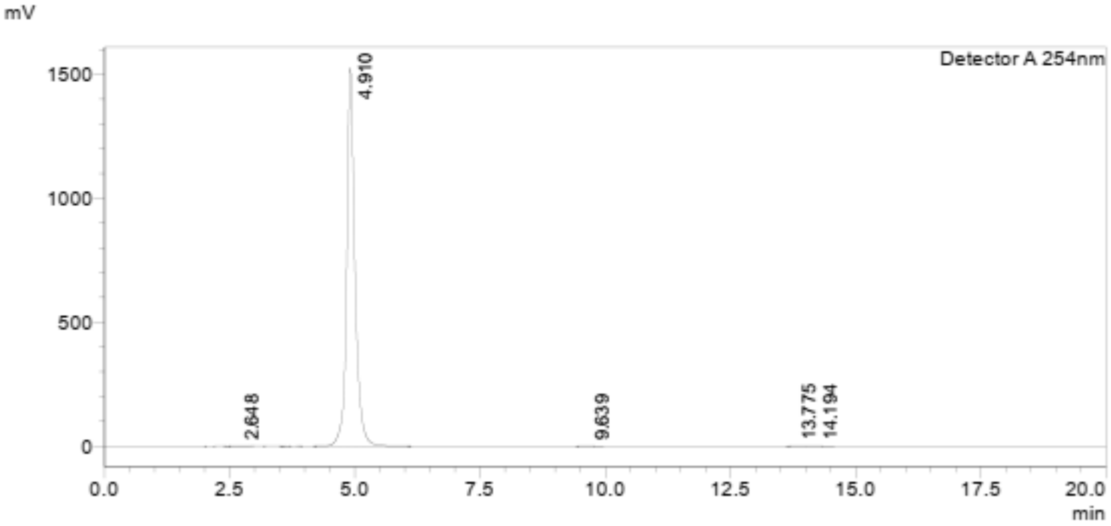

<Peak Table>

Detector A 254nm

Peak#	Ret. Time	Area	Height	Area%	Area/Height	Width at 5% Height
1	2.648	3020	196	0.017	15.428	--
2	4.910	17401746	1521603	99.929	11.436	0.466
3	9.639	3581	231	0.021	15.524	0.461
4	13.775	2031	92	0.012	21.977	--
5	14.194	3773	196	0.022	19.259	--
Total		17414151	1522318	100.000		

Figure **S20**. HPLC trace report of **tBuCz-PA**

2-butyl-7-(9,9-dimethylacridin-10(9H)-yl)dibenzo[cd,g]indazol-6(2H)-one

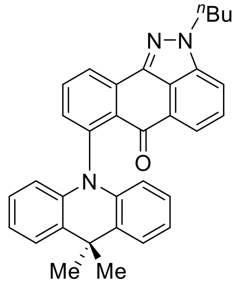

DMAC-PA

The quantities used for the reaction are as follows: 2-butyl-7-chlorodibenzo[cd,g]indazol-6(2H)-one (200 mg, 0.64 mmol, 1 equiv.), DMAC (148 mg, 0.71 mmol, 1.1 equiv.), bis(dichlorophosphino)ferrocene (43 mg, 77 µmol, 0.12 equiv.), $Pd_2(dba)_3$ (30 mg, 0.17 mmol, 0.08 equiv.), cesium carbonate (629 mg, 1.93 mmol, 3 equiv.). The target compound was then purified by silica gel column chromatography (hexane: EtOAc = 4:1, silica gel). Black solid. $\boldsymbol{R_f}$: 0.53 (hexane: EtOAc = 3:1, silica gel). **Yield**: 87%. **Mp**: 268-270 °C. ^{1}H NMR (400 MHz, $CDCl_3$) δ 8.48 (dd, J = 7.8, 1.4 Hz, 1H), 7.96 – 7.88 (m, 2H), 7.68 (d, J = 8.2 Hz, 1H), 7.63 – 7.55 (m, 1H), 7.44 (dd, J = 7.7, 1.4 Hz, 1H), 6.74 (dd, J = 7.9, 1.5 Hz, 2H), 6.61 (td, J = 7.7, 1.5 Hz, 2H), 6.50 (td, J = 7.7, 1.5 Hz, 2H), 5.80 (dd, J = 7.9, 1.5 Hz, 2H), 4.57 (t, J = 7.1 Hz, 2H), 2.10 – 2.00 (m, 2H), 1.51 – 1.38 (m, 2H), 1.01 (t, J = 7.4 Hz, 3H). ^{13}C NMR (101 MHz, $CDCl_3$) δ 181.53, 144.27, 139.87, 139.06, 138.22, 135.90, 135.36, 134.31, 134.06, 130.79, 128.44, 126.86, 124.03, 123.03, 121.19, 120.89, 115.44, 114.59, 112.20, 77.36, 77.05, 76.73, 50.01, 32.48, 20.15, 13.71. **HR-MS[M-2CH$_3$-CH$_2$+3H]$^+$ Calculated: (**$C_{38}H_{40}N_3O$) 554.3166; **Found**: 554.3166. **HPLC**: 98.9%, retention time: 2.7 minutes in 90% MeCN/10% H_2O.

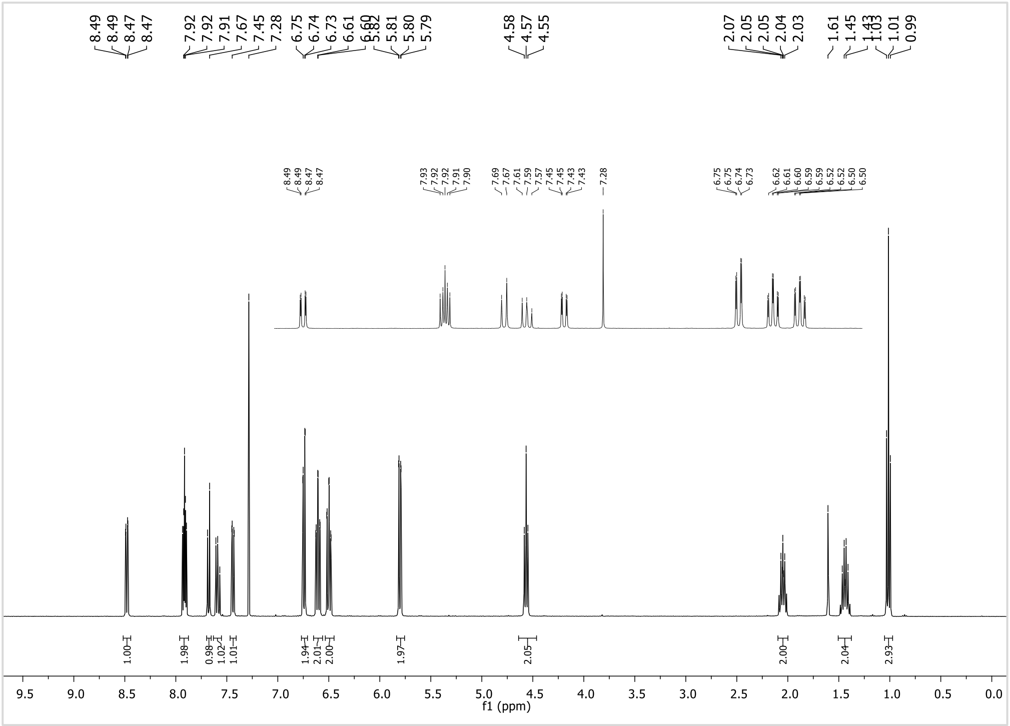

Figure **S21**. ^{1}H NMR of **DMAC-PA** in $CDCl_3$

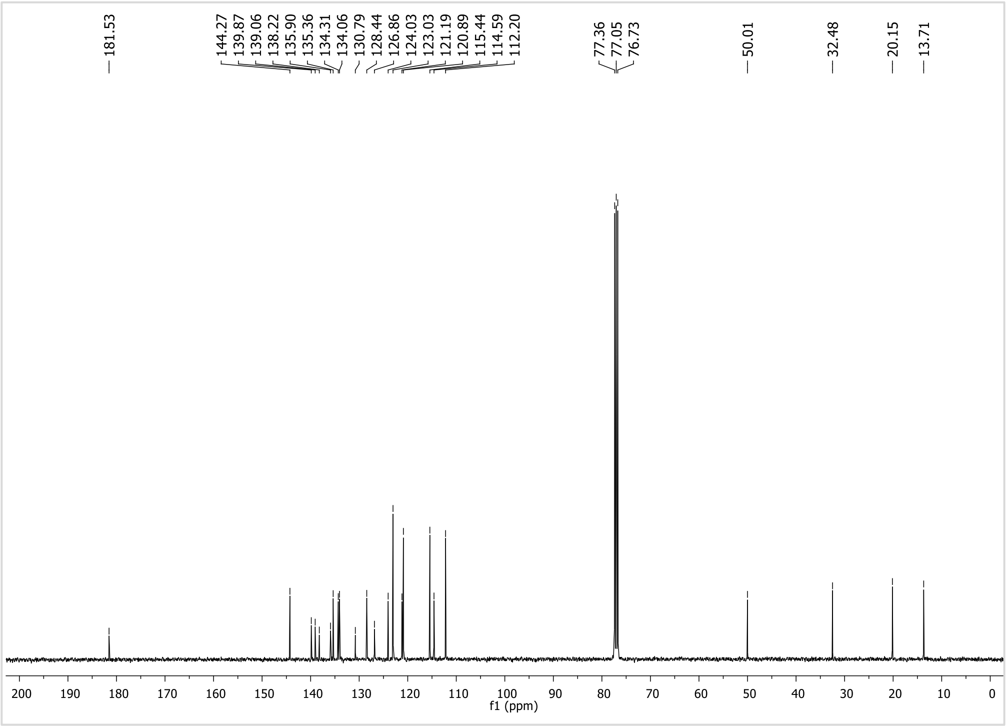

Figure **S22**. ^{13}C NMR of **DMAC-PA** in $CDCl_3$

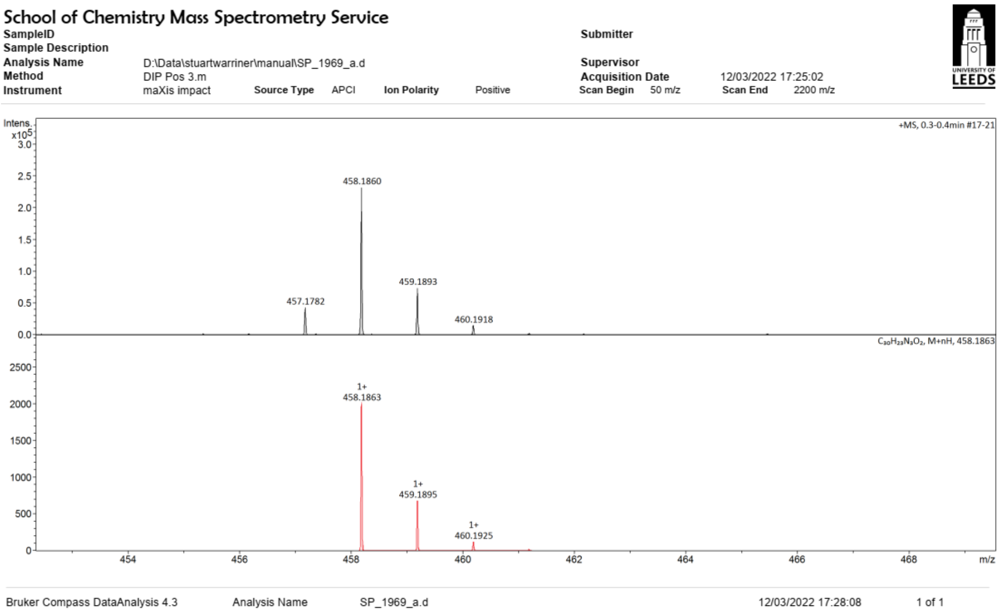

Figure **S23**. HRMS of **DMAC-PA**

HPLC Trace Report12Jun2023

<Sample Information>

Sample Name	: 1969-R		
Sample ID	:		
Method Filename	: 95% Acetonitrile 5 Water 20 mins.lcm		
Batch Filename	: REPEATION.lcb		
Vial #	: 1-52	Sample Type	: Unknown
Injection Volume	: 10 uL		
Date Acquired	: 11/06/2023 19:49:21	Acquired by	: System Administrator
Date Processed	: 11/06/2023 20:09:23	Processed by	: System Administrator

<Chromatogram>

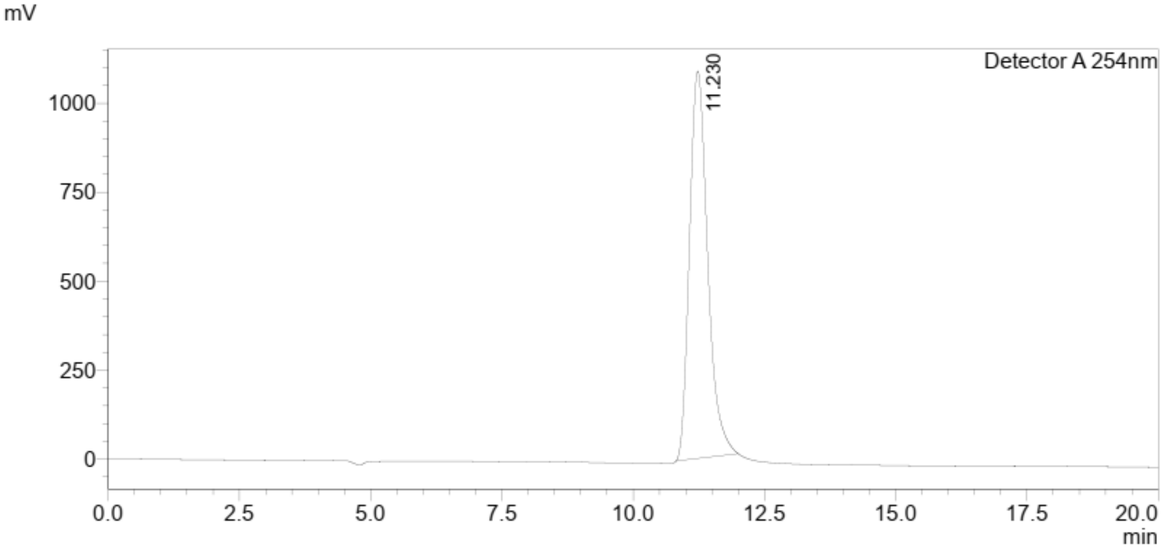

<Peak Table>

Detector A 254nm

Peak#	Ret. Time	Area	Height	Area%	Area/Height	Width at 5% Height
1	11.230	26194559	1088238	100.000	24.071	0.849
Total		26194559	1088238	100.000		

Figure **S24**. HPLC trace report of **DMAC-PA**

Table **S1**. Natural transition orbitals, unoccupied (hole) (blue) & occupied (electron) (red) NTOs (isovalue: 0.02) of compounds.

Compound	S_1	T_1	T_2
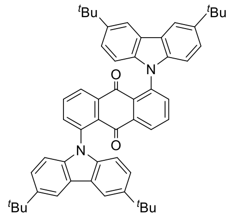	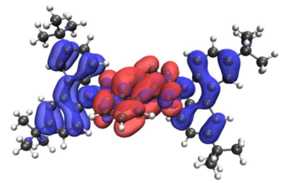	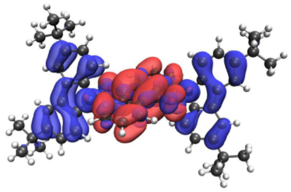	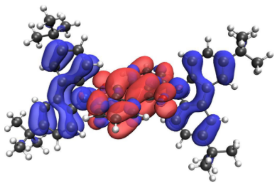
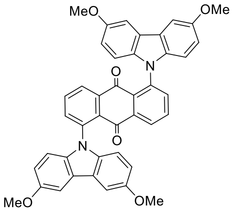	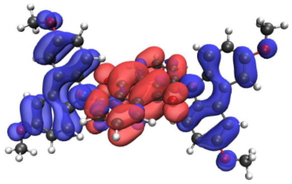	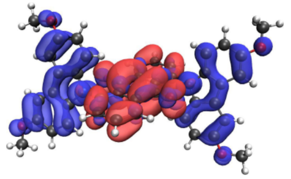	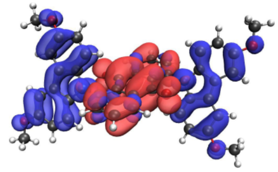
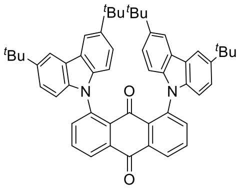	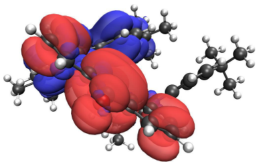	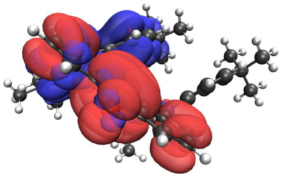	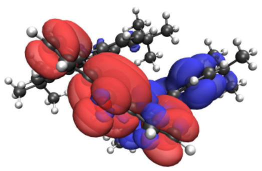
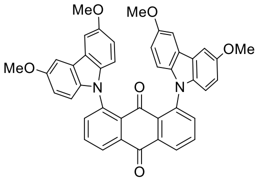	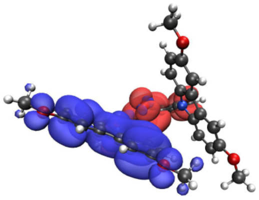	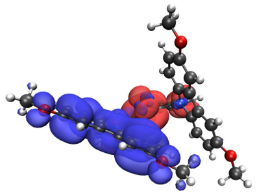	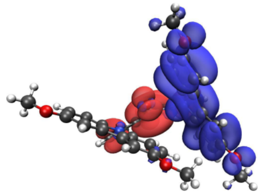
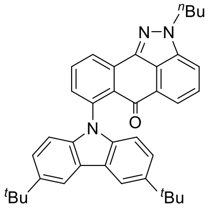	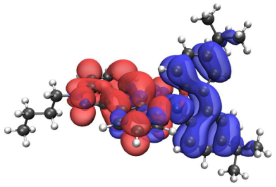	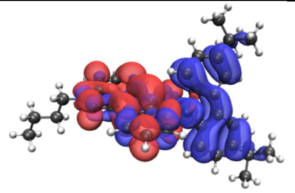	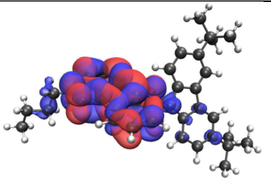
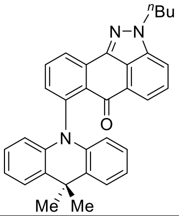	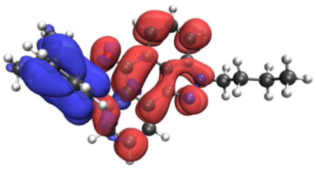	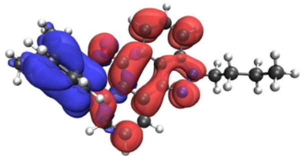	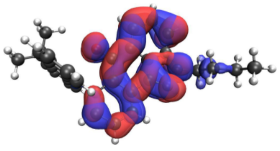

Table **S2**. Excited state energies of S_1, T_1 and T_2 and spin-orbit coupling constants calculated at optimized S_1 geometry using PBE0/6-31G(d,p) level of theory.

Compounds	S_1 / eV	T_1 / eV	T_2 / eV	$<S_1\|\hat{H}_{SOC}\|T_1>$ / cm^{-1}	$<S_1\|\hat{H}_{SOC}\|T_2>$ / cm^{-1}
**T-tBuCz-AQ**	2.05	1.82	1.9312	0.0708	0.0135
**T-MeOCz-AQ**	1.81	1.62	1.6857	0.0231	0.0708
**C-tBuCz-AQ**	1.88	1.79	1.9014	0.0620	0.1601
**C-MeOCz-AQ**	1.68	1.50	1.6797	0.0016	0.0729
**tBuCz-PA**	2.26	2.11	2.3721	0.1259	6.0447
**DMAC-PA**	1.79	1.77	2.3324	0.0631	4.0490

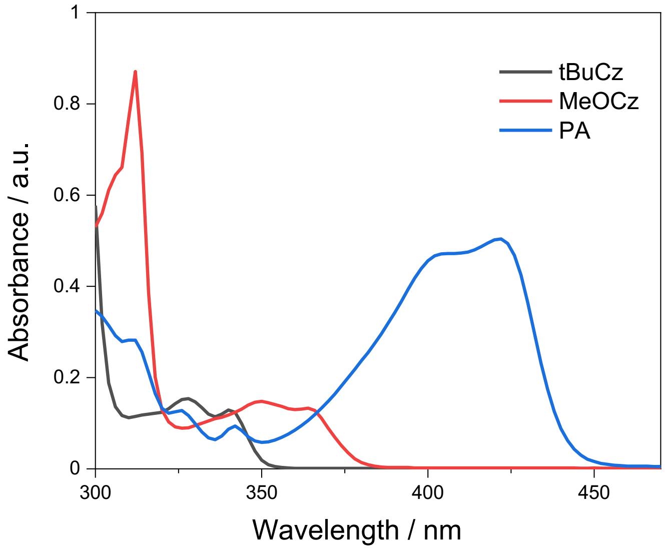

Figure **S25**. Absorption spectra of tBuCz, MeOCz and PA in toluene.

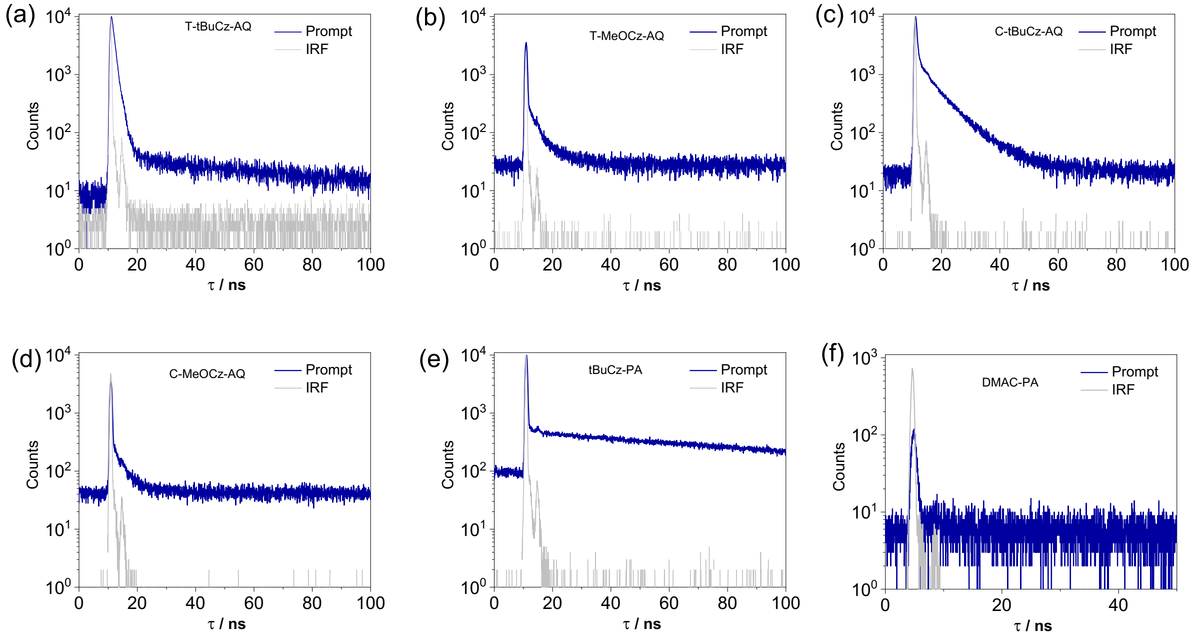

Figure **S26.** TRPL of (a) **T-tBuCz-AQ**, (b) **T-MeOCz-AQ**, (c) **C-tBuCz-AQ**, (d) **C-MeOCz-AQ**, (e) **tBuCz-PA and** (f) **DMAC-PA** (no PL decay observed for this compound).

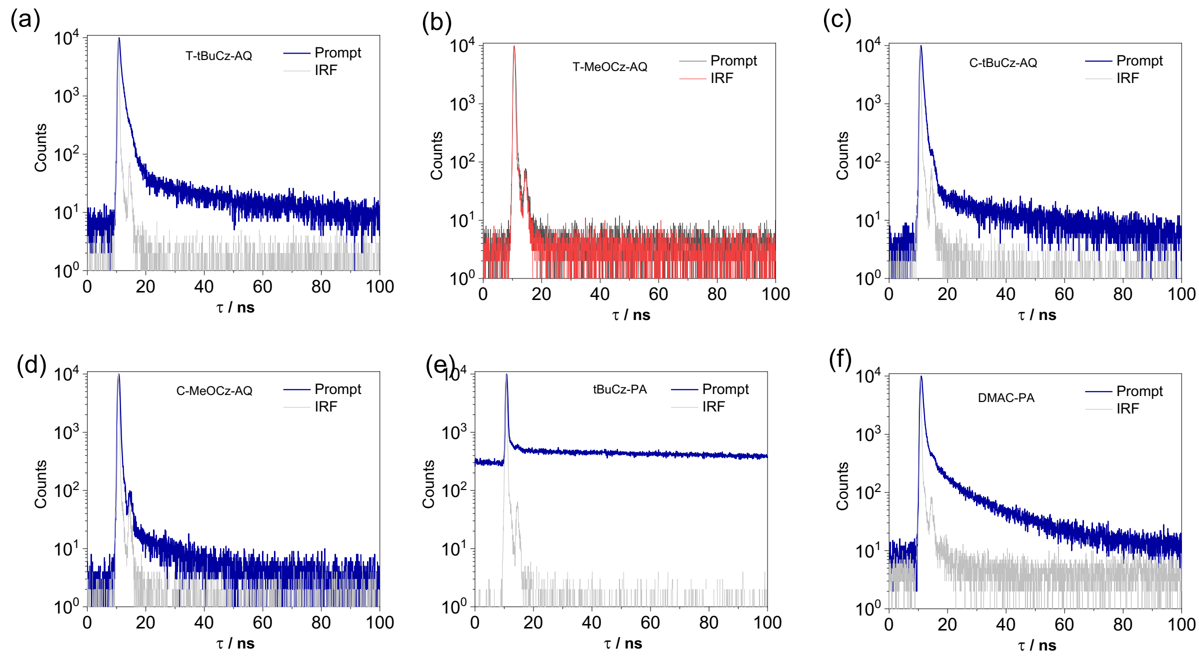

Figure **S27.** TRPL of (a) **T-tBuCz-AQ**, (b) **T-MeOCz-AQ** (no PL decay observed for this compound), (c) **C-tBuCz-AQ**, (d) **C-MeOCz-AQ**, (e) **tBuCz-PA and** (f) **DMAC-PA**.

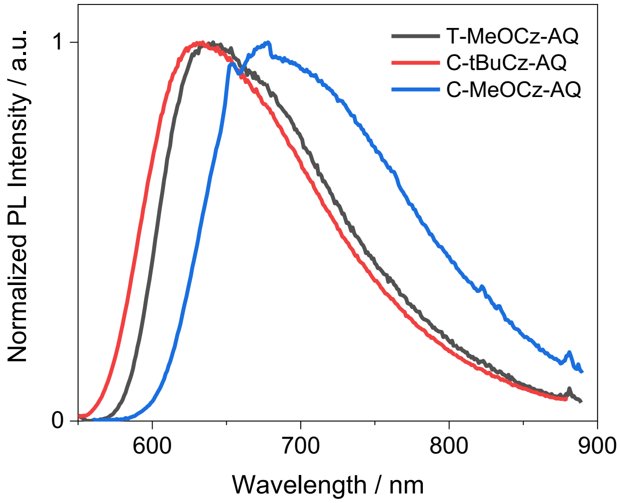

Figure **S28**. Steady-state PL spectra of **T-MeOCz-AQ, C-tBuCz-AQ** and **C-MeOCz-AQ** in 2-Me-THF at 77 K (λ_{exc} = 450 nm).

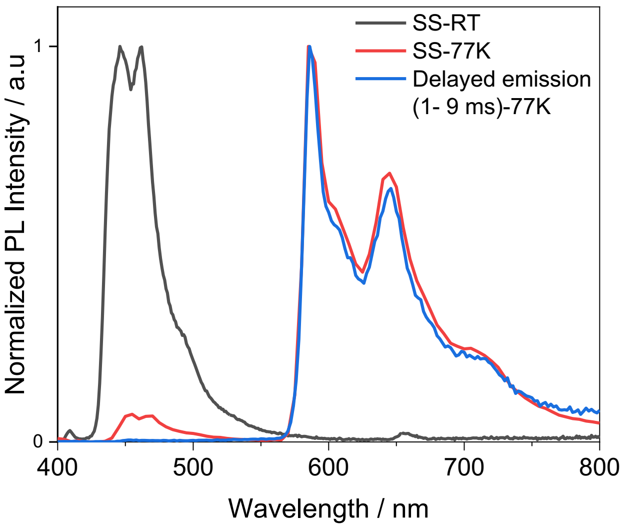

Figure **S29**. Steady-state PL spectra at RT and 77 K, and delayed emission spectra at 77 K of **PA** precursor (λ_{exc} = 450 nm).

Table **S3**. Rate constants of anthrone based TADF compounds.

Compound	Φ_{PL} (O_2 / N_2)^a / %	$\tau_p{}^b$/ ns	$\tau_d{}^c$ / µs	$k_{ISC}{}^d$ / × 10^7 s^{-1}	$k_{RISC}{}^e$ / × 10^6 s^{-1}
**T-tBuCz-AQ**	3.9/ 6.6	3.5	1.00	17.8	2.7
**T-MeOCz-AQ**	2.4/ 2.7	14	0.132	2.9	16.0
**C-tBuCz-AQ**	3.6/ 3.6	88	0.704	0.4	2.8

[a] Φ_{PL} was recorded under air/ N_2 atmosphere using an integrating sphere (λ_{exc} = 450 nm), [b]prompt PL lifetime (τ_p) was recorded using time correlated single photon counting (TCSPC) (λ_{exc} = 375 nm) and, [c]delayed PL lifetime (τ_d) was recorded using TCSPC for **T-tBuCz-AQ, T-MeOCz-AQ and C-tBuCz-AQ** and microsecond flash lamp for **tBuCz-PA** (λ_{exc} = 450 nm), [d] k_{ISC} = intersystem crossing rate constant from S_1 to T_1 states, [e] k_{RISC} = reverse intersystem crossing rate constant.

$$k_p = 1/\tau_p$$

$$k_d = 1/\tau_d$$

$$k_{ISC} = k_p \frac{\Phi_d}{\Phi_{PL}} - k_d \frac{\Phi_d}{\Phi_p}$$

$$k_{RISC} = k_p \frac{\Phi_{PL}}{\Phi_p}$$

k_p and k_d are the rate constants of prompt fluorescence and delayed fluorescence, respectively, k_{ISC} = intersystem crossing rate constant, k_{RISC} = reverse intersystem crossing rate constant, Φ_P and Φ_d are the prompt fluorescence and delayed PL quantum yields. Rate constants are calculated based on the methodology described by Tsuchiya *et al*. (Tsuchiya et al., 2021).

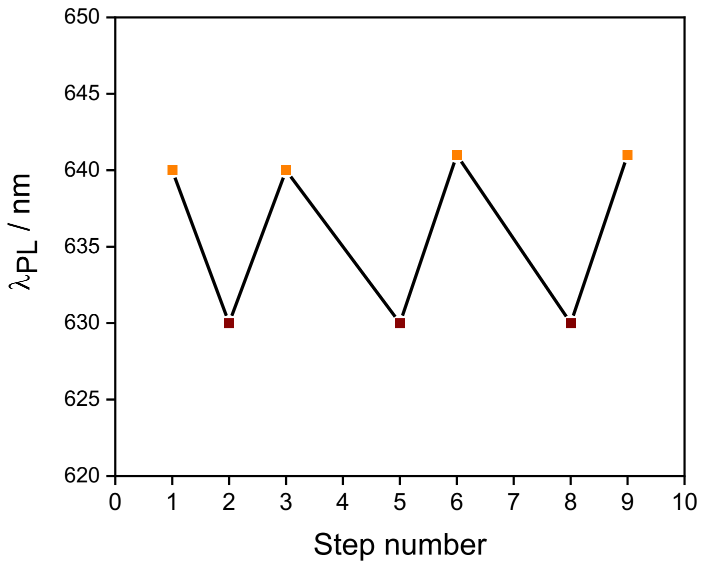

Figure **S30**. Repeated switching of the λ_{PL} upon mechanical pressure and hexane fuming of **tBuCz-PA**.

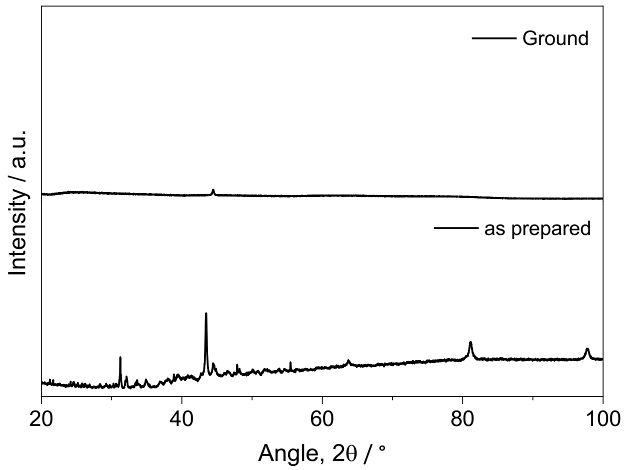

Figure **S31**. PXRD spectra of as prepared and ground of **tBuCz-PA**.

References

Adamo, C., and Barone, V. (1999). Toward reliable density functional methods without adjustable parameters: The PBE0 model. *J. Chem. Phys.* 110**,** 6158-6170.

Allouche, A.-R. (2011). Gabedit—A graphical user interface for computational chemistry softwares. *J. Comput. Chem* 32**,** 174-182.

Dennington, R., Keith, T.A., and Millam, J.M. (2016). *Semichem Inc.: Shawnee Mission, KS, USA*.

Edward, J., Ercal, F., Walters, F.G., and Pottinger, H.J. (1998). AN EFFICIENT LIBRARY FOR PARALLEL RAY TRACING AND ANIMATION, University of Missouri-Rolla.

Frisch, M.J., Trucks, G.W., Schlegel, H.B., Scuseria, G.E., Robb, M.A., Cheeseman, J.R., Scalmani, G., Barone, V., Petersson, G.A., Nakatsuji, H., Li, X., Caricato, M., Marenich, A.V., Bloino, J., Janesko, B.G., Gomperts, R., Mennucci, B., Hratchian, H.P., Ortiz, J.V., Izmaylov, A.F., Sonnenberg, J.L., Williams, Ding, F., Lipparini, F., Egidi, F., Goings, J., Peng, B., Petrone, A., Henderson, T., Ranasinghe, D., Zakrzewski, V.G., Gao, J., Rega, N., Zheng, G., Liang, W., Hada, M., Ehara, M., Toyota, K., Fukuda, R., Hasegawa, J., Ishida, M., Nakajima, T., Honda, Y., Kitao, O., Nakai, H., Vreven, T., Throssell, K., Montgomery Jr., J.A., Peralta, J.E., Ogliaro, F., Bearpark, M.J., Heyd, J.J., Brothers, E.N., Kudin, K.N., Staroverov, V.N., Keith, T.A., Kobayashi, R., Normand, J., Raghavachari, K., Rendell, A.P., Burant, J.C., Iyengar, S.S., Tomasi, J., Cossi, M., Millam, J.M., Klene, M., Adamo, C., Cammi, R., Ochterski, J.W., Martin, R.L., Morokuma, K., Farkas, O., Foresman, J.B., and Fox, D.J. (2016). "Gaussian 16 Rev. C.01". (Wallingford, CT).

Hirata, S., and Head-Gordon, M. (1999). Time-dependent density functional theory within the Tamm–Dancoff approximation. *Chem. Phys. Lett* 314**,** 291-299.

Humphrey, W., Dalke, A., and Schulten, K. (1996). VMD: Visual molecular dynamics. *J. Mol. Graph.* 14**,** 33-38.

Pavlishchuk, V.V., and Addison, A.W. (2000). Conversion constants for redox potentials measured versus different reference electrodes in acetonitrile solutions at 25°C. *Inorg. Chim. Acta* 298**,** 97-102.

Petersson, G.A., Tensfeldt, T.G., and Montgomery, J.A., Jr. (1991). A complete basis set model chemistry. III. The complete basis set-quadratic configuration interaction family of methods. *J. Chem. Phys.* 94**,** 6091-6101.

Tsuchiya, Y., Diesing, S., Bencheikh, F., Wada, Y., Dos Santos, P.L., Kaji, H., Zysman-Colman, E., Samuel, I.D.W., and Adachi, C. (2021). Exact Solution of Kinetic Analysis for Thermally Activated Delayed Fluorescence Materials. *The Journal of Physical Chemistry A* 125**,** 8074-8089.
