# Supplementary material for: Thermally activated delayed fluorescence and high-contrast mechanochromism of anthrone-based donor–acceptor systems
Source: Front Chem. 2023 Aug 31;11:1248267. doi: 10.3389/fchem.2023.1248267 (PMC10501131; doi:10.3389/fchem.2023.1248267)
Supplement: Supplementary file 2 [file DataSheet1.PDF]

Ground state

T-tBuCz-AQ

C	-2.43727400	-1.49831700	-2.44637500
C	-2.48489100	-0.72847200	-1.28125900
C	-1.28391200	-0.35172600	-0.64308800
C	-0.06282100	-0.70282800	-1.24939900
C	-0.03509400	-1.45491600	-2.42071900
C	-1.22295500	-1.87207700	-3.00560500
C	-1.26238900	0.30070400	0.68851000
C	1.26249600	-0.30034100	-0.68832200
C	1.28402500	0.35195500	0.64333900
C	0.06294100	0.70306400	1.24965400
C	0.03524000	1.45503800	2.42105000
C	1.22311500	1.87204500	3.00601700
C	2.43742500	1.49825700	2.44678200
C	2.48501300	0.72855600	1.28157100
O	2.27274500	-0.49477700	-1.34264900
O	-2.27265400	0.49533000	1.34275700
C	5.62640100	-3.34702200	0.28929400
C	4.27680100	-3.33813000	0.69948500
C	3.55715800	-2.16991700	0.89484800
C	4.21054100	-0.96280600	0.67027200
C	5.56114400	-0.93043200	0.27398500
C	6.25900100	-2.12446900	0.08367500
C	4.79397200	1.21396700	0.51876500
C	4.81583300	2.60543500	0.49650900
C	6.00442600	3.22473200	0.13849600
C	7.16862900	2.50744300	-0.20539300
C	7.11346300	1.11603300	-0.18770100
C	5.93371600	0.46223200	0.17046900
N	3.75015700	0.34170700	0.81535500
H	-1.20673400	-2.46334900	-3.91647900
H	0.93180800	-1.69700500	-2.84866300
H	-3.37561400	-1.77051600	-2.91997600
H	-0.93165700	1.69715600	2.84899100
H	1.20691000	2.46321500	3.91695600
H	3.37577200	1.77033000	2.92044100
H	2.51980700	-2.20304300	1.21372900
H	3.76937800	-4.28368700	0.87026000
H	7.29922800	-2.08320700	-0.22217200
H	7.98237800	0.52409600	-0.45577900
H	6.02710500	4.31095900	0.11997800
H	3.93441200	3.19099700	0.73982200
N	-3.75006300	-0.34165800	-0.81508700
C	-4.79381200	-1.21396600	-0.51840600
C	-4.21060200	0.96283800	-0.67031900
C	-4.81553100	-2.60543200	-0.49587900
C	-5.93365600	-0.46228100	-0.17033100
C	-3.55734000	2.16997200	-0.89512200
C	-5.56122500	0.93040100	-0.27411000
C	-6.00408400	-3.22478200	-0.13782100
H	-3.93403500	-3.19095000	-0.73902200
C	-7.11335800	-1.11613300	0.18789300

C	-4.27712500	3.33814700	-0.70006300
H	-2.51997400	2.20314300	-1.21394800
C	-6.25922600	2.12440300	-0.08410200
C	-7.16838200	-2.50754600	0.20585300
H	-6.02665200	-4.31100800	-0.11909400
H	-7.98235100	-0.52423300	0.45579700
C	-5.62674800	3.34698100	-0.28994800
H	-3.76979800	4.28372400	-0.87101300
H	-7.29946600	2.08308700	0.22169000
C	8.43343200	3.27659600	-0.58716400
C	6.33631900	-4.68587000	0.08933500
C	-8.43313100	-3.27675500	0.58769200
C	-6.33681800	4.68580300	-0.09034900
C	7.79001300	-4.50837600	-0.35211800
H	7.86120600	-3.97518400	-1.30681800
H	8.37620700	-3.96018800	0.39408800
H	8.25723800	-5.49048100	-0.48468700
C	6.32965500	-5.47258600	1.40852200
H	5.31122500	-5.66986200	1.75883100
H	6.83196500	-6.43907800	1.27977800
H	6.85184900	-4.91658100	2.19489600
C	5.60371500	-5.49500300	-0.99149600
H	4.56306700	-5.69282500	-0.71441200
H	5.59879300	-4.95425500	-1.94418200
H	6.09847900	-6.46153400	-1.14674700
C	9.59531000	2.34312000	-0.93126800
H	9.86681700	1.70417700	-0.08335000
H	9.35909300	1.70006400	-1.78654400
H	10.47779900	2.93600900	-1.19595000
C	8.14745500	4.15625500	-1.81347400
H	7.34967600	4.88000100	-1.61713400
H	9.04547700	4.71762900	-2.09875800
H	7.83972800	3.54328900	-2.66763000
C	8.86604200	4.16510100	0.58874100
H	9.77076000	4.72838700	0.32962800
H	8.08888100	4.88755700	0.85893100
H	9.08176100	3.55849500	1.47516800
C	-7.79048400	4.50829100	0.35119300
H	-8.37661100	3.95972400	-0.39478500
H	-7.86159900	3.97547800	1.30611200
H	-8.25784200	5.49038800	0.48336800
C	-5.60427100	5.49533200	0.99021600
H	-5.59923300	4.95485900	1.94305900
H	-4.56365900	5.69321200	0.71303300
H	-6.09915300	6.46184000	1.14521500
C	-6.33027300	5.47213500	-1.40977000
H	-6.85245200	4.91584800	-2.19595300
H	-6.83265800	6.43862500	-1.28129000
H	-5.31187200	5.66938800	-1.76017500
C	-9.59512900	-2.34333500	0.93154000
H	-9.35903300	-1.70008200	1.78670200
H	-9.86665400	-1.70459000	0.08347700
H	-10.47757200	-2.93626500	1.19628600
C	-8.14714300	-4.15614700	1.81419000

H	-9.04512500	-4.71755700	2.09952600
H	-7.34927700	-4.87985000	1.61804200
H	-7.83953200	-3.54298400	2.66824700
C	-8.86557100	-4.16553400	-0.58806800
H	-8.08831600	-4.88795900	-0.85806800
H	-9.77024700	-4.72886500	-0.32890400
H	-9.08129700	-3.55912200	-1.47462600

C-tBuCz-AQ

C	3.42542300	3.56548600	2.01448700
C	3.51040600	4.93335700	1.77783900
C	2.63601700	5.53469100	0.88395600
C	1.68468400	4.76212800	0.22058900
C	1.60145300	3.37544700	0.42932600
C	2.48615900	2.77639600	1.34869600
C	0.77741700	5.44581700	-0.73635500
C	-0.31377200	4.64639100	-1.35240500
C	-0.40052900	3.25706500	-1.14756900
C	0.69040100	2.55760000	-0.41994200
C	-1.27546800	5.33135000	-2.09029100
C	-2.35992000	4.64234700	-2.61358700
C	-2.48403600	3.27839900	-2.38247800
C	-1.52025100	2.56806700	-1.65926500
O	0.85916500	1.36021700	-0.54696300
O	0.89675300	6.63704100	-0.97535000
N	-1.80320600	1.20976800	-1.39727700
N	2.48492800	1.39600600	1.61799600
C	3.47988100	0.52961800	1.15896500
C	3.02808500	-0.79999900	1.29326800
C	1.68983200	-0.73233900	1.83653000
C	1.38380900	0.63372700	2.00036000
C	-2.96899100	0.81830800	-0.73838700
C	-3.10930900	-0.58258000	-0.83982200
C	-1.96752000	-1.05828100	-1.58729600
C	-1.20982700	0.07195200	-1.94692600
C	0.74452700	-1.69565600	2.16893400
C	-0.51124300	-1.31863900	2.64712000
C	-0.78586800	0.05291000	2.78591600
C	0.14600900	1.03845600	2.47829400
C	4.72373700	0.81222000	0.61359200
C	5.51875400	-0.26021100	0.21712000
C	5.10763000	-1.59723300	0.34468700
C	3.84596400	-1.84959400	0.88973800
C	-1.53221200	-2.33564700	-1.94720500
C	-0.35973200	-2.49257900	-2.67736000
C	0.32923900	-1.33149000	-3.08712400
C	-0.07745700	-0.05300700	-2.74448400
C	-3.90138600	1.58246400	-0.04802100
C	-5.00799300	0.93160600	0.49066400
C	-5.20862100	-0.45413600	0.36571700
C	-4.22872100	-1.20336600	-0.29195600
C	5.98255800	-2.77102700	-0.09893200
C	6.26255500	-3.68231800	1.10527100
C	5.24841200	-3.57310700	-1.18384800

C	7.32497500	-2.31334700	-0.67205100
C	-1.53181600	-2.39915500	3.00320200
C	-2.85459700	-1.80408300	3.48567400
C	-1.81691800	-3.25988500	1.76361300
C	-0.96520100	-3.28515600	4.12219500
C	0.22997700	-3.86382900	-3.00139200
C	-6.48178800	-1.14518400	0.86078900
C	1.59732600	-3.98170000	-2.30895300
C	-0.65714400	-5.00576100	-2.50309100
C	0.40675300	-4.01988300	-4.51823100
C	-7.41855000	-0.18318400	1.59391200
C	-7.23273000	-1.71420300	-0.35349700
C	-6.13553200	-2.29062100	1.82146400
H	4.08146800	3.08067000	2.73119300
H	4.25057300	5.52812700	2.30487600
H	2.66692900	6.59939500	0.67732400
H	-1.15087700	6.40040600	-2.22770100
H	-3.11439300	5.16325700	-3.19558600
H	-3.34071800	2.72916400	-2.76111900
H	0.98968300	-2.74712300	2.04144800
H	-1.76052600	0.37063700	3.13869900
H	-0.10123400	2.08996000	2.59505900
H	5.06860000	1.83378800	0.48416900
H	6.48993200	-0.03728500	-0.21101700
H	3.48594000	-2.87066500	0.98948200
H	-2.11088800	-3.19761700	-1.63207700
H	1.23641400	-1.43482500	-3.67605200
H	0.49542900	0.81440900	-3.05147400
H	-3.78525700	2.65661000	0.06036300
H	-5.74260300	1.53403200	1.01356900
H	-4.34548000	-2.27845500	-0.40345400
H	6.88339300	-4.53505800	0.80434000
H	6.79079300	-3.13418900	1.89317900
H	5.33714500	-4.07768700	1.53667400
H	5.86094500	-4.42097500	-1.51428700
H	5.03525200	-2.94360400	-2.05490800
H	4.29526000	-3.97010900	-0.82023700
H	7.19400400	-1.68234200	-1.55839500
H	7.91240600	-1.75493400	0.06558800
H	7.91384300	-3.18734600	-0.97181800
H	-3.55237800	-2.61333600	3.72770900
H	-2.72099200	-1.20057700	4.39088700
H	-3.32086800	-1.18103800	2.71459600
H	-2.55820100	-4.03411100	1.99872600
H	-0.91286200	-3.75989900	1.39961100
H	-2.20476600	-2.63914000	0.95119800
H	-0.75824800	-2.69314900	5.02065300
H	-1.68219800	-4.07206800	4.38692500
H	-0.03126100	-3.77040300	3.81963600
H	2.04921800	-4.96113000	-2.50984600
H	2.29421800	-3.21139200	-2.65415200
H	1.49044400	-3.86397600	-1.22442100
H	-0.19661000	-5.96762800	-2.75464300
H	-1.64960800	-4.98019900	-2.96751300

H	-0.78505600	-4.97330700	-1.41499700
H	-0.55798800	-3.94266300	-5.03176500
H	1.06878500	-3.25269000	-4.93256000
H	0.84324400	-4.99820400	-4.75331900
H	-8.30419000	-0.72650600	1.94153100
H	-6.93573200	0.26150500	2.47156800
H	-7.76349600	0.62677400	0.94147800
H	-8.16047000	-2.20532200	-0.03517000
H	-6.62754100	-2.45264600	-0.88972300
H	-7.49048700	-0.91733800	-1.05979000
H	-5.65963900	-1.90722400	2.72911900
H	-7.04621200	-2.82735700	2.11360300
H	-5.45161900	-3.01529500	1.36759800

T-MeOCz-AQ

C	-2.54612400	-1.62070000	-2.24788700
C	-2.54161700	-0.75577200	-1.14910200
C	-1.31179400	-0.36082900	-0.57805900
C	-0.11803700	-0.79257300	-1.18835800
C	-0.14363900	-1.63764600	-2.29388900
C	-1.35896100	-2.06959100	-2.80852700
C	-1.23291000	0.38941900	0.69721400
C	1.23357100	-0.38571200	-0.69743500
C	1.31251400	0.36325900	0.57859700
C	0.11880700	0.79521300	1.18881000
C	0.14459500	1.63952200	2.29494000
C	1.36001400	2.07021100	2.81037900
C	2.54710500	1.62087500	2.24990600
C	2.54239200	0.75690100	1.15039800
O	2.21863100	-0.65912500	-1.36302800
O	-2.21814100	0.66451500	1.36187900
C	5.52110900	-3.45546100	0.38852300
C	4.17864900	-3.40042300	0.80856700
C	3.50822700	-2.19671500	0.92773800
C	4.20039200	-1.02771800	0.61844900
C	5.54685600	-1.06720900	0.21310700
C	6.21638800	-2.28886700	0.09265100
C	4.85387000	1.11636800	0.32995400
C	4.92197900	2.49775100	0.21558000
C	6.12512200	3.07069300	-0.18984900
C	7.24046200	2.27157000	-0.48666100
C	7.16200500	0.88358600	-0.38695300
C	5.96750100	0.30142900	0.02056100
N	3.78160000	0.29903200	0.68516000
H	-1.38455300	-2.73196600	-3.66892600
H	0.80371400	-1.93937800	-2.72753600
H	-3.50392800	-1.90474300	-2.67300300
H	-0.80271300	1.94161100	2.72843100
H	1.38572800	2.73191600	3.67129100
H	3.50493500	1.90383100	2.67569100
H	2.47417000	-2.17278800	1.25668300
H	3.68063700	-4.33772300	1.03508000
H	7.25465500	-2.30743500	-0.21938600
H	8.03536800	0.28911500	-0.63682000

H	6.18239800	4.14942300	-0.27934700
H	4.06164700	3.12600100	0.42441400
N	-3.78103800	-0.29852600	-0.68379900
C	-4.85265000	-1.11653900	-0.32811100
C	-4.20127100	1.02787300	-0.61897800
C	-4.91937600	-2.49784800	-0.21207900
C	-5.96722600	-0.30237100	-0.02008800
C	-3.51024100	2.19718300	-0.92959000
C	-5.54791700	1.06647000	-0.21416500
C	-6.12208000	-3.07154400	0.19360000
H	-4.05832700	-3.12546800	-0.41985400
C	-7.16125300	-0.88525400	0.38775500
C	-4.18197100	3.40034200	-0.81220600
H	-2.47607300	2.17389800	-1.25821500
C	-6.21876300	2.28757600	-0.09548600
C	-7.23832900	-2.27321300	0.48907300
H	-6.17827400	-4.15022600	0.28441800
H	-8.03530200	-0.29138400	0.63665000
C	-5.52460600	3.45452000	-0.39262300
H	-3.68488200	4.33786800	-1.03980500
H	-7.25719200	2.30545800	0.21606100
O	-8.44228300	-2.76817600	0.88938600
O	-6.05239000	4.70648300	-0.31669000
O	6.04752500	-4.70789100	0.31085400
O	8.44480100	2.76576000	-0.88686500
C	7.38396500	-4.81859300	-0.10888100
H	7.61422000	-5.88546200	-0.11364800
H	7.52521800	-4.41336400	-1.12028800
H	8.06872000	-4.30453500	0.57972800
C	8.56750800	4.15883600	-1.02201900
H	9.59211000	4.34190300	-1.35074700
H	7.87227800	4.55783000	-1.77337700
H	8.39846500	4.67721500	-0.06805500
C	-7.38911600	4.81625400	0.10239100
H	-7.53037500	4.41192100	1.11415400
H	-7.62045800	5.88289500	0.10599200
H	-8.07307000	4.30080300	-0.58596900
C	-8.56334200	-4.16116800	1.02680500
H	-7.86770300	-4.55809400	1.77887400
H	-9.58777300	-4.34492600	1.35568100
H	-8.39358600	-4.68090200	0.07370600

C-MeOCz-AQ

C	3.55319400	2.50272600	1.55851200
C	3.62513900	3.88698200	1.59862500
C	2.61434400	4.64991300	1.02747800
C	1.53636800	4.01601500	0.41906400
C	1.45917200	2.61042800	0.34328500
C	2.48880500	1.84435300	0.92678100
C	0.47621500	4.87852900	-0.16522000
C	-0.76290000	4.21395300	-0.64042800
C	-0.83720300	2.81398100	-0.71846200
C	0.38409100	1.99386000	-0.47895200
C	-1.86617800	5.01226100	-0.93534800

C	-3.07537500	4.42083300	-1.27155300
C	-3.17880400	3.03405300	-1.29522300
C	-2.07349500	2.22229100	-1.03172000
O	0.50037400	0.88532000	-0.96588700
O	0.59300900	6.09337100	-0.19773100
N	-2.28724700	0.82865100	-0.97629000
N	2.50836500	0.44649300	0.92458700
C	3.62299500	-0.31141900	0.54962600
C	3.34921900	-1.67761400	0.77696700
C	2.01097500	-1.74971400	1.31793000
C	1.51635500	-0.42818000	1.38166000
C	-3.04473900	0.23265100	0.02451800
C	-3.28383900	-1.11918100	-0.31878900
C	-2.65770800	-1.33542300	-1.60392800
C	-2.06361900	-0.11018200	-1.98058800
C	1.23912900	-2.81268100	1.76654400
C	-0.03291200	-2.55899700	2.27430900
C	-0.52791800	-1.24583100	2.31988700
C	0.24533100	-0.17437500	1.87938100
C	4.82413900	0.08884300	-0.01945600
C	5.76995200	-0.88577200	-0.33151100
C	5.51328900	-2.24343400	-0.08895900
C	4.29598900	-2.64309000	0.46052000
C	-2.56678200	-2.44014300	-2.44451400
C	-1.89404900	-2.31032900	-3.65641000
C	-1.31847000	-1.08233900	-4.02440400
C	-1.39776200	0.02643400	-3.18848300
C	-3.51773700	0.77323100	1.21189700
C	-4.24651400	-0.04996200	2.06931300
C	-4.49288600	-1.39107400	1.73758300
C	-4.01056500	-1.92985400	0.54437200
H	4.31694500	1.89740800	2.03655000
H	4.46102000	4.36871100	2.09750600
H	2.63131200	5.73450700	1.04342900
H	-1.74936400	6.08956500	-0.87595700
H	-3.94253300	5.03343500	-1.49974500
H	-4.12489600	2.54914300	-1.51705100
H	1.60291900	-3.83507300	1.74216900
H	-1.53215300	-1.04662200	2.67710100
H	-0.15873900	0.83167900	1.93081400
H	5.03073500	1.13221400	-0.23533200
H	6.70853400	-0.57388800	-0.77519100
H	4.11082000	-3.70084600	0.61909300
H	-3.00395000	-3.39875800	-2.18343200
H	-0.79206900	-0.98502800	-4.96700000
H	-0.93058100	0.96426300	-3.46912900
H	-3.33167000	1.81065900	1.47327600
H	-4.62220900	0.36590200	2.99727100
H	-4.21303800	-2.97224900	0.31800600
O	6.39232400	-3.24560100	-0.36654900
O	-0.71442400	-3.65087000	2.71097500
O	-1.84649400	-3.43226900	-4.42828500
O	-5.18195800	-2.25741500	2.53603100
C	-5.65116500	-1.77515600	3.76952400

H	-6.15389300	-2.61531000	4.25182500
H	-4.82773100	-1.43122700	4.41120400
H	-6.36883100	-0.95367100	3.63972800
C	-1.13844500	-3.36218000	-5.63931500
H	-1.20265800	-4.35648200	-6.08525400
H	-1.58135700	-2.63076100	-6.32971600
H	-0.08194500	-3.10727000	-5.47874900
C	-1.96269900	-3.44627400	3.32745300
H	-2.29688400	-4.42975800	3.66347300
H	-1.87793300	-2.77850100	4.19662800
H	-2.69941900	-3.03576500	2.62984000
C	7.62279800	-2.89318900	-0.94536700
H	8.16844900	-3.82753500	-1.08910200
H	7.48888400	-2.40156600	-1.91880900
H	8.20735300	-2.23313600	-0.28955800

tBuCz-PA

C	-5.10709300	1.59545500	-1.94304000
C	-4.89349300	0.88030800	-0.75717000
C	-3.59010800	0.51958700	-0.41802300
C	-2.46307200	0.80655600	-1.16895800
C	-2.66918400	1.51093000	-2.34719100
C	-3.97894400	1.89366900	-2.70703300
C	-3.66584200	-0.18953900	0.78660800
C	-1.17359000	0.29719900	-0.64032400
C	-1.21599800	-0.37567400	0.71944600
C	-2.44144300	-0.68272000	1.38652600
C	-2.45850400	-1.43470500	2.56047900
H	-3.41547400	-1.64778700	3.02678700
C	-1.27199700	-1.89538500	3.10971200
C	-0.06259900	-1.57050600	2.50931600
C	-0.02396700	-0.80928700	1.33981900
H	-6.09461000	1.90606200	-2.27087300
H	-4.11873600	2.44655500	-3.63170800
H	-1.28303400	-2.48254600	4.02347500
H	0.88043500	-1.87582600	2.95200300
N	-4.92748900	-0.27628800	1.18996900
N	-5.66503700	0.38532700	0.26633900
C	-7.10615900	0.35733300	0.35516000
H	-7.48462000	1.27752600	-0.10485300
H	-7.36059600	0.38904800	1.41845100
C	-7.71295500	-0.87237700	-0.31045500
H	-7.41187400	-0.89575700	-1.36653100
H	-7.28391400	-1.76673900	0.15872000
O	-0.15055900	0.39535800	-1.29584500
C	4.45885500	-2.80229300	-0.46830000
C	3.27663200	-3.46077200	-0.08945400
C	2.14998000	-2.78174300	0.36469600
C	2.21579700	-1.39692900	0.44550300
C	3.38374000	-0.69906600	0.06524600
C	4.49217100	-1.40791100	-0.38565400
H	3.22287900	-4.54217500	-0.15222700
H	1.24822900	-3.32144300	0.63758500
H	5.38642700	-0.86315100	-0.67868300

C	1.78070100	0.79921100	0.73170100
C	1.21405800	2.02798700	1.03980700
C	1.98899100	3.16711600	0.85211500
C	3.30880300	3.11695500	0.37050000
C	3.85588200	1.86599500	0.07764200
C	3.10564800	0.70763100	0.25422800
H	0.19690400	2.10394100	1.41228400
H	1.54222500	4.12643300	1.08943800
H	4.87595100	1.78630400	-0.29045500
N	1.25231800	-0.47992300	0.84669500
H	-1.82537000	1.76059800	-2.98268500
C	4.15373100	4.37493500	0.16078100
C	5.69329500	-3.55500400	-0.96832600
C	4.56582800	4.47171900	-1.31567100
H	5.17742300	5.36648100	-1.48546300
H	5.15094400	3.60127300	-1.62985400
H	3.68313500	4.53034800	-1.96178800
C	5.41271000	4.29738600	1.03706200
H	6.03510200	5.18941300	0.89443500
H	5.14448500	4.23170800	2.09726800
H	6.02218400	3.42132100	0.79235500
C	3.39740700	5.65313200	0.52762400
H	2.49825300	5.78249500	-0.08528500
H	3.09998600	5.66066300	1.58236100
H	4.04201900	6.52302500	0.35882400
C	5.48179300	-5.06988600	-0.99408600
H	5.26478500	-5.46666100	0.00416800
H	4.66246800	-5.35405000	-1.66401800
H	6.39151800	-5.56196500	-1.35590700
C	6.88323600	-3.25497500	-0.04467400
H	7.78086000	-3.78111300	-0.39224400
H	7.11266100	-2.18475200	-0.01565500
H	6.67080000	-3.57864700	0.98024000
C	6.02798800	-3.09556200	-2.39523900
H	5.19374700	-3.30084100	-3.07490200
H	6.23419700	-2.02106300	-2.43617300
H	6.91471600	-3.62189700	-2.76951000
C	-9.23272700	-0.89779900	-0.20134000
H	-9.52127400	-0.86560400	0.85831900
H	-9.64754600	0.01171000	-0.65828100
C	-9.84523400	-2.12604700	-0.86127800
H	-9.47113400	-3.04770700	-0.40108900
H	-10.93635300	-2.12615700	-0.77089900
H	-9.59789100	-2.16523200	-1.92842800

DMAC-PA

C	3.90089900	-1.03814900	-2.44225500
C	3.71083100	-0.82829800	-1.07015700
C	2.41853300	-0.59822500	-0.60014100
C	1.27917500	-0.56590500	-1.38557600
C	1.46154600	-0.77475200	-2.74585600
C	2.76092600	-1.00471000	-3.24530700
C	2.51655500	-0.41933700	0.78442900
C	-0.00138200	-0.30838700	-0.68239600

C	0.07720200	−0.11356800	0.82258800
C	1.30760500	−0.16857600	1.54359200
C	1.34339500	0.01325500	2.92760400
H	2.30393000	−0.03860900	3.43124000
C	0.17562500	0.25277800	3.63202000
C	−1.03676600	0.31145600	2.95129200
C	−1.09256100	0.13265000	1.57223500
H	4.87906300	−1.21762200	−2.87826000
H	2.88221500	−1.16372700	−4.31308900
H	0.20385200	0.39391100	4.70863000
H	−1.96941600	0.49739000	3.47472400
N	3.78135800	−0.52675000	1.17328800
N	4.49849800	−0.78966900	0.05527700
C	5.94016100	−0.83347100	0.12891900
H	6.29160700	−1.51160700	−0.65735600
H	6.19623600	−1.28221000	1.09292600
C	6.57929300	0.54286700	−0.01483600
H	6.27550800	0.98271300	−0.97439000
H	6.17763000	1.19480200	0.77113000
O	−1.04875900	−0.26044600	−1.30529000
H	0.60659000	−0.75933500	−3.41438900
N	−2.37629200	0.20981000	0.96558200
C	−2.76712600	1.42110800	0.39263800
C	−3.06357900	−0.97891200	0.71466600
C	−3.86979800	1.48089800	−0.47291600
C	−2.05891400	2.59311400	0.69956900
C	−4.17564800	−0.99601100	−0.14069600
C	−2.64680200	−2.16501800	1.33817900
C	−4.22073500	2.72453900	−1.00005300
C	−2.43232400	3.81260400	0.15676800
H	−1.20779800	2.54439600	1.36966700
C	−4.83044400	−2.21258500	−0.33784200
C	−3.31806900	−3.35771300	1.11886400
H	−1.78785800	−2.14897800	1.99981400
C	−3.52366600	3.88815600	−0.70104700
H	−5.06426500	2.78184200	−1.68285100
H	−1.86495100	4.70411000	0.41010500
H	−5.68456300	−2.24181300	−1.00917400
C	−4.42261100	−3.39027800	0.27534100
H	−2.97248800	−4.26166000	1.61313600
H	−3.82700000	4.83582600	−1.13591700
H	−4.95740400	−4.31739100	0.09189100
C	−4.65274200	0.24225300	−0.88826400
C	−6.14854000	0.46562100	−0.59993400
H	−6.53039700	1.33636800	−1.14198700
H	−6.74465200	−0.39846800	−0.90948700
H	−6.31389600	0.62935200	0.46956800
C	−4.43634700	0.01318800	−2.39683600
H	−3.37369600	−0.14520100	−2.60030600
H	−4.99299700	−0.86581000	−2.74063200
H	−4.77774100	0.87926900	−2.97480600
C	8.09951100	0.48551200	0.07396200
H	8.39046400	0.03633000	1.03351700
H	8.48641100	−0.18476700	−0.70648300

C	8.74508800	1.85779800	-0.06515000
H	8.39936200	2.53700200	0.72246300
H	9.83602300	1.79493500	0.00264300
H	8.49562400	2.31535500	-1.02947700

Excited S1 state optimization

T-tBuCz-AQ

C	2.16285700	-0.00026300	-3.12999000
C	2.32260700	-0.00016400	-1.75790500
C	1.20384700	-0.00012900	-0.88436700
C	-0.08977300	-0.00018200	-1.47789400
C	-0.22479800	-0.00029200	-2.88021700
C	0.87929300	-0.00033500	-3.69959800
C	1.35825600	-0.00005300	0.57237700
C	-1.32094700	-0.00012000	-0.69944800
C	-1.16700400	-0.00000600	0.74661800
C	0.11089800	0.00002800	1.35555200
C	0.22674300	0.00012800	2.75623000
C	-0.88467800	0.00019800	3.56908400
C	-2.16555100	0.00016700	2.98706000
C	-2.28378200	0.00007100	1.61648700
O	-2.45277000	-0.00014600	-1.24372100
O	2.45153300	-0.00006700	1.15769500
C	-6.20197200	3.06119000	0.06552000
C	-4.92801700	3.40732300	0.55763300
C	-3.98802800	2.45858800	0.92507600
C	-4.35066100	1.11848800	0.79125000
C	-5.61981700	0.72909700	0.30253400
C	-6.53695700	1.69501200	-0.05627400
C	-4.35066200	-1.11848400	0.79133300
C	-3.98800100	-2.45853200	0.92528600
C	-4.92798800	-3.40733900	0.55792300
C	-6.20192900	-3.06128900	0.06578600
C	-6.53693300	-1.69512600	-0.05612800
C	-5.61979800	-0.72916500	0.30259900
N	-3.61552700	0.00004900	1.07620800
H	0.76664500	-0.00041500	-4.78006100
H	-1.23524300	-0.00033900	-3.27600600
H	3.05351800	-0.00027900	-3.75090100
H	1.23524000	0.00014400	3.15858300
H	-0.78481300	0.00027400	4.64999700
H	-3.05996700	0.00022200	3.60354400
H	-3.00581900	2.73515400	1.29080400
H	-4.66341600	4.45549200	0.64809100
H	-7.50968700	1.40354000	-0.43551500
H	-7.50966700	-1.40369500	-0.43539200
H	-4.66334800	-4.45549100	0.64848200
H	-3.00578700	-2.73505900	1.29103400
N	3.65204000	-0.00008100	-1.26297000
C	4.36736000	1.12177300	-0.89288200
C	4.36753200	-1.12183300	-0.89291200
C	4.04037000	2.46696800	-1.03390400
C	5.59039600	0.72194800	-0.31310000

C	4.04074900	-2.46707500	-1.03396700
C	5.59050500	-0.72183500	-0.31311500
C	4.96207400	3.40179900	-0.58906600
H	3.09403700	2.77023400	-1.47176500
C	6.49893000	1.68971300	0.11946700
C	4.96259500	-3.40177600	-0.58915100
H	3.09446300	-2.77047300	-1.47183700
C	6.49918600	-1.68947200	0.11943000
C	6.19872700	3.04317400	-0.01087100
H	4.71283000	4.45488700	-0.69273400
H	7.43685400	1.37063500	0.56272000
C	6.19919200	-3.04297500	-0.01094300
H	4.71351400	-4.45490000	-0.69284500
H	7.43706100	-1.37026300	0.56269300
C	-7.17430900	-4.16780300	-0.32568000
C	-7.17436700	4.16765000	-0.32602900
C	7.15206000	4.14585600	0.45091700
C	7.15269700	-4.14552400	0.45081100
C	-8.50874500	3.62025200	-0.83609200
H	-8.38162700	3.00563400	-1.73429000
H	-9.02459600	3.02447000	-0.07447600
H	-9.16598100	4.45485900	-1.10034900
C	-7.45203900	5.05227200	0.90052400
H	-6.53939600	5.51717900	1.28595100
H	-8.14676300	5.85517800	0.62973800
H	-7.90252100	4.46945400	1.71120300
C	-6.54250300	5.01745600	-1.44104000
H	-5.60590600	5.48360800	-1.12029300
H	-6.32907000	4.40791500	-2.32532700
H	-7.23157100	5.81769400	-1.73352200
C	-8.50867400	-3.62045700	-0.83583400
H	-9.02454300	-3.02459400	-0.07429300
H	-8.38153400	-3.00593400	-1.73409300
H	-9.16590700	-4.45509100	-1.10002000
C	-6.54241400	-5.01771300	-1.44059000
H	-5.60581600	-5.48381600	-1.11977200
H	-7.23146000	-5.81799300	-1.73301200
H	-6.32896500	-4.40825700	-2.32493300
C	-7.45201600	-5.05230400	0.90094800
H	-8.14673200	-5.85524000	0.63022900
H	-6.53938000	-5.51717200	1.28644400
H	-7.90251600	-4.46940700	1.71156000
C	8.44220100	-3.58205400	1.05040200
H	8.99616700	-2.97814500	0.32285900
H	8.24101900	-2.96107500	1.93053300
H	9.09383000	-4.40520200	1.36521200
C	6.46438000	-5.00323500	1.52320000
H	6.18850800	-4.39114800	2.38882300
H	5.55083800	-5.47096800	1.14176400
H	7.13400800	-5.80271400	1.86465000
C	7.53153900	-5.03248900	-0.74459400
H	8.03242500	-4.44286400	-1.52037100
H	8.21102700	-5.83424600	-0.42903000
H	6.65052000	-5.49889900	-1.19750800

C	8.44164500	3.58256800	1.05050500
H	8.24055100	2.96154100	1.93062200
H	8.99570700	2.97875800	0.32295300
H	9.09314800	4.40580800	1.36533800
C	6.46360300	5.00343600	1.52332100
H	7.13310400	5.80301100	1.86479400
H	5.54999100	5.47103500	1.14188900
H	6.18782100	4.39128700	2.38892800
C	7.53077800	5.03290900	-0.74446200
H	6.64969100	5.49919700	-1.19737200
H	8.21014200	5.83476100	-0.42887200
H	8.03175900	4.44337900	-1.52025000

C-tBuCz-AQ

C	-2.50914100	3.55998000	-2.68930500
C	-2.43888400	4.96455500	-2.60992000
C	-1.76716100	5.55136500	-1.56103100
C	-1.12064100	4.77591700	-0.58258000
C	-1.15679700	3.36219800	-0.65330800
C	-1.89717900	2.79828000	-1.72159500
C	-0.42160700	5.46066500	0.50754000
C	0.35741200	4.61200900	1.41104600
C	0.40935500	3.20021500	1.26030700
C	-0.46470000	2.51763300	0.31658800
C	1.10560400	5.24434100	2.41855600
C	1.92386600	4.51594800	3.25264900
C	2.05195400	3.13059500	3.05793700
C	1.32609000	2.48279200	2.07721400
O	-0.63253600	1.27904100	0.32933900
O	-0.46088200	6.69729500	0.62557700
N	1.67681100	1.12763800	1.81052600
N	-2.07260700	1.37318100	-1.77955900
C	-3.12485600	0.71151300	-1.19982100
C	-2.91354400	-0.68474800	-1.30710400
C	-1.62931600	-0.84291400	-1.97808800
C	-1.17026900	0.46624000	-2.26568300
C	2.83081200	0.78615900	1.12527500
C	2.92995700	-0.62334300	1.04997200
C	1.75677900	-1.14661500	1.70879900
C	1.01737500	-0.03455500	2.16471700
C	-0.82378400	-1.91744500	-2.26772600
C	0.44645600	-1.71176100	-2.86147000
C	0.85064900	-0.40227800	-3.16244300
C	0.06204200	0.70289100	-2.86832900
C	-4.25433900	1.24277400	-0.58491200
C	-5.19218300	0.34521600	-0.08735600
C	-5.02618600	-1.04471700	-0.18554700
C	-3.85583200	-1.55110400	-0.80202800
C	1.27568600	-2.44081200	1.92143400
C	0.06763000	-2.63753200	2.58033800
C	-0.63496200	-1.50102400	3.04022700
C	-0.18290600	-0.20699200	2.84835100
C	3.80895500	1.60302200	0.56964600
C	4.90264800	0.99186100	-0.03428100

C	5.05359500	-0.40537900	-0.09961700
C	4.04294500	-1.20289200	0.44380800
C	-6.06745500	-2.02642700	0.33855400
C	-6.58367400	-2.87630800	-0.83466000
C	-5.41901000	-2.94386600	1.38690800
C	-7.26130500	-1.32349900	0.98639600
C	1.34969000	-2.91515400	-3.08806900
C	2.64710100	-2.54015100	-3.80356600
C	1.70561100	-3.50159700	-1.71032000
C	0.61618400	-3.96984500	-3.93069300
C	-0.53720400	-4.02539200	2.78768500
C	6.29675000	-1.06749100	-0.69943000
C	-1.87577800	-4.09960000	2.03791000
C	0.36744800	-5.13940700	2.25874300
C	-0.77783800	-4.27735500	4.28305300
C	7.30422100	-0.04556300	-1.22986200
C	6.99439400	-1.90520300	0.38318400
C	5.89469000	-1.98248400	-1.86405600
H	-3.05523200	3.07163400	-3.49110500
H	-2.92675700	5.57027000	-3.36726600
H	-1.70624100	6.62974500	-1.44891000
H	1.01561300	6.32344000	2.49581800
H	2.49836500	5.00701800	4.03268200
H	2.75340700	2.54889900	3.64888800
H	-1.14191700	-2.92165600	-2.00562500
H	1.82200900	-0.23316200	-3.60949100
H	0.40262400	1.71355600	-3.06267700
H	-4.38964200	2.31498100	-0.49887800
H	-6.07749900	0.74481100	0.39156600
H	-3.70175700	-2.62350500	-0.87275500
H	1.85492500	-3.28425100	1.55905200
H	-1.58149800	-1.63850100	3.55725000
H	-0.75837900	0.64873800	3.18467000
H	3.72874300	2.68449700	0.61854400
H	5.66937200	1.63298300	-0.45577700
H	4.13106700	-2.28715800	0.42187700
H	-7.33020000	-3.59330000	-0.47478500
H	-7.05362200	-2.24697900	-1.59806600
H	-5.77966200	-3.44468500	-1.31340000
H	-6.16324700	-3.64779400	1.77608300
H	-5.02559400	-2.36185500	2.22705100
H	-4.59444500	-3.52914100	0.96935300
H	-6.95927300	-0.71908500	1.84873500
H	-7.79071100	-0.67816700	0.27668000
H	-7.97406700	-2.07393000	1.34343300
H	3.25755700	-3.43774300	-3.94668400
H	2.45663700	-2.10612500	-4.79185600
H	3.24062200	-1.83337300	-3.21616400
H	2.36790000	-4.36651200	-1.83458000
H	0.81605200	-3.83185700	-1.16359200
H	2.21512900	-2.75405900	-1.09468800
H	0.34475300	-3.56933600	-4.91355800
H	1.26763400	-4.83732000	-4.08391700
H	-0.29812900	-4.32721700	-3.44615600

H	-2.34197100	-5.08493600	2.16621700
H	-2.57724400	-3.34233000	2.40092200
H	-1.72369100	-3.92411800	0.96569300
H	-0.10281200	-6.11368900	2.43421900
H	1.34018200	-5.14247000	2.76288100
H	0.54172500	-5.04396900	1.18060500
H	0.16543800	-4.23350400	4.83806400
H	-1.45373100	-3.53333800	4.71656700
H	-1.22375500	-5.26738500	4.44038800
H	8.17192300	-0.56695100	-1.64965400
H	6.87349900	0.57517100	-2.02375900
H	7.66684500	0.61577700	-0.43528500
H	7.89474000	-2.38430900	-0.02128500
H	6.33900300	-2.69287700	0.76898600
H	7.29042600	-1.27489900	1.22866200
H	5.44082400	-1.39947800	-2.67320000
H	6.77331400	-2.49879200	-2.27034000
H	5.17256200	-2.74446700	-1.55175600

T-MeOCz-AQ

C	2.22864900	0.02199900	-2.94952000
C	2.32156800	-0.02299400	-1.57789400
C	1.18996800	-0.03978600	-0.72632800
C	-0.07610000	-0.00520800	-1.36153300
C	-0.16527500	0.03798400	-2.76391700
C	0.96002800	0.05098800	-3.55652600
C	1.30718000	-0.09229800	0.72510600
C	-1.34221000	-0.01139700	-0.60872600
C	-1.22434100	-0.04543100	0.84992200
C	0.05527900	-0.09146600	1.47178900
C	0.15663100	-0.13857600	2.87628300
C	-0.96567400	-0.13787000	3.67036100
C	-2.23524500	-0.08464800	3.07212200
C	-2.36386200	-0.03911400	1.69750800
O	-2.42081200	0.01161100	-1.22148700
O	2.42073600	-0.13419600	1.30300800
C	-6.01011300	3.15523000	-0.18362800
C	-4.79509100	3.48188300	0.44761200
C	-3.94629200	2.50097900	0.92974900
C	-4.32907100	1.17066200	0.76995400
C	-5.53893100	0.82754900	0.13206600
C	-6.39191300	1.82916900	-0.34485200
C	-4.42628300	-1.07123000	0.76471100
C	-4.16596900	-2.42592200	0.91330900
C	-5.09996000	-3.33544100	0.42506700
C	-6.27750200	-2.89344500	-0.20023700
C	-6.53402200	-1.53361900	-0.35028000
C	-5.60511200	-0.61335500	0.12677000
N	-3.68121500	0.01854500	1.17552800
H	0.88018800	0.08472900	-4.63862500
H	-1.16653700	0.06100600	-3.18327500
H	3.13546500	0.03514600	-3.54758000
H	1.15751700	-0.17479600	3.29395800
H	-0.87813100	-0.17457000	4.75260100

H	-3.14004600	-0.07581500	3.67222000
H	-3.00831300	2.75829100	1.41225600
H	-4.54263600	4.53340800	0.54368700
H	-7.32188000	1.55930700	-0.83341700
H	-7.45043900	-1.22341400	-0.84348700
H	-4.90127500	-4.39572000	0.53487900
H	-3.25460400	-2.76984200	1.39292100
N	3.64673800	-0.04421700	-1.01823200
C	4.36100400	1.06607800	-0.65369000
C	4.36911000	-1.16504100	-0.75110500
C	3.99348900	2.40474100	-0.73732600
C	5.61524900	0.65942000	-0.13091000
C	4.01295900	-2.50519300	-0.95199200
C	5.62140200	-0.79429900	-0.19358100
C	4.89855000	3.35913700	-0.29349900
H	3.02265500	2.68813100	-1.12710500
C	6.51057200	1.60647700	0.30684200
C	4.92782100	-3.46636500	-0.59213700
H	3.04534400	-2.76385700	-1.36551100
C	6.53565200	-1.76371600	0.16667800
C	6.14506100	2.97070700	0.22480800
H	4.62737000	4.40623800	-0.34413600
H	7.48161600	1.35588700	0.72110400
C	6.17968600	-3.11187200	-0.03599400
H	4.71382500	-4.52213400	-0.71433400
H	7.49187500	-1.49278600	0.59862300
O	7.07486300	3.82485000	0.67652500
O	6.97171200	-4.14552100	0.27164200
O	-6.74570700	4.22772100	-0.60499600
O	-7.23643800	-3.73414600	-0.69683800
C	-7.95686500	3.95175100	-1.25576100
H	-8.39071200	4.91872000	-1.51931800
H	-7.80213700	3.36381500	-2.17163700
H	-8.65590100	3.40927200	-0.60320400
C	-7.01142400	-5.11267600	-0.58058400
H	-7.87570900	-5.60213600	-1.03491200
H	-6.10163900	-5.42603000	-1.11243700
H	-6.93245300	-5.42874900	0.46960600
C	8.23489800	-3.89009100	0.85714100
H	8.12669200	-3.36649000	1.81372100
H	8.68528800	-4.86782400	1.02572200
H	8.87132300	-3.30538800	0.18289000
C	6.78790800	5.21080100	0.65810000
H	5.91258900	5.43961200	1.27634100
H	7.66768700	5.70066700	1.07479500
H	6.62102900	5.56545700	-0.36551900

C-MeOCz-AQ

C	-3.55318900	2.50273200	-1.55851000
C	-3.62513800	3.88698200	-1.59862000
C	-2.61433800	4.64991100	-1.02748000
C	-1.53636800	4.01602100	-0.41906000
C	-1.45916900	2.61043100	-0.34329000
C	-2.48880900	1.84435100	-0.92678000

C	-0.47620700	4.87853000	0.16522000
C	0.76290200	4.21395000	0.64043000
C	0.83720100	2.81398000	0.71846000
C	-0.38408900	1.99386000	0.47895000
C	1.86618300	5.01225900	0.93535000
C	3.07538200	4.42082800	1.27155000
C	3.17880200	3.03404800	1.29522000
C	2.07349100	2.22228900	1.03172000
O	-0.50037000	0.88532000	0.96589000
O	-0.59300700	6.09337000	0.19773000
N	2.28725000	0.82864900	0.97629000
N	-2.50836000	0.44649100	-0.92459000
C	-3.62299000	-0.31141800	-0.54963000
C	-3.34922100	-1.67760800	-0.77697000
C	-2.01098100	-1.74970900	-1.31793000
C	-1.51635000	-0.42817900	-1.38166000
C	3.04474000	0.23264800	-0.02452000
C	3.28383900	-1.11918200	0.31879000
C	2.65770900	-1.33542100	1.60393000
C	2.06362000	-0.11018100	1.98059000
C	-1.23913200	-2.81267900	-1.76654000
C	0.03290800	-2.55900000	-2.27431000
C	0.52791900	-1.24583000	-2.31989000
C	-0.24533000	-0.17438000	-1.87938000
C	-4.82414000	0.08884300	0.01946000
C	-5.76995100	-0.88576700	0.33151000
C	-5.51329100	-2.24342700	0.08896000
C	-4.29599200	-2.64308800	-0.46052000
C	2.56677900	-2.44014100	2.44451000
C	1.89404900	-2.31033100	3.65641000
C	1.31846900	-1.08234100	4.02440000
C	1.39776000	0.02642900	3.18848000
C	3.51774000	0.77322800	-1.21190000
C	4.24651000	-0.04996200	-2.06931000
C	4.49288900	-1.39107300	-1.73758000
C	4.01055900	-1.92985200	-0.54437000
H	-4.31693900	1.89741200	-2.03655000
H	-4.46101800	4.36871200	-2.09751000
H	-2.63130700	5.73451100	-1.04343000
H	1.74936300	6.08956900	0.87596000
H	3.94253300	5.03342800	1.49975000
H	4.12490100	2.54913800	1.51705000
H	-1.60292200	-3.83506900	-1.74217000
H	1.53214900	-1.04662100	-2.67710000
H	0.15874000	0.83168000	-1.93081000
H	-5.03073000	1.13221300	0.23533000
H	-6.70853000	-0.57388600	0.77519000
H	-4.11082200	-3.70084800	-0.61909000
H	3.00394800	-3.39876200	2.18343000
H	0.79206900	-0.98503000	4.96700000
H	0.93058000	0.96425900	3.46913000
H	3.33167100	1.81065800	-1.47328000
H	4.62221000	0.36589700	-2.99727000
H	4.21303800	-2.97225200	-0.31801000

O	-6.39232200	-3.24559700	0.36655000
O	0.71441800	-3.65087000	-2.71097000
O	1.84648800	-3.43227100	4.42828000
O	5.18195900	-2.25741300	-2.53603000
C	5.65115900	-1.77516300	-3.76952000
H	6.15388800	-2.61531300	-4.25183000
H	4.82772900	-1.43123300	-4.41120000
H	6.36882900	-0.95367400	-3.63973000
C	1.13843800	-3.36218100	5.63931000
H	1.20265700	-4.35648100	6.08525000
H	1.58135800	-2.63076100	6.32972000
H	0.08194800	-3.10727000	5.47875000
C	1.96269800	-3.44627100	-3.32745000
H	2.29687700	-4.42976100	-3.66347000
H	1.87792800	-2.77850100	-4.19663000
H	2.69941800	-3.03577200	-2.62984000
C	-7.62280200	-2.89318600	0.94537000
H	-8.16845200	-3.82753600	1.08910000
H	-7.48888100	-2.40156600	1.91881000
H	-8.20735100	-2.23313600	0.28956000

tBuCz-PA

C	-4.94298800	0.76643200	-2.56463000
C	-4.76782300	0.75354500	-1.17043200
C	-3.48535800	0.59719400	-0.64119300
C	-2.31577300	0.45831200	-1.39323000
C	-2.49303900	0.47659600	-2.79049200
C	-3.77642000	0.62633400	-3.33000000
C	-3.62532200	0.61059400	0.75389600
C	-1.08076800	0.31004400	-0.65781700
C	-1.20095700	0.32518900	0.81321700
C	-2.43246100	0.47134700	1.54370700
C	-2.43980100	0.47337000	2.94437200
H	-3.39745800	0.58800700	3.44466500
C	-1.27325100	0.33517000	3.67324500
C	-0.05605100	0.19080400	2.99313500
C	-0.04254200	0.18871200	1.61254400
H	-5.91402900	0.87431500	-3.03697000
H	-3.87893200	0.63226700	-4.41295000
H	-1.29130600	0.33817100	4.75843500
H	0.87818600	0.08103900	3.53663600
N	-4.90650400	0.76504100	1.10885000
N	-5.58186800	0.87059600	-0.07304100
C	-7.01983300	0.90224900	-0.05771800
H	-7.35380000	1.47261800	-0.93319600
H	-7.31966900	1.45844300	0.83590400
C	-7.64172500	-0.49039800	-0.05627000
H	-7.29782100	-1.03513500	-0.94589300
H	-7.25721200	-1.03621400	0.81466200
O	0.04455400	0.17420900	-1.21423400
C	3.45011200	-3.30184500	-0.04857000
C	2.16109500	-3.50024200	0.46925400
C	1.34141000	-2.44322200	0.84917400
C	1.84783600	-1.15679700	0.69966000

C	3.14458500	-0.91440100	0.18406300
C	3.93463400	-1.97908500	-0.18500300
H	1.77711500	-4.50748900	0.57411900
H	0.34105100	-2.60402100	1.23435200
H	4.92887200	-1.80514700	-0.58557300
C	2.10362500	1.06595000	0.69729600
C	1.90281400	2.43426400	0.84409700
C	2.94116300	3.27651400	0.46240400
C	4.15135900	2.78926100	-0.05458500
C	4.32256300	1.39067600	-0.18839000
C	3.31135800	0.53412300	0.18249400
H	0.96529800	2.81900000	1.22879200
H	2.79621000	4.34487200	0.56523200
H	5.25125700	0.99459800	-0.58822600
N	1.24645600	0.03897700	0.99172200
H	-1.62960600	0.37155800	-3.43876700
C	5.28630600	3.71213000	-0.48282100
C	4.34532700	-4.45933900	-0.47532700
C	5.57860200	3.48642200	-1.97544300
H	6.39361300	4.14379200	-2.29899100
H	5.88013400	2.45482200	-2.18369500
H	4.69601600	3.70778000	-2.58446800
C	6.54156500	3.38349200	0.34189000
H	7.36824400	4.03592600	0.03874700
H	6.35910200	3.53681500	1.41096000
H	6.86671100	2.34765200	0.20044400
C	4.94895900	5.18958700	-0.27694800
H	4.07734900	5.49436800	-0.86630000
H	4.75535600	5.42102200	0.77634200
H	5.79599600	5.80403400	-0.59915100
C	3.68049600	-5.82097200	-0.26787000
H	3.43923600	-6.00105600	0.78561500
H	2.76238300	-5.91993500	-0.85712200
H	4.36536800	-6.61253700	-0.58913800
C	5.64239600	-4.42399800	0.34938100
H	6.29901400	-5.24771600	0.04720900
H	6.19459500	-3.48941200	0.20685500
H	5.42983900	-4.53054700	1.41857900
C	4.68149200	-4.30801300	-1.96809000
H	3.77178300	-4.32349000	-2.57723800
H	5.20992600	-3.37237900	-2.17745800
H	5.32558700	-5.13400400	-2.29050100
C	-9.16423100	-0.45005700	-0.02514000
H	-9.49502400	0.10564400	0.86347700
H	-9.53251700	0.11636700	-0.89236700
C	-9.78932700	-1.83890600	-0.02092100
H	-9.46162000	-2.41422100	0.85277100
H	-10.88327600	-1.78850900	0.00248700
H	-9.49912700	-2.40348200	-0.91475500

DMAC-PA

C	3.97250000	-0.99658700	-2.41963100
C	3.74908300	-0.81469400	-1.04436000
C	2.44958400	-0.59474800	-0.58402100

C	1.30756100	-0.54721100	-1.38653300
C	1.53328900	-0.73479100	-2.76411100
C	2.83379800	-0.94954900	-3.23657100
C	2.53933700	-0.43946500	0.80580000
C	0.04684600	-0.31073500	-0.72182400
C	0.11189700	-0.14622900	0.74719000
C	1.32080600	-0.20513700	1.53007300
C	1.28903300	-0.03929100	2.92118600
H	2.23234600	-0.09520900	3.45734800
C	0.10418800	0.18742600	3.59536700
C	-1.09159300	0.25180600	2.86551000
C	-1.06478200	0.08835400	1.49653900
H	4.95867600	-1.16161400	-2.84133200
H	2.97369900	-1.08676300	-4.30653400
H	0.09087100	0.31440700	4.67312000
H	-2.04153900	0.42851000	3.36249300
N	3.80653700	-0.55041200	1.22277100
N	4.52292900	-0.79811500	0.08786400
C	5.95951900	-0.82943100	0.15849600
H	6.32084500	-1.50361600	-0.62773700
H	6.22423300	-1.27178400	1.12386300
C	6.58825000	0.55225300	0.01076200
H	6.27827400	0.98393300	-0.95048300
H	6.17684100	1.20142500	0.79410200
O	-1.05128200	-0.24594700	-1.34364700
H	0.69316700	-0.70862400	-3.44985500
N	-2.33767800	0.16607200	0.80681700
C	-2.77452300	1.39204300	0.36228200
C	-3.07035300	-0.98566300	0.64024200
C	-3.94774500	1.48986100	-0.42173500
C	-2.04238700	2.54799900	0.70777700
C	-4.25427400	-0.97214800	-0.13375400
C	-2.63019900	-2.17482900	1.25990300
C	-4.35486900	2.75995500	-0.81518100
C	-2.47997700	3.78984500	0.30272300
H	-1.13622500	2.44514600	1.28882700
C	-4.96758900	-2.16107900	-0.23966700
C	-3.36654200	-3.33239200	1.13530900
H	-1.71002900	-2.16515400	1.82783400
C	-3.64590500	3.90176200	-0.45746900
H	-5.23952700	2.86965800	-1.43215000
H	-1.91223500	4.67399100	0.57309300
H	-5.86967100	-2.19065200	-0.84024500
C	-4.54602600	-3.32849100	0.38785500
H	-3.02211700	-4.24240000	1.61529800
H	-3.99644200	4.87635700	-0.78264100
H	-5.13100200	-4.23690400	0.28281600
C	-4.64930400	0.25067200	-0.93150600
C	-6.17292400	0.43987000	-0.93589000
H	-6.46068800	1.28872900	-1.56021300
H	-6.67511900	-0.43313300	-1.35874700
H	-6.55642600	0.60597100	0.07558800
C	-4.14016500	0.01780300	-2.38059300
H	-3.05491800	-0.11776600	-2.38578900

H	−4.61836600	−0.87525200	−2.79742700
H	−4.39994200	0.88107400	−3.00289600
C	8.10855500	0.51452600	0.09976100
H	8.40559200	0.07203300	1.06090800
H	8.50356600	−0.15453000	−0.67795100
C	8.74021000	1.89283900	−0.04487800
H	8.38569800	2.57148800	0.73963600
H	9.83239500	1.84461400	0.02261100
H	8.48397600	2.34354500	−1.01084300
